# Conference Proceedings for the 10th Annual Meeting of Arthroplasty Society in Asia (ASIA), 26th Annual Meeting of the Thai Hip and Knee Society (THKS), and the 16th Annual Meeting of the ASEAN Arthroplasty Association (AAA)

**DOI:** 10.1186/s42836-024-00286-8

**Published:** 2024-12-20

## O1 Application of 3D-printed individualized articulating spacer in two-stage revision for periprosthetic joint infection after total knee arthroplasty

### Liqiang Zhi, Xiangxiang Sun, Jianbing Ma, Liang Du

#### Department of Joint Surgery, Honghui Hospital, Xi’an Jiaotong University, Xi’an, China

##### **Correspondence:** Liqiang Zhi (zhiliqiang2011@126.com)

*Arthroplasty 2024*, **6(Suppl 1):**O1

**Background and Aims**

PJI is one of the most serious complications of total knee arthroplasty. The current “golden standard” for treating PJI is two-stage revision, and the design and production of bone cement spacers are essential. Currently, various spacers have shortcomings for revision surgery. Therefore, better restoring the affected limb’s function during the infection interval is a challenge in the clinical application of spacers. 

**Methods**

A prospective analysis was conducted on 20 patients with PJI who were admitted to the Joint Department of Xi’an Honghui Hospital from October 2021–March 2023. All PJI patients underwent two-stage revision surgery. 10 patients were treated with 3D-printed customized spacer molds (3D printing group), and the other 10 patients were treated with existing spacer molds (Traditional group). Preoperative CT scans of the infected knee were performed on patients in the 3D group and imported into image processing software (Mimics 21.0). 3D printing technology was used to accurately replicate the CT data of the original prosthesis. The original prosthesis data were mirror-reversed and modeled to obtain a 3D-printed mold consistent with the original prosthesis. The mold was then cut and polished to obtain a spacer assembly mold that was completely identical to the original prosthesis (Fig. 1). Antibiotics were administered postoperatively based on the results of bacterial drug sensitivity testing. The operation time, blood loss, preoperative and postoperative VAS scores, postoperative KSS scores, and ROM of the affected limb were recorded in both groups to evaluate the advantages and disadvantages of the two methods.

**Results**

A total of 20 patients with PJI were included, including 10 patients in the 3D printing group and 10 patients in the traditional group (Table 1). All patients were followed up until the time of the second revision surgery. The study found that patients in the 3D printing group had significantly improved postoperative pain VAS scores, postoperative KSS scores, and ROM of the affected limb compared to the traditional group. There was no significant difference in infection control rates between the two groups, but the waiting time during the PJI interval was significantly shorter in the 3D printing group (Table 2).

**Conclusion**

The use of 3D-printed spacer molds can significantly shorten the operation time, reduce blood loss, and improve pain and function in the affected limb. It also shortens the time limit for the PJI interval, providing more favorable conditions for the two-stage revision surgery.

**Table 1 (Abstract O1):** Patient baseline data and data during follow-up time

	Gender (F/M)	Age (years ± x)	Follow-up time (week ± x)
3D Printing Group	3/7	65.3 ± 3.2	26.1 ± 2.8
Traditional Group	5/5	66.5 ± 4.5	24.2 ± 3.4
*P* value	>0.05	>0.05	>0.05

**Table 2 (Abstract O1):** Comparison of various indexes between the two groups

	VAS (± x)		Operation Time	Blood loss	KSS Scores (± x)		ROM (°± x)		Reinfection	Interval Time
	Pro-op	Pos-op	(min ± x)	(mL ± x)	Pro-op	Pos-op	Pro-op	Pos-op		(weeks ± x)
3D Printing Group	5.35 ± 1.34	1.91 ± 0.59	121.46 ± 12.60	452.32 ± 32.45	38.93 ± 8.01	77.96 ± 9.74	68.89 ± 9.44	93.48 ± 7.45	1	9.36 ± 2.60
Traditional Group	5.84 ± 1.32	2.31 ± 1.12	188.67 ± 15.43	594.76 ± 45.01	34.25 ± 6.31	67.83 ± 8.31	66.50 ± 10.48	80.08 ± 5.89	1	14.83 ± 1.12
*P* value	>0.05	>0.05	<0.05	<0.05	>0.05	<0.05	>0.05	<0.05	>0.05	<0.05

**Fig. 1 (Abstract O1):**
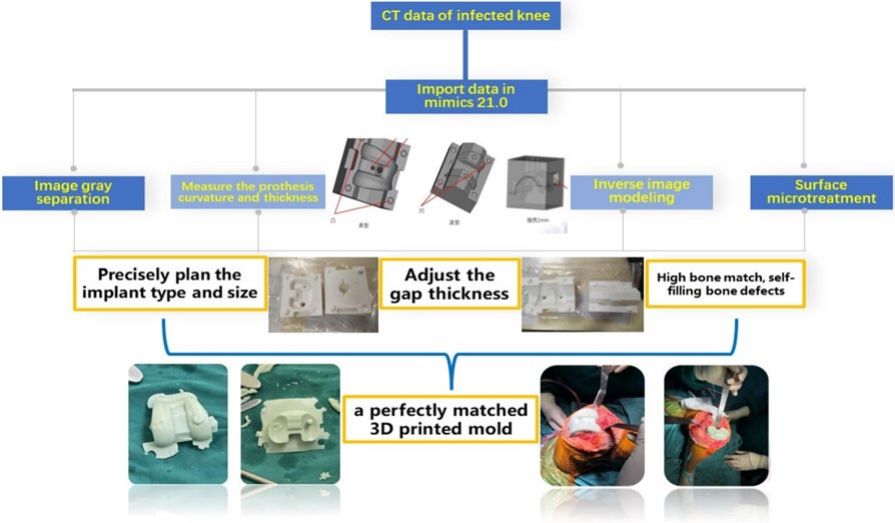
The operation procedure of 3D-printed, individualized spacer mold

## O2 Diagnosis of periprosthetic joint infection: the impact of intraoperative direct sonication on time to positivity

### Wenbo Mu^1^, Chen Zou^1^, Tuerhongjiang Wahafu^1^, Wentao Guo^1^, Boyong Xu^1^, Javad Parvizi^2,3^, Li Cao^1^

#### ^1^Department of Orthopaedics, First Affiliated Hospital of Xinjiang Medical University, Urumqi, China; ^2^International Joint Center, Acibadem University Hospital, Istanbul, Turkey; ^3^Rothman Orthopedic Institute at Thomas Jefferson University Hospital, Philadelphia, PA, USA

##### **Correspondence:** Wenbo Mu (muwenbo8964@163.com)

*Arthroplasty 2024*, **6(Suppl 1):**O2

**Background and Aims**

This study aimed to evaluate the impact of intraoperative direct sonication on the yield of traditional culture and the time to positivity (TTP) of cultures obtained for periprosthetic joint infection (PJI), thereby assessing its potential to improve diagnostic efficiency and reduce contamination risk.

**Method**

A prospective cohort study was conducted at a tertiary care center, involving 190 patients undergoing revision surgery for PJI from August 2021 to January 2024. Patients were included based on the 2018 International Consensus Meeting definition of PJI. The study utilized a novel sonication protocol, which involved direct intraoperative sonication of the implant and tissue, followed by incubation in a BACT/ALERT 3D system. The primary outcomes measured were the number and percentage of positive culture samples, identified microorganisms, and the TTP of each culture. Statistical analysis was performed using R software, with various tests applied to assess the significance of the findings.

**Results**

The study included 510 positive cultures from 190 patients, demonstrating that sonication significantly improved the positivity rate for both tissue and prosthesis specimens (*P* < 0.05). The median TTP for all samples was 3.13 days, with sonicated samples showing a significantly shorter TTP compared to non-sonicated samples (*P* < 0.05). Specifically, the shortest median TTP was observed in prosthesis post-sonication samples. Furthermore, the study found that Gram-positive organisms had a shorter TTP than Gram-negative organisms, and specific microorganisms like Staphylococcus aureus and MRSE showed the fastest TTP. The analysis also revealed higher positivity rates in chronic PJIs compared to acute PJIs for sonicated tissue samples (Fig. 1).

**Conclusions**

The study demonstrates that intraoperative direct sonication combined with the BACT/ALERT 3D system can significantly enhance the diagnostic yield of cultures and reduce the TTP for common PJI pathogens. This novel technique not only improves pathogen detection, facilitating the tailoring of antibiotic therapy but also potentially reduces the risk of contamination associated with sonication. These findings suggest that direct intraoperative sonication could be a valuable addition to the current diagnostic protocols for PJI, contributing to more effective management and treatment of this complex condition. Further research is necessary to explore the clinical significance of TTP and its correlation with patient outcomes in PJI.

**Fig. 1 (Abstract O2):**
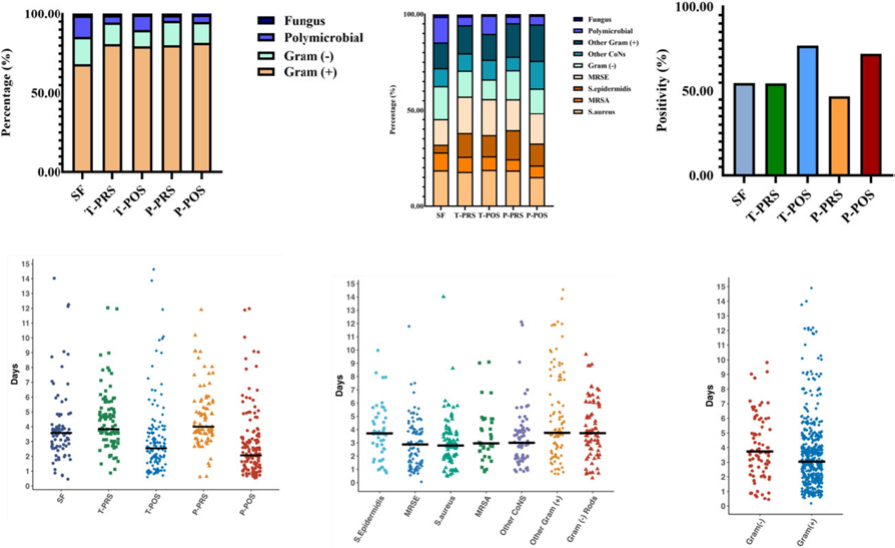
Study result

## O3 How often bacteraemia occurs in patients with chronic periprosthetic joint infection: a prospective, observational cohort study

### Baochao Ji, Yichang Li, Guoqing Li, Li Cao

#### Department of Orthopaedics, First Affiliated Hospital of Xinjiang Medical University, Urumqi, China

##### **Correspondence:** Baochao Ji (jbcjoint@126.com)

*Arthroplasty 2024*, **6(Suppl 1):**O3

**Background and Aims**

Periprosthetic joint infection (PJI) poses a formidable challenge following total joint arthroplasty (TJA), especially for chronic infection with a mature biofilm. The prevalence and impact of bacteremia in chronic PJI remain unknown. The purposes of this study are to evaluate the prevalence of bacteremia and the influence of positive blood cultures on the treatment success of one-stage revision in patients with chronic PJI.

**Methods**

A prospective observational cohort study was conducted from June 2021 to May 2022. To eliminate the influence of false positives in the culture process, the primary TJA cohorts were set as the control group and the PJI cohorts as the observation group. A standard set of two BACT/ALERT blood culture bottles (anaerobic and aerobic) defined one culture, and two cultures were routinely obtained per patient in both groups. The influence of positive blood cultures on surgical outcomes, vital signs, and systemic inflammatory factors was assessed (Table 1).

**Results**

Among 125 initially included patients, 108 met the criteria (56 chronic PJI, 52 primary TJA). A 19.6% rate of positive blood cultures in chronic PJI did not significantly differ from the primary TJA group (21.2%). Among all the PJI patients with positive blood cultures, no consistent organism was identified between the preoperative blood culture result and intraoperative tissue culture. Predominant organisms of blood culture are Staphylococcus hominis and Staphylococcus epidermidis, hinting at potential contamination. There was no significant difference in the Kaplan-Meier survivorship for infection-free implant survival at 27 months between chronic PJI patients with positive blood cultures and culture-negative results (88.9% vs 93.5%; *P* = 0.929) (Fig. 1). 

**Conclusion**

Bacteremia is rare in chronic PJI, suggesting chronically infected prostheses are unlikely sources of disseminated bacteremia and will not affect treatment outcomes.

**Fig. 1 (Abstract O3):**
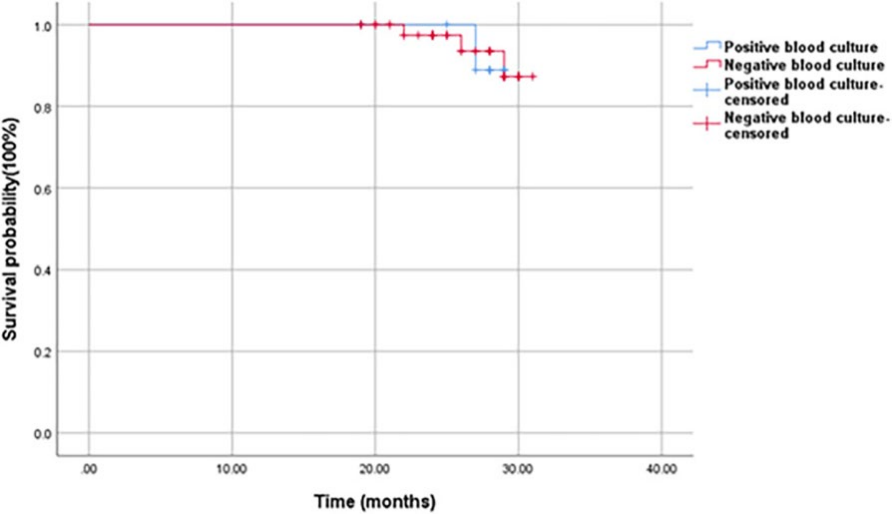
The infection-free survival of implants between chronic PJI patients with positive blood cultures and culture-negative results by Kaplan-Meier survival analysis

**Table 1 (Abstract O3):** Preoperative blood culture data of the two patient cohorts

Organism, *n* (%)	Primary TJA (*n* = 52)	Chronic PJI (*n* = 56)	*P* Value
Culture negative	41 (78.8%)	45 (80.4%)	0.846
Staphylococcus cohnii	1 (1.9%)	1 (1.8%)	0.958
Staphylococcus hominis	6 (11.5%)	6 (10.7%)	0.892
Staphylococcus epidermidis	4 (7.7%)	2 (3.6%)	0.350
Peanibacillus alvei	0 (0.0%)	1 (1.8%)	N/A
Bacillus firmus	0 (0.0%)	1 (1.8%)	N/A

*TJA* total joint arthroplasty, *PJI* periprosthetic joint infection, *NAA* not applicable

## O4 Distribution of pathogen and antibiotics selection in patients with PJI

### Weijun Wang, Gongan Jiang, Minghao Zhang, Qing Jiang

#### Nanjing Drum Tower Hospital, The Affiliated Hospital of Nanjing University Medical School, Nanjing, China

##### **Correspondence:** Weijun Wang (drwilliamwang@163.com)

*Arthroplasty 2024*, **6(Suppl 1):**O4

**Background and Aims**

Periprosthetic joint infection (PJI) is a catastrophic complication of total joint arthroplasty. Identification of pathogens and sensitive antibiotics is critical in treating PJI. However, negative culture is not uncommon. Hence an understanding of the distribution of pathogens and their sensitivity to antibiotics would be helpful. The current study aimed to investigate the distribution of pathogens and their sensitivity to antibiotics in our center, to facilitate our further treatment for culture-negative patients and the acute cases when the pathogen has not been identified. 

**Method**

Patients diagnosed as PJI according to ICM 2018 criteria in our center were retrieved retrospectively. The distribution of Gram-stain bacteria, MRSA resistance, mixed infections, and multidrug resistance were analyzed. Based on sensitivity and coverage to antibiotics, parameters for effective coverage were constructed, and appropriate empirical antimicrobial usage schemes were provided.

**Results**

108 PJI cases with positive culture were included. 26 patients suffered polymicrobial infections, and 153 strains of pathogens were isolated, with the proportions of Gram-positive bacteria being 88.2%. 85 strains of multidrug-resistant bacteria were identified, accounting for 55.6%, with Gram-positive bacteria making up 88.3%, coagulase-negative staphylococci 54.9%, epidermal staphylococci 26.1%, and Staphylococcus aureus 15.7%, Gram-negative Enterobacteriaceae 5.2%, 61 strains of MRSA were detected, accounting for 39.9%, all resistant to penicillin and oxacillin, but sensitive to vancomycin, tigecycline, and linezolid with a 100% sensitivity rate; 14 strains induced clindamycin-resistant bacteria, accounting for 9.2%, Gram-negative Enterobacteriaceae showed a 100% sensitivity rate to imipenem and gentamicin, a 50% resistance rate to fluoroquinolones, and a 75.0% sensitivity rate to penicillin, cephalosporins, amikacin, and colistin. All MRSA strains were resistant to penicillin and oxacillin, while sensitive to vancomycin, tigecycline, and linezolid with a 100% sensitivity rate; the Gram-negative Enterobacteriaceae had a high resistance rate to fluoroquinolones of 50.0%.

**Conclusions**

Gram-positive bacteria, especially Staphylococci served as the most common pathogen in PJI in our center. Pathogens had a higher proportion of MRSA resistant to fluoroquinolone antibiotics. Vancomycin combined with imipenem is the best empirical anti-infection choice for PJI patients, while vancomycin combined with piperacillin/tazobactam, or cefotaxime can be considered as an alternative empirical medication.

## O5 The association between sinus tracts and treatment failure in patients with periprosthetic joint infection

### Bin-Fei Zhang, Zhi Yang

#### Department of Joint Surgery, Honghui Hospital, Xi’an Jiaotong University, Xi’an, China

##### **Correspondence:** Bin-Fei Zhang (zhangbf07@gmail.com)

*Arthroplasty 2024*, **6(Suppl 1):**O5

**Background and Aims**

To evaluate the association between preoperative sinus tract and relapse in patients with periprosthetic joint infection (PJI).

**Methods**

The patients with PJI were screened between Jan 2013 and Sep 2019. Demographic and clinical characteristics of patients were collected. Linear and nonlinear multivariate logistic regression models were used to identify the association between preoperative sinus tracts and relapse in patients with PJI. Analyses were performed using EmpowerStats and the R software.

**Results**

Two hundred twenty-four patients were included in this study. The mean age was 64.78 ± 12.51 years, and the mean follow-up period was 77.64 months. There were 124 PJIs in the knee and 100 PJIs in the hip. The median of the sinus tract was 1 (min 0–max 5). Thirty-six (16.0%) patients relapsed during the follow-up. The multivariate logistic regression models showed that the preoperative sinus tract was associated with failure (OR = 0.41, 95% CI: 0.20–0.85, *P* = 0.0174) after adjusting for confounding factors. The multivariate logistic regression model showed that the number of sinus tracts was also associated with relapsed (OR = 0.64, 95% CI: 0.42–0.96, *P* = 0.0307) after adjusting for confounding factors. In the subgroups of knee PJI (OR = 0.25, 95% CI: 0.09–0.74), *P* = 0.0125), the association between preoperative sinus tract and failure was more obvious than the hip PJI subgroup (OR = 0.64, 95% CI: 0.12–1.76), *P* = 0.3909). In the nonlinear association, one sinus tract was an inflection point for prediction. The one sinus tract was associated with relapse (OR = 0.41, 95%CI: 0.19–0.87, *P* = 0.0208), whereas there was no difference in relapse among two or more sinus tracts (OR = 0.89, 95%CI: 0.46–1.75, *P* = 0.7419). We found the nonlinear association was very stable in the propensity score-matching sensitive analysis (Fig. 1).

**Conclusion**

Preoperative sinus tracts were associated with relapse in patients with PJI, especially in knee PJI patients. Also, one sinus tract was already a risk indicator of relapse.

**Fig. 1 (Abstract O5):**
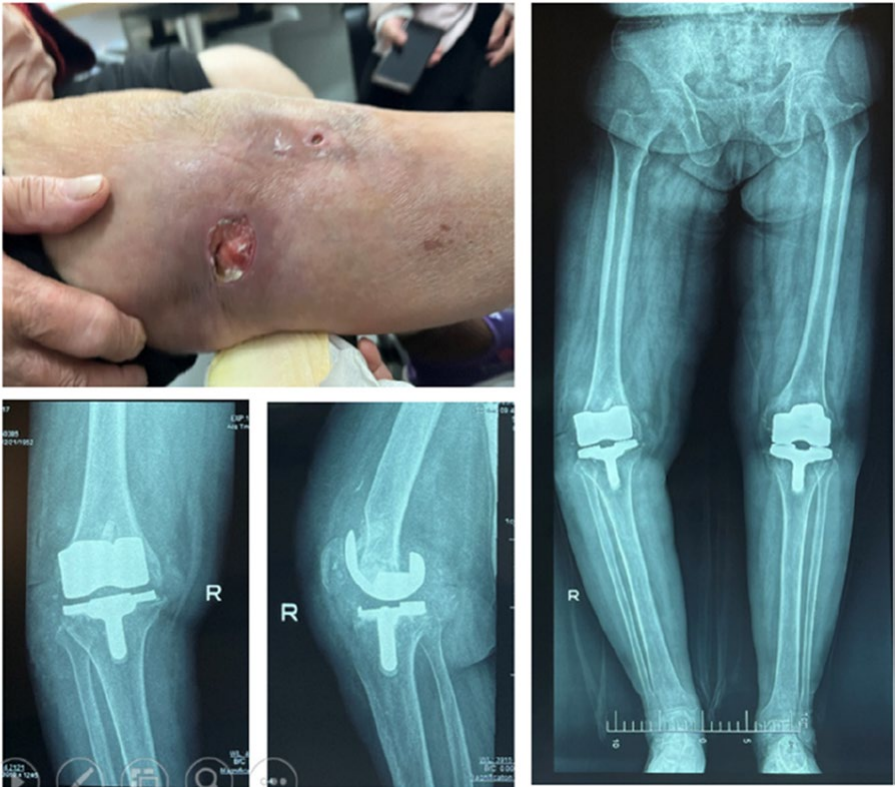
The case of preoperative sinus tracts

## O6 The iliofemoral impingement angle: a new radiographic measure

### Dirk van Bavel^1,2^, Sina Babazadeh^1,2^, Isaac Rhee^1,2^ and Jackson Ellis^1,2^

#### ^1^St Vincent’s Hospital Melbourne, Melbourne, Australia; ^2^Epworth HealthCare, Melbourne, Australia

##### **Correspondence:** Dirk van Bavel (dvanbavel@bigpond.com)

*Arthroplasty 2024*, **6(Suppl 1):**O6

**Background**

Hip dislocations after total hip arthroplasty (THA) are a debilitating complication. A common position for dislocation is with the hip in deep flexion. There is a large variation in the pelvic tilt of patients in different positions and those patients with increased anterior pelvic tilt in deep flexion are at increased risk of early impingement and hip instability.

New advances in three-dimensional modeling allow us to simulate the angle and position of impingement (which is typically bone-on-bone impingement of the femur on the anterior inferior iliac spine (AIIS)). This allows the identification of at-risk patients for early dislocation. This technology is expensive and not universally available and as such we describe a simple radiographic measurement that can replicate this impingement.

**Methods**

A radiographic study was performed on 117 consecutive patients who underwent preoperative planning for THA with 3D remodeling from 2021 to 2022 at a single institution. The deep flexion angle at which the femur first impinged on the pelvis was identified from the MAKO VROM 3D model and was recorded (see Fig. 1)—the 3D Maximal Flexion Impingement angle (3DMFIA). The Iliofemoral Impingement Angle (IFIA) is an angle measured with a vertical line up to the centre of the femoral head and the angle this subtends with a line rotated from the femoral head superiorly until it intersects with the pelvis (typically on the AIIS, see Fig. 2). The 3DMFIA was compared to the IFI angle and other radiologic measurements.

**Results**

The IFIa significantly correlated with the 3DMFIA (*r* = 0.69; 95% CI 0.55–0.78; *P* < 0.05). The 3DMFIA was compared to other radiological measurements used in the discussion of spinopelvic biomechanics and the correlation was poor to moderate (see Table 1). Of these, the Spinopelvic tilt was the best with a Pearson’s correlation of 0.53.

**Conclusion**

This study demonstrates that the novel radiographic measurement of the IFIa represented the deep flexion impingement angle of the femur on the ileum. The IFIa may be used as a simple and cost-effective alternative measurement to model impingement during deep flexion and hence identify patients who are at increased risk of impingement in deep flexion and instability.

**Fig. 1 (Abstract O6):**
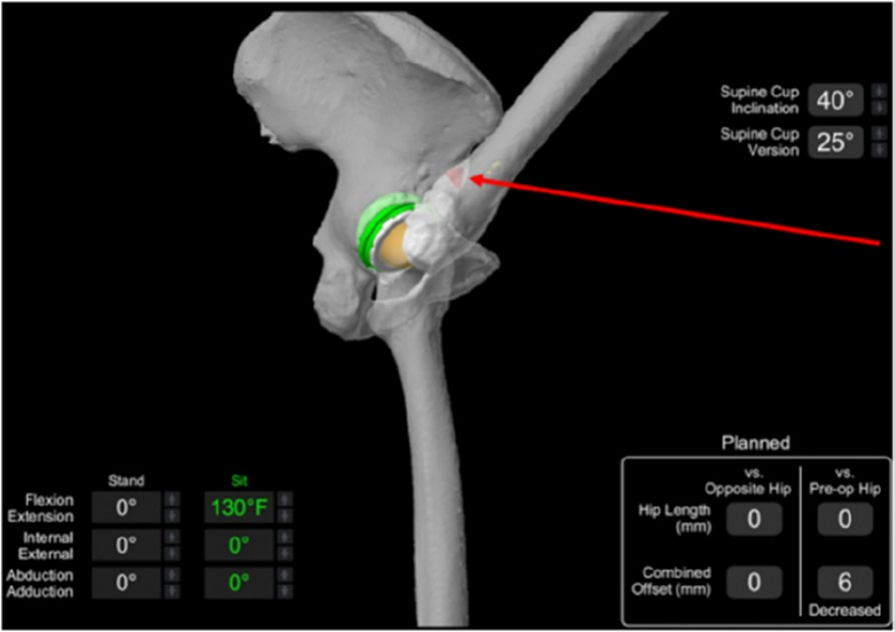
VROM planning of impingement in deep flexion. This shows impingement of the femur on the AIIS at 130 degrees of flexion

**Fig. 2 (Abstract O6):**
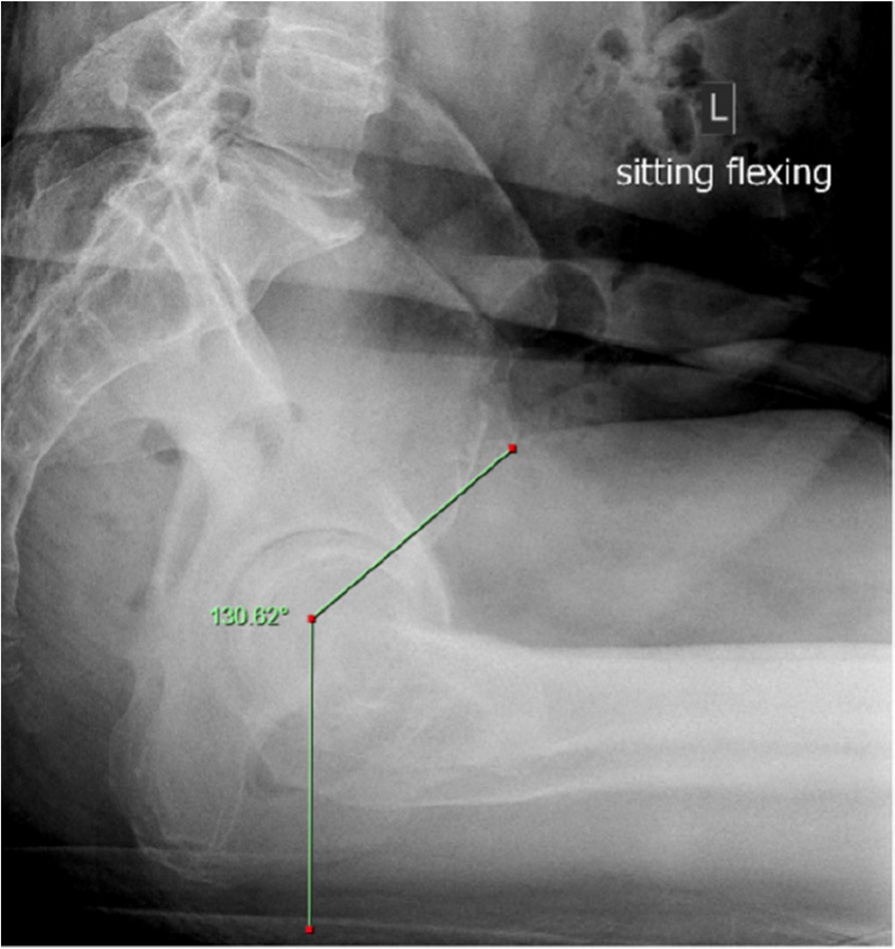
The Ilio Femoral Impingement angle IFIA is an angle measured with a vertical line up to the centre of the femoral head and the angle this subtends with a line rotated from the femoral head superiorly until it intersects with the pelvis (typically on the AIIS)

**Table 1 (Abstract O6):** Correlation between the 3D maximal Flexion Impingement Angle (3DMFIA) and pelvic radiologic measurements

Parameter (seated flexed)	*R*-value (95% CI)
IFIa	0.69 (0.55, 0.78)
SPT	0.51 (0.34, 0.65)
PI	0.22 (0.01, 0.41)
APPT	0.16 (−0.04, 0.36)
SS	−0.34 (−0.52, −0.14)

## O7 Development of artificial intelligence algorithm for differentiation of hip prosthesis

### Wiset Tangphromphan, Nattaphon Twinprai, Ritt Apinyankul, Prin Twinprai, Kittikhun, Tientong, Puripong Suttisopaphan

#### Department of Orthopedics, Khonkaen University, Khonkaen, Thailand

##### **Correspondence:** Wiset Tangphromphan (wiseta@kku.ac.th)

*Arthroplasty 2024*, **6(Suppl 1):**O7

**Background and Aims**

The increasing prevalence of revision total hip arthroplasty (THA) procedures, particularly among younger, active patient demographics, necessitates precise preoperative implant identification. Traditional methodologies employed for identification are both time-consuming and insufficient [1]. This study aims to explore the potential of deep learning for accurate preoperative identification of implant design.

**Methods**

We analyzed 2979 radiographs (anterior-posterior and lateral views) of total hip arthroplasty and bipolar hemiarthroplasty, 3 brands, and 5 stem designs from 647 patients. To accelerate and improve the accuracy of implant identification, we employed a YOLOV9 [2], object classification model. The model was trained and validated using separate datasets of the 1275 implants, 604 stems, and 1100 cup radiographs. The accuracy was compared to the implant data record.

**Results**

The CNN demonstrated exceptional performance in classifying hip arthroplasty components, achieving accuracies of 100% for implant models, 99.71% for stems, and 98.95% for cups. Interestingly, stem images yielded slightly higher accuracy than cup images (Figs. 1 and 2). 

**Conclusion**

This study demonstrates the effectiveness of deep learning with CNN for preoperative identification of hip arthroplasty implant designs. Both stem and cup images achieved high accuracy, suggesting either view can be utilized. This technology has the potential to streamline preoperative planning and improve outcomes in revision total hip arthroplasty surgeries.

**References**

1. Wilson NA, Jehn M, York S, Davis CM. Revision Total Hip and Knee Arthroplasty Implant Identification: Implications for Use of Unique Device Identification 2012 AAHKS Member Survey Results. J Arthroplasty. 2014 Feb; 29(2):251–5.
2. Redmon J, Divvala S, Girshick R, Farhadi A. You Only Look Once: Unified, Real-Time Object Detection [Internet]. Available from: http://pjreddie.com/yolo/

**Fig. 1 (Abstract O7):**
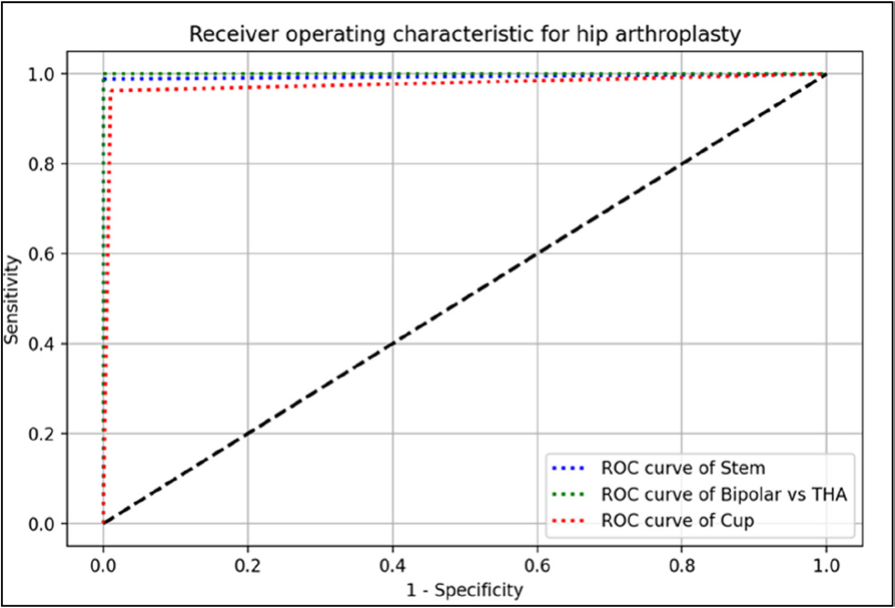
ROC Curve of the AI Model Prediction

**Fig. 2 (Abstract O7):**
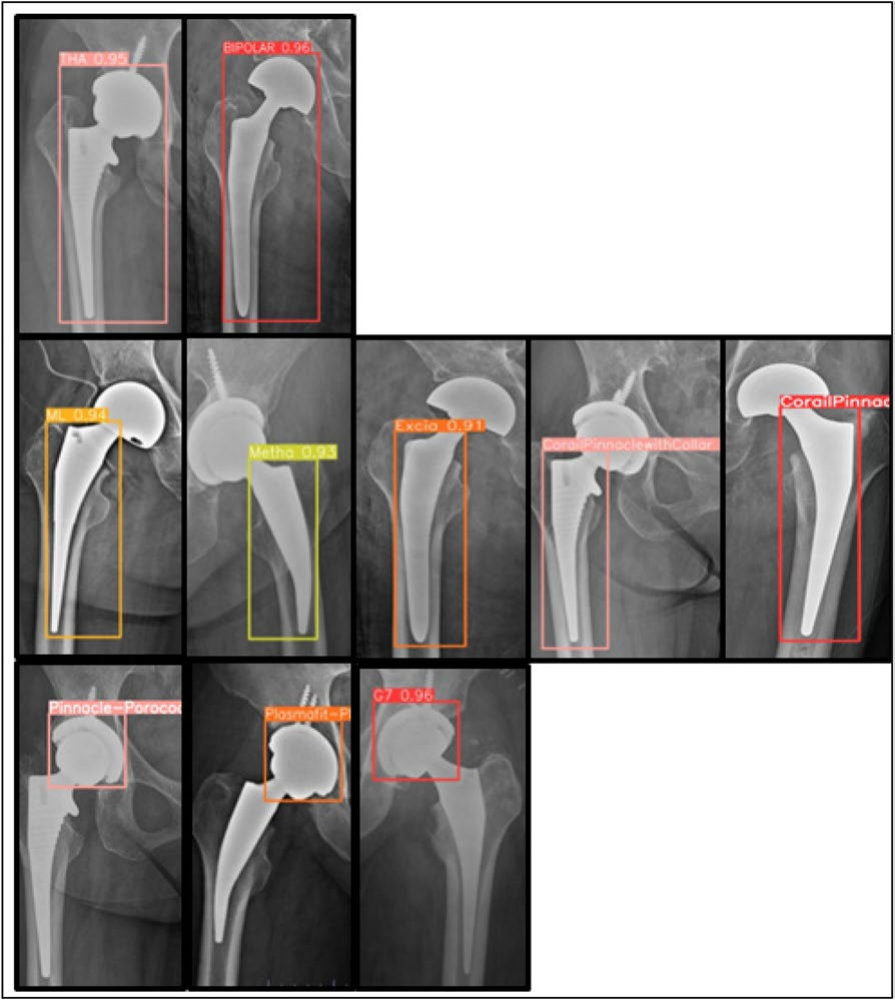
The X-ray image illustrating the AI-identified hip prosthesis

## O8 Revision total hip arthroplasty with pelvic discontinuity: the application of impaction bone grafting techniques

### Xinzhan Mao, Liang Xiong, Hui Li, Xianzhe Huang, Shuo Jie

#### Department of Orthopedics, The Second Xiangya Hospital of Central South University, Changsha, China

##### **Correspondence:** Xinzhan Mao (maoxinzhan72@126.com)

*Arthroplasty 2024*, **6(Suppl 1):**O8

**Background and Aims**

Pelvic discontinuity has been a considerable challenge in hip revision arthroplasty. Several techniques, including cup cage, custom triflange, and porous metal, have been reported in the literature while high surgical failure rates were observed [1]. In this study, we present the experience of our institution with the use of impaction bone grafting (IBG), titanium mesh, and cement fixation of polyethylene cups for the treatment of pelvic discontinuity during revision total hip arthroplasty (THA).

**Methods**

We retrospectively reviewed 5 patients (mean age 69 years; range, 65 to 75 years) who were admitted to our institution from January 2022 to March 2024 and underwent revision THA using IBG in combination with titanium mesh. Preoperative and post-revision Harris scores, VAS scores, and post-revision complications were evaluated. Radiographs were used to determine bone graft fusion and rotation center of the hip joint. 

**Results**

All 5 patients were presented with Paprosky type IIIB acetabular defect with pelvic discontinuity [2] (Fig. 1). Straight leg raise exercise was started on the second postoperative day, and partial weight bearing was allowed at 6 weeks after operation. These patients all reported significant improvement in their mobilization, Harris, and VAS scores post-operation. No implant migration or change in the center of rotation of the hip joint was observed in the last follow-up. The sequential radiographs taken at every follow-up showed good bone graft fusion with no bone resorption observed (Fig. 2). At the latest follow-up, no patient had re-revision or operations related to the prosthesis.

**Conclusion**

IBG in combination with cemented prosthesis is a biological reconstruction technique that could provide satisfying short-term outcomes in dealing with pelvic discontinuity of revision THA. However, long-term outcomes still need to be elucidated in further follow-ups.

**References**

1. Szczepanski et al., Surgical Treatment of Pelvic Discontinuity: A Systematic Review and Meta-Analysis. JBJS Rev. 2019 Sep;7(9):e4.
2. Paprosky et al. Acetabular defect classification and surgical reconstruction in revision arthroplasty: a 6-year follow-up evaluation. J Arthroplasty. 1994;9:33–44.

**Fig. 1 (Abstract O8):**
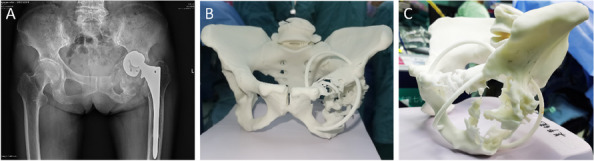
**A**, **B** and **C** Pictures showed preoperative pelvic X-ray and 3D reconstruction of one typical case

**Fig. 2 (Abstract O8):**
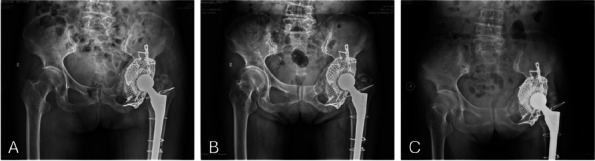
**A**, **B** and **C** Pictures showed postoperation, 3-month, and 6-month follow-up pelvic X-ray of the same case in Fig. 1

## O9 A retrospective study to determine ankylosing spondylitis patients’ satisfaction between dual-mobility and fixed-bearing THA in both fibrous and bony fusions

### Ahmad Obeid, Shuai Liu, Shuo Geng, Yan Zhang, XinHua Cai, LuJun Feng, Ke Bi

#### Department of Orthopaedic, First Affiliated Hospital of Harbin Medical University, Harbin, China

##### **Correspondence:** Ahmad Obeid (Dr.AhmadObeid@outlook.com)

*Arthroplasty 2024*, **6(Suppl 1):**O9

**Background and Aims**

The clinical significance of ankylosing spondylitis (AS) following total hip arthroplasty (THA) is receiving attention due to the various generally used surgical techniques based on both fibrous and bony hip fusions. This study examines the level of patient satisfaction among individuals with AS who have undergone primary THA. The study directly compares the utilization of dual-mobility (DM) and fixed-bearing (FB) prostheses.

**Methods**

A retrospective investigation was conducted by two groups of surgeons using the same surgical technique and approach to evaluate the level of satisfaction of a total of 100 hips afflicted with AS undergone THA between 2019 and 2023. The participants were categorized into two cohorts: 22 individuals underwent DM THA, whereas 29 individuals underwent FB THA. Additionally, a subgroup analysis was conducted, which included 31 cases of fibrous fusion and 20 cases of bony fusion. The patients were monitored for a duration of 6 months. The Oxford Hip Score (OHS), The Short Form 12 Health Survey (SF-12), Harris hip scores (HHSs), Bath Ankylosing Spondylitis Disease Activity Index (BASDAI) and X-rays were documented pre- and post-operation.

**Results**

The post-operative scores of all groups showed great improvement. DM THA revealed better improvement compared to FB THA. Significant differences were seen between fibrous fusion groups and bony fusion groups in terms of post-operative outcomes. Notably, bony fusion DM THA showed a greater improvement. The scores were evaluated with lower values indicating better performance and greater values indicating poorer performance. 

**Conclusion**

DM THA is a suitable substitute for conventional bearing surfaces in AS patients receiving primary THA. Patients in the bony fusion group had higher satisfaction levels, while also ensuring high patient satisfaction as indicated by Patient-Reported Outcome Measures (PROMs).

**Fig. 1 (Abstract O9):**
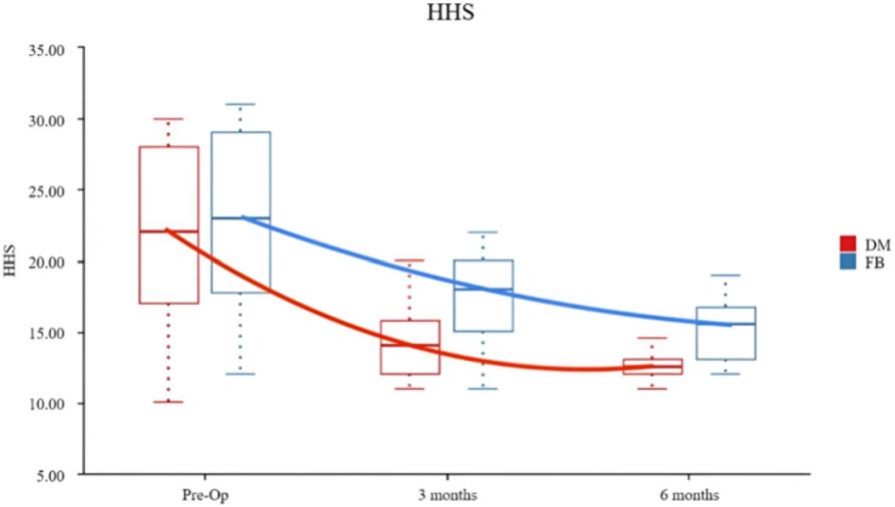
The HHS scores of both groups were compared at different periods

**Fig. 2 (Abstract O9):**
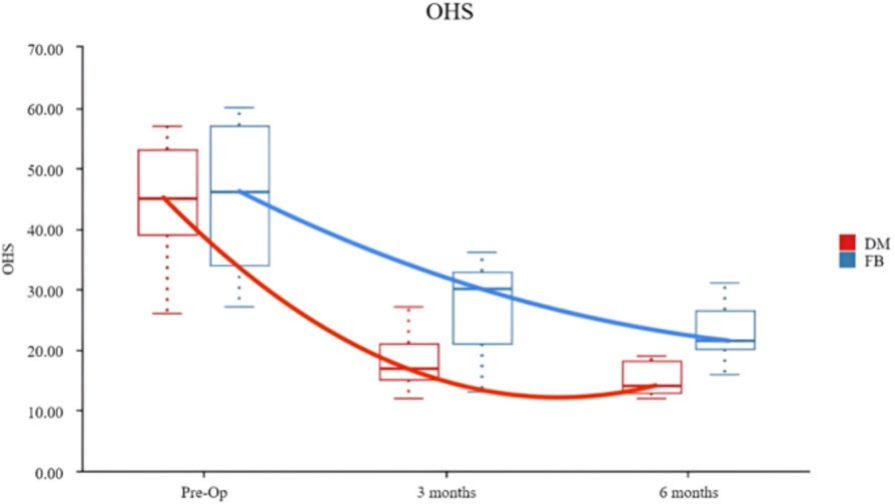
The OHS scores of both groups were compared at various time intervals

## O10 Application of intraoperative individualized cup combination—cup-on-cup technique in severe acetabular defects

### Weihua Li^1,2^, Daobi Liu^3^, Xuqiang Liu^1,2^, Xiaofeng Li^1,2^

#### ^1^Orthopedic Hospital, The First Affiliated Hospital, Jiangxi Medical College, Nanchang University, Nanchang, China; ^2^Artificial Joints Engineering and Technology Research Center of Jiangxi Province, The First Affiliated Hospital, Jiangxi Medical College, Nanchang University, Nanchang, China; ^3^Lushan Rehabilitation and Nursing Center, Jiujiang, China

##### **Correspondence:** Xiaofeng Li (doctorli00001@163.com)

*Arthroplasty 2024*, **6(Suppl 1):**O10

**Background and Aims**

In the case of severe acetabular defects in revision total hip arthroplasty (THA), it is always a challenge for the surgeon to fill the bone defect, restore the hip center of rotation (COR), and obtain the initial stability of the revision cup. This study aimed to investigate the application of the cup-on-cup technique in revision THA and report clinical and radiographic outcomes from a series of case follow-ups.

**Methods**

Retrospective analysis of 10 patients who underwent acetabular prosthesis revision with cup-on-cup technique in our center from February 2018 to June 2019. According to the Paprosky classification of acetabular bone defects, there were 2 cases of type II C, 3 cases of type III A, and 5 cases of type III B. All acetabular revisions were performed by the same senior joint surgeon, and relevant clinical and radiographic data were systematically collected during follow-up. The patient gave their informed written consent to publish their information in an open access journal.

**Results**

The average follow-up was 54.8 ± 5.1 months, and the Harris score of the hip joint increased from 37.0 ± 9.9 preoperatively to 80.5 ± 3.1 postoperatively at the final follow-up (*P* < 0.001). Comparing the surgical side’s hip COR to the contralateral side, the preoperative average upward displacement was 33.8 ± 15.0 mm, while the postoperative average upward displacement was 0.2 ± 3.3 mm (*P* < 0.001). Similarly, the preoperative average inward displacement was 9.1 ± 5.1 mm, while the postoperative average outward displacement was 1.8 ± 1.6 mm (*P* < 0.001). There was no significant difference (*P* = 0.71) between the average density values of the contralateral and surgical sides at the final follow-up, which were 127.4 ± 13.7 and 125.0 ± 14.8, respectively. During the follow-up period, all patients achieved satisfactory radiographic outcomes, and no prosthetic loosening was observed (Fig. 1).

**Conclusion**

The cup-to-cup technique can reconstruct acetabular bone defects and restore hip COR in revision THA, with favorable clinical and radiographic outcomes.

**Fig. 1 (Abstract O10):**
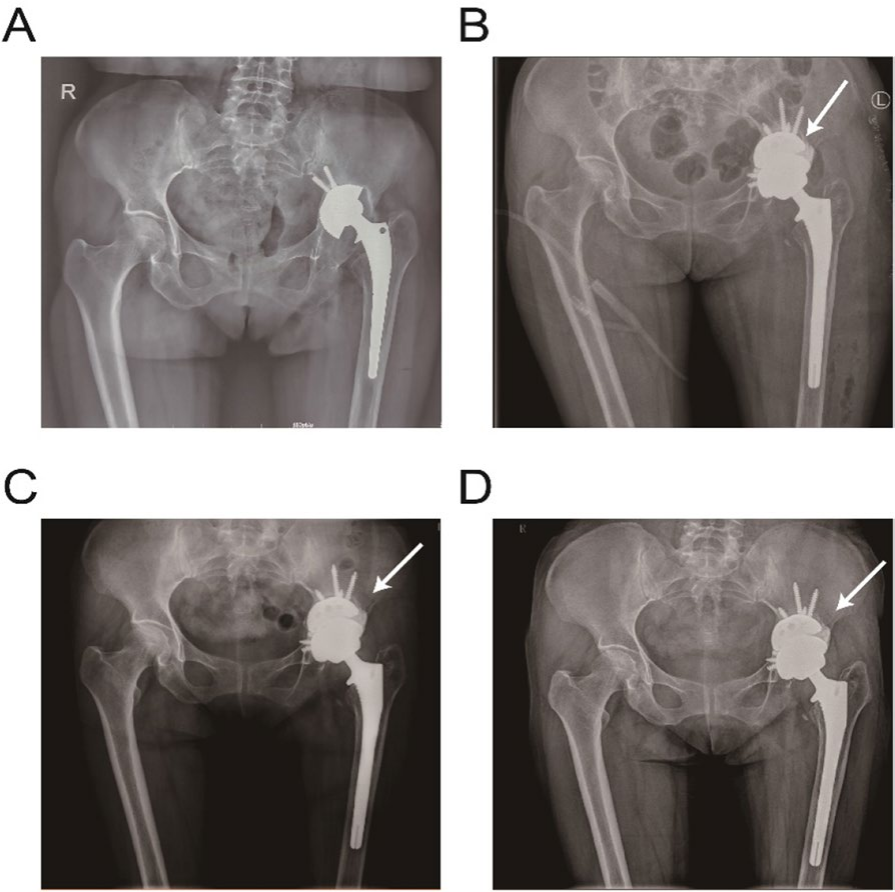
A 59-year-old female underwent THA 14 years ago due to avascular necrosis of the left femoral head. **A** Before the revision, the acetabular defect Paprosky III A was observed, and radiological bright lines were seen in areas 1 and 2 of the femoral prosthesis; **B** On the first day after revision, the prosthesis was in good position with a clear line of lacunar bone defect indicated by the white arrow; **C** One month after surgery, there was no loosening of the prosthesis, and the bright line was still visible; **D** Six months after the operation, the prosthesis did not loosen and bone integration was obtained

## O11 The comparative study on the efficacy of posterior lateral spare approach with preservation of the external rotation muscle group and data approach in total hip arthroplasty for garden type IV femoral neck fractures in the elderly

### Ye Ye

#### Department of Adult Reconstruction, Henan Provincial Orthopedic Hospital, Zhengzhou, China

*Arthroplasty 2024*, **6(Suppl 1):**O11

**Background and Aims**

Total hip arthroplasty is the best choice for the treatment of displaced femoral neck fractures in the elderly. The posterior lateral approach is proficiently used by the vast majority of doctors and is one of the most commonly used approaches. However, it inevitably carries a high risk of dislocation when used for total hip replacement in elderly femoral neck fractures. The currently popular Direct Anterior Approach (DAA) is believed to greatly improve the risk of dislocation, but due to its long learning curve, it has not been widely applied. The author applied the SPAIRE approach (Sparing Piriformis And obturator Internus Repair obturator Externus), a minimally invasive posterior lateral approach that preserves the short external rotator muscle group, for minimally invasive total hip arthroplasty in the treatment of femoral neck fractures in the elderly, and compared it with another group of patients who underwent total hip arthroplasty using the DAA approach at the same center, and observed its clinical efficacy.

**Methods**

A total of 96 elderly patients over 70 years old with femoral neck fractures who underwent hip replacement surgery at our hospital from October 2021 to May 2022 were selected as the study subjects and randomly divided into two groups. 48 patients were treated surgically through the posterior lateral SPAIRE approach, and 48 through the DAA approach. Postoperative observation and recording of surgical incision length, operation time, intraoperative blood loss, postoperative VAS scores, and other indicators were used to evaluate the clinical outcomes of the patients.

**Results**

Both groups of patients were able to perform active hip flexion exercises on the day of surgery and could stand or walk within 1 to 3 days postoperatively. The average surgical incision length for the DAA group was (12 ± 3.5) cm, the average operation time was (78 ± 16) minutes; the average intraoperative blood loss was (267 ± 58) mL; the VAS score at 3 days postoperatively was (2.1 ± 0.3). For the SPAIRE group, the average surgical incision length was (14 ± 2.5) cm, the average operation time was (68 ± 15) minutes; the average intraoperative blood loss was (342 ± 36) mL; the VAS score at 3 days postoperatively was (2.3 ± 0.4). There were no restrictions on patients’ body posture postoperatively in either group. Except for one patient lost to follow-up, the remaining patients received follow-up for 3 to 9 months. During the follow-up period, no case of joint dislocation occurred. The patients followed up postoperatively all had stable conditions and good recovery.

**Conclusion**

The SPAIRE minimally invasive posterior lateral approach, which preserves the short external rotator muscle group—piriformis, superior gemellus, obturator internus, and inferior gemellus, and repairs the tendon of the obturator externus, for total hip arthroplasty in the treatment of femoral neck fractures in the elderly, has good clinical efficacy compared to the DAA approach. The surgery has a small trauma and can preserve most of the external rotator muscle group of the hip joint, improving the stability of the patient’s hip joint and reducing postoperative pain. It is an effective clinical treatment method for elderly patients with femoral neck fractures who are at high risk of postoperative dislocation. Moreover, the learning curve for the SPAIRE approach is shorter than that for the DAA, making it more convenient for clinical promotion and application.

## O12 Does surgeon’s experience affect the radiographic diagnosis of Vancouver type B1 and B2 periprosthetic femoral fracture after hip arthroplasty?

### Chavarin Amarase^1^, Srihatach Ngarmukos^1^, Aree Tanavalee^1^, Saran Tantavisut^1^, Chotetawan Tanavalee^2^, Nonn Jaruthien^2^, Pakpoom Somrak^2^

#### ^1^Department of Orthopaedics, Faculty of Medicine, Chulalongkorn University, Bangkok, Thailand; ^2^Department of Orthopaedics, King Chulalongkorn Memorial Hospital, The Thai Red Cross Society, Bangkok, Thailand

##### **Correspondence:** Aree Tanavalee (areetana@hotmail.com)

*Arthroplasty 2024*, **6(Suppl 1):**O12

**Background and Aims**

Periprosthetic femoral fracture (PFF) is one of the most common complications of hip arthroplasty (HA). Vancouver classification is popular for classifying fractures and guiding treatment. However, diagnosing B1 and B2 remains challenging, with the surgeon's experience playing a pivotal role in decision-making. This study aims to assess how the surgeon’s experience affects the interpretation of radiographic characteristics for diagnosing Vancouver type B1 and B2 PFF after HA.

**Methods**

The Vancouver type B1 and B2 PFF patients who underwent fixation or revision surgery and had follow-up at least 1 year were included. Recorded radiographic characteristics for Vancouver-type diagnosis included comminution, stem subsidence, major osteolysis, fracture at the fixation level, cement or implant fracture, and implant type. Subsequently, the first to fourth-year orthopedics residents (group 1), orthopedists with less than 3 years of experience (group 2), and those with more than 3 years of experience (group 3) evaluated the radiographs and determined the Vancouver type B1 or B2 and the radiographic characteristics. The correctness of the Vancouver type and radiographic characteristics was compared within each orthopedist group.

**Results**

The study included 23 patients in the B1 group, 41 patients in the B2 group, 40 orthopedics residents in group 1, 20 orthopedists in group 2, and 20 orthopedists in group 3. Correctness rates for Vancouver type B1 and B2 were 65.8%, 73.1%, and 78.8% in groups 1, 2, and 3, respectively (*P* = 0.36). Surgeon experience significantly influenced four radiographic characteristics: cement or implant fracture (47.5%, 87%, and 75%; *P* < 0.01), implant type (40.8%, 70%, and 63.3%; *P* < 0.01), Stem subsidence (58.3%, 68.5, and 77.5%; *P* < 0.002), and major osteolysis (51.7%, 74.2%, and 73.3%; *P* ≤ 0.001).

**Conclusion**

The study highlights the impact of surgeon experience on radiographic characteristics when diagnosing Vancouver type B2 PFF, particularly in cases of cement or implant fracture, implant type, stem subsidence, and major osteolysis.

**Fig. 1 (Abstract O12):**
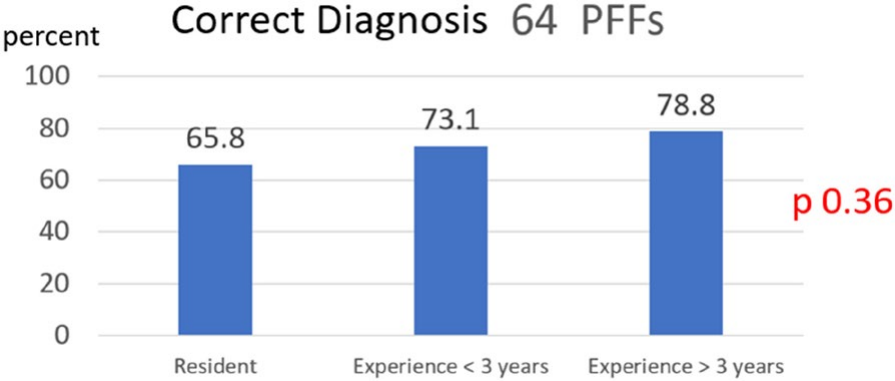
See text for description

**Fig. 2 (Abstract O12):**
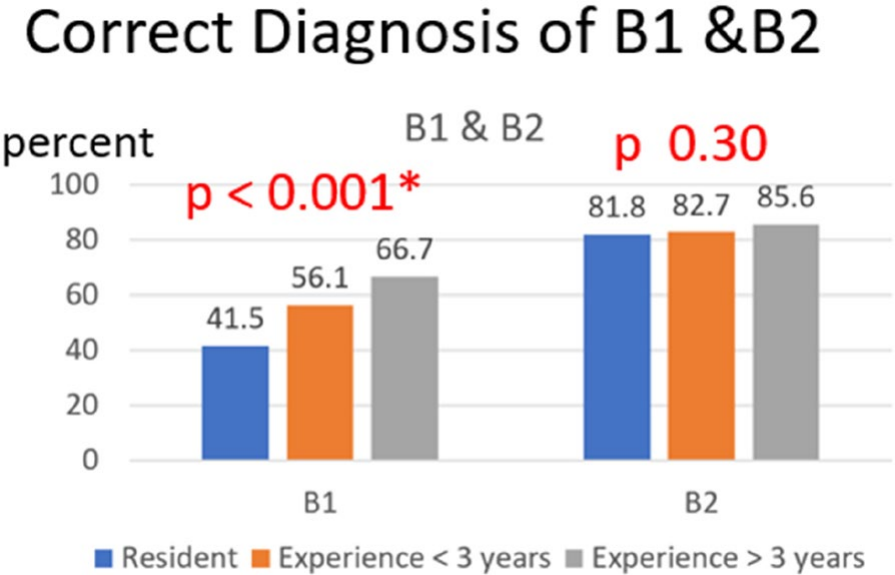
See text for description

## O13 Chronic expanding hematomas that occurred 10 years or more after primary total hip arthroplasty

### Chuanlong Wu, Hongyi Wang, Qiyuan Bao, Chuan He

#### Department of Orthopaedics, Shanghai Key Laboratory for Prevention and Treatment of Bone and Joint Diseases, Shanghai Institute of Traumatology and Orthopaedics, Ruijin Hospital, Shanghai Jiao Tong University School of Medicine, Shanghai, China

##### **Correspondence:** Chuanlong Wu (challengewu1988@163.com)

*Arthroplasty 2024*, **6(Suppl 1):**O13

**Background and Aims**

Chronic expanding hematoma (CEH) after total hip arthroplasty (THA) is a rare complication after joint replacement. This study aims to review the CEH patients after THA in our center to get more clinical experience [1].

**Methods**

In this study, we systematically reviewed the CEH patients after THA in our hospital in the past 20 years. The following data before surgery and during postoperative follow-up were collected: demographic information, initial prosthesis information, imaging data, and Harris scores. At the same time, the reports of CEH after THA were reviewed via literature.

**Results**

CEH occurred in 5 patients with more than 10 years of follow-up after THA, 1 case was caused by trauma, and 4 cases had no obvious cause. A total of 7 similar cases have been reported so far by reviewing the literature. 1) Inducement: Due to trauma or surgery, but not necessary. We speculated that it might be related to inadequate hemostasis in the initial operation. 2) Time: Slow progress for years. 3) Mechanism: Not clear. 4) Diagnosis: (1) MRI: T2-weighted phase shows a “Mosaic sign”—a mixture of fresh and old blood—indicating repeated bleeding. (2) Three typical histological features: peripheral wall wrapping with dense fibrous tissue; Fresh and mobile clots/blood; An intermediate area of loose connective tissue formation. 5) Differential diagnosis: Inflammatory pseudotumor, hemophilia, malignant tumor, etc. 6) Treatment: The gold standard is complete surgical resection (including capsule). CEH was characterized by progressive osteolysis. Therefore, once detected, follow-up should be conducted every 2–3 months, and removal of the mass should be considered as soon as bone loss is evident (Fig. 1).

**Conclusion**

CEH is a rare complication after THA that often progresses slowly for years. It should be distinguished from inflammatory pseudotumor, hemophilia, and malignant tumor. Once found, regular follow-up, early detection, and early treatment are needed.

**Reference**

1. Ishikawa T, Kawai T, Goda N, Goto K, Kuroda Y, Matsuda S: Chronic Expanding Hematomas That Occurred 20 Years or More After Primary Total Hip Arthroplasty: A Report of 2 Cases. *JBJS Case Connect *2021, 11(1).

**Fig. 1 (Abstract O13):**
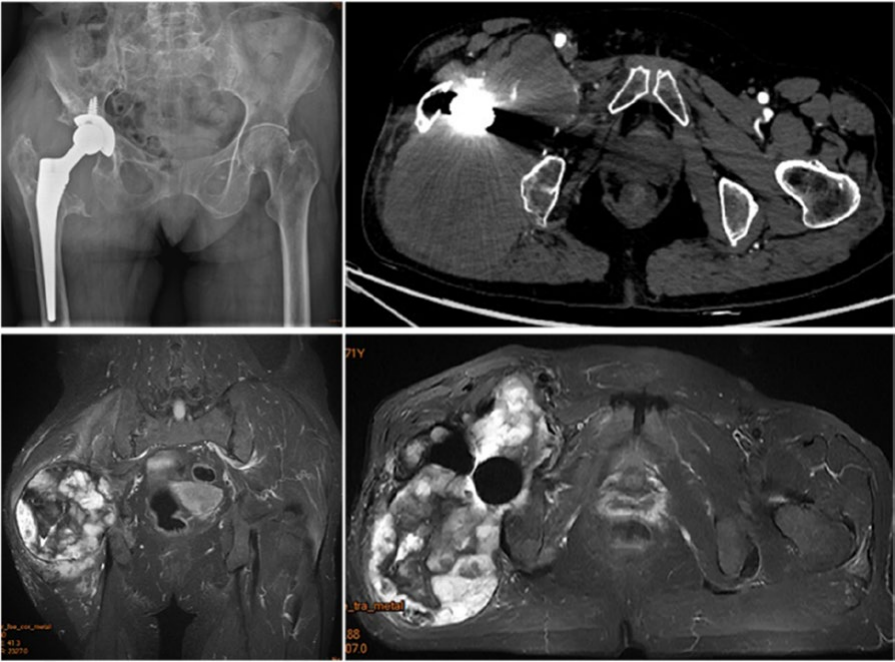
One CEH case: x-ray, CT, and MRI 20 years after primary THA

**Fig. 2 (Abstract O13):**
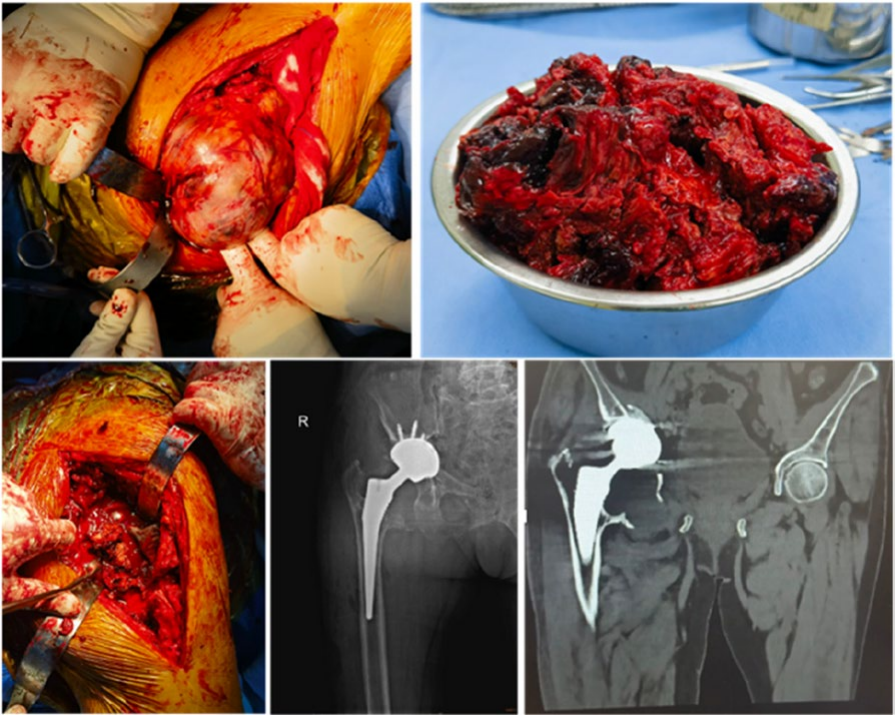
One CEH case: intraoperation and post operation

## O14 Constrained liner in revision total hip arthroplasty for patients with high-risk factors of dislocation

### Myeong Gu Lee, Young Soo Chun, Kee Hyung Rhyu, Yoon Je Cho

#### Department of Orthopedic Surgery, School of Medicine, Kyung Hee University, Seoul, Korea

##### **Correspondence:** Myeong Gu Lee (myeonggu.lee@khu.ac.kr)

*Arthroplasty 2024*, **6(Suppl 1):**O14

**Background and Aims**

Dislocation is one of the concerning complications after total hip arthroplasty (THA). There are several patient-related risk factors for dislocation, including neuromuscular diseases, psychiatric disorders, autoimmune diseases, and previous spinal surgery. Recurrent dislocations frequently occur in patients with these risk factors. Constrained liners can be used to prevent further dislocation, but re-dislocation and component failure have been reported. The purpose of this study is to investigate the results after revision THA using a constrained liner in patients with risk factors of dislocation.

**Methods**

A retrospective review of revision THAs using constrained liner between 2010 and 2022 was performed. A total of ten patients with minimum 2-year follow-up were included. Five Trilogy Longevity-constrained liners (Zimmer Biomet, Warsaw, IN, USA) and five Pinnacle ESC-constrained liners (DePuy Synthes, Warsaw, IN, USA) were used in revision THAs. The average age was 74 years, and the average follow-up period was 41.5 months. Risk factors included four autoimmune diseases, five psychiatric disorders, one sequelae of poliomyelitis, and five previous spinal surgeries. The patients experienced an average of 3.9 dislocations. All dislocations were non-traumatic. The patients were evaluated radiographically and clinically.

**Results**

No further dislocations occurred after the revision of THAs. There were no other complications, including loosening, osteolysis, and liner wear. The average inclination and anteversion angle of the acetabular cup before the revision were 44.1 degrees and 26.9 degrees, respectively. The average Harris hip score, UCLA score, and NRS pain score before revision were 52.4, 3.1, and 2.7, respectively. After the revision, the scores were 55.1, 3.1, and 2.5, respectively. There were no significant differences in the scores and range of motion before and after the revision. 

**Conclusion**

A constrained liner in revision THA provides satisfactory results in preventing further dislocation in patients with high-risk factors. Although poor results have been reported with traditional constrained liners, our study using modern constrained liners showed no re-dislocation or component failure.

## O15 Postoperative patient-reported pain and opioid consumption after total hip arthroplasty: a propensity score matched comparison of the direct superior and posterior approaches

### Seok Ha Hong, Seung Beom Han

#### Department of Orthopedic Surgery, College of Medicine, Korea University, Seoul, South Korea

##### **Correspondence:** Seok Ha Hong (terume00@gmail.com)

*Arthroplasty 2024*, **6(Suppl 1):**O15

**Background and Aims**

The direct superior approach (DSA), a muscle-sparing technique derived from the posterior approach (PA), has received little attention despite its potential advantages. As there is no consensus on the effect of DSA on postoperative pain and opioid consumption, this study compared the impact of DSA and PA on patient-reported pain and postoperative opioid consumption with medical and surgical complications. 

**Methods**

We reviewed 430 primary total hip arthroplasties for avascular necrosis and osteoarthritis between January 2016 and December 2022, categorized as DSA or PA. Demographic data, including age, sex, preoperative opioid usage, smoking status, chronic alcoholism, and underlying diseases were collected. Propensity score matching balanced DSA and PA groups. Patients who reported maximum and minimum pain on the postoperative day (POD), total opioid consumption, and chronic opioid use were compared between the two groups. Inflammation-related serum markers, medical, and surgical complications, length of hospital stay, and 90 days readmission rates were also analyzed.

**Results**

After matching, 131 patients were included in each group. Patients with DSA reported lower average maximum pain on POD #1 and #4, and lower average minimum pain on POD #1,2, and 4. DSA showed a significant reduction in opioid consumption and a shorter hospital stay. In addition, DSA showed a significant reduction in C-reactive protein (CRP) on POD #5, 14, and 28 after index surgery compared to PA. No significant differences were observed in chronic opioid use, medical complications, surgical complications, or 90-day readmission rates (Figs 1 and 2).

**Conclusion**

DSA was associated with slightly lower patient-reported pain and a marked reduction in opioid consumption. Furthermore, it provides earlier functional recovery and discharge from the hospital compared to PA.

**Fig. 1 (Abstract O15):**
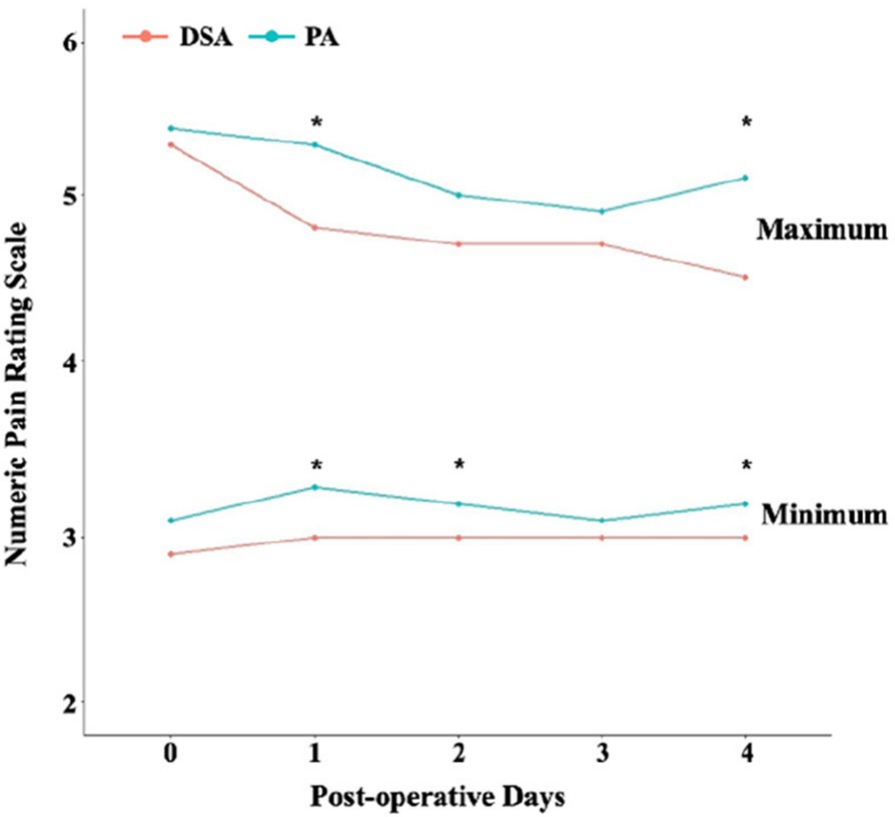
See text for description

**Fig. 2 (Abstract O15):**
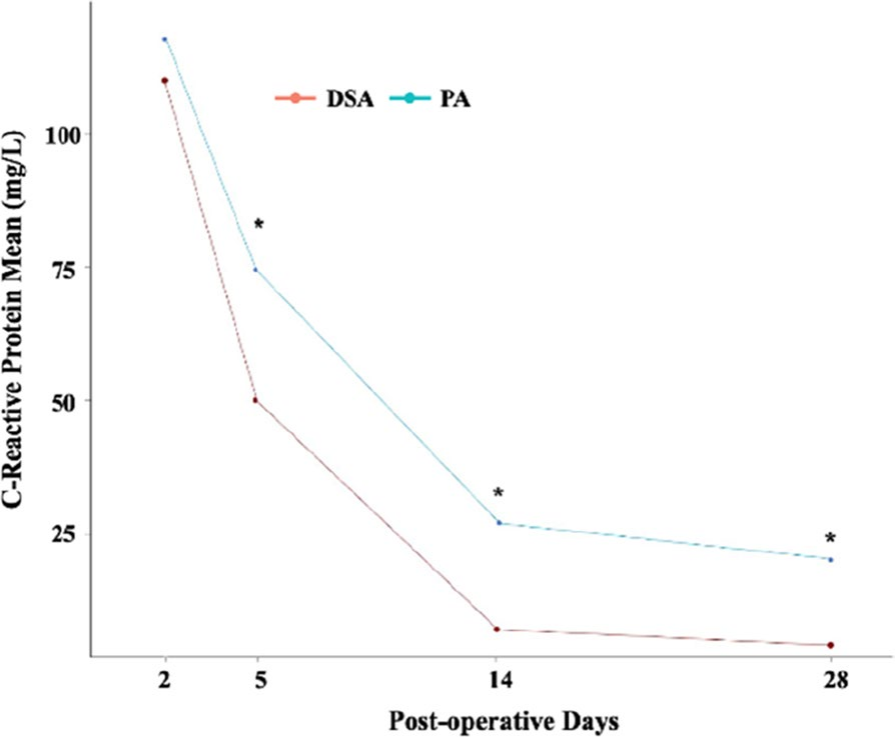
See text for description

## O16 Vitamin E-diffused highly cross-linked polyethylene in total hip arthroplasty: a single-arm, prospective study of minimum 5 years

### Seung-Hoon Baek^1^, Hyeong-chan Seo^1^, Suk-Kyoon Song^2^, Shin-Yoon Kim^1^

#### ^1^Department of Orthopedic Surgery, Kyungpook National University Hospital, Daegu, South Korea; ^2^Department of Orthopedic Surgery, Daegu Catholic University Medical Center, Daegu, South Korea

##### **Correspondence:** Hyeong-chan Seo (os22145os@gmail.com)

*Arthroplasty 2024*, **6(Suppl 1):**O16

**Background and Aims**

Vitamin E-diffused highly crossed-linked polyethylene (VEPE) has recently gained popularity in total hip arthroplasty (THA), but the mid-term result after THA using ceramic-VEPE articulation is limited. The objective of this prospective study was to evaluate the clinical and radiological outcomes of THA using VEPE.

**Methods**

Seventy-five cementless THAs using VEPE with a minimum follow-up duration of 5 years were included. There were 49 males and 26 females with an average age of 54.5 years and the mean follow-up duration was 8.5 ± 0.9 years. The most common reason for THA was the osteonecrosis of the femoral head (45 hips, 60%). All THAs were performed with RingLoc^®^ cup (Zimmer Biomet, Warsaw, IN, USA), Taperloc Microplasty^®^ stem (Zimmer Biomet, Warsaw, IN, USA), and Delta ceramic head. Clinical evaluation included a modified Harris Hip score (HHS) and radiographic analysis was performed regarding the inclination and anteversion angle of the acetabular component, component stability, osteolysis, radiolucent line (RLL), stress shielding, spot weld, and cortical hypertrophy. Complications included recurrent dislocation, periprosthetic femoral fracture (PFF), periprosthetic joint infection (PJI), and iliopsoas tendinitis (IPT).

**Results**

The HHS improved from 57.6 preoperatively to 93 postoperatively (*P* < 0.001). The mean inclination and anteversion angle of the cup were 46.1° and 19.2°, respectively. There were osteolysis in 2 (2.7%), recurrent dislocation in 2 (2.7%), PJI in 2, IPT in 1, but no PFF. Two hips (2.7%) were revised due to PJI and recurrent dislocation, respectively. RLL was demonstrated in 9 hips (12%) in the cup and 62 hips (82.7%) in the noncoated area of the stem. There was stress shielding in all hips, cortical hypertrophy in 3 (4%), but no loosening of any components.

**Conclusion**

THA using VEPE showed favorable mid-term outcomes without component loosening. Further longer-term study is necessary to determine the in-vivo usefulness of VEPE in THA.

**Table 1 (Abstract O16):** Mid-term results of cementless total hip arthroplasty using Vitamin E-diffused highly crossed-linked polyethylene

Author	VEPE	Head	Hips	Age	FU *yr*	Size	Wear rate *mm/yr*	Osteolysis
Current Study	Diffused	Ceramic	75	55	8	28/36	0.058 (28 mm), 0.063 (36 mm)	2.7%
Nebergal et al.	Diffused	CoCr	51	59	3.2	32	0.012	NA
Shareghi et al.	Diffused	CoCr	37	58	5	32	0.071	NA
Lindalen et al.	Diffused	Ceramic	50	57	6	32/36	0.02 (32 mm), 0.01 (36 mm)	NA
Galea et al.	Diffused	CoCr	89	65	7	32/36	0.025	0
Busch et al.	Blended	Ceramic	400	62	5	32	0.024	NA
Massier et al.	Blended	Ceramic	199	65	6	28/32/36	0.028	0

## O17 Novel algorithm for gap balancing and bone cuts in robotic total knee arthroplasties significantly improves accuracy and surgical duration

### Zi Qiang Glen Liau^1^, Song Peng Matthew Ng^1^, Ryan Wai Keong Loke^2^

#### ^1^Department of Orthopaedic Surgery, National University Hospital, National University Health System, Singapore; ^2^Yong Loo Lin School of Medicine, National University of Singapore, Singapore

##### **Correspondence:** Zi Qiang Glen Liau (glen_liau@nuhs.edu.sg)

*Arthroplasty 2024*, **6(Suppl 1):**O17

**Background and Aims**

Robotic Total Knee Arthroplasties (rTKA) have become increasingly popular recently. Intra-operative manual planning of the positions of the femur and tibia implants in all degrees of freedom to achieve surgeons’ ideal targets and limits of bone cuts, gaps, and alignment is challenging. The final manually defined solution may not be optimal, and surgical duration extended. We aim to demonstrate the effectiveness of our novel algorithm clinically in terms of accuracy and surgical duration.

**Methods**

We developed a novel computational algorithm to achieve optimal positioning of rTKA implants in three-dimensional space. The initial set of parameters of the 3D positioning of implants in relation to each other and surgeon-defined target gaps and bone cuts are defined. The algorithm then determines various permutations that fulfill the targets with an accuracy of ±0.5 mm, while also ranking them according to a set of evidence-based criteria. We compared accuracy in achieving surgeon-defined target gaps between both groups, the intra-operative gap-balancing duration, and total surgical duration. Power analysis based on a pilot study showed that 44 patients were required.

**Results**

A prospective cohort study on 67 consecutive patients who underwent rTKA at a tertiary institution from November 2021 to December 2023 was performed. 25 patients (mean age 70.4 years ± 7.34) had our novel algorithm utilized intra-operatively, while 42 patients (mean age 70.5 years ± 6.90) did not. 92% of rTKAs using our algorithm achieved surgeon-defined target gaps ±1.5 mm, compared to 52% of rTKAs done manually (*P* = 0.003). The average difference between surgeon-defined target gaps and final achieved gaps was 1.08 ± 0.51 mm in the algorithm group, significantly lower than in the non-algorithm group −1.81 ± 1.04 mm (*P* = 0.003). Gap-balancing duration was significantly shorter: 1.16 min ± 0.11 with algorithm use, compared to 14.49 min ± 8.31 (*P *< 0.0001). The total surgical duration was also significantly lower with algorithm use, with a mean total surgical time of 38.4 min ± 14.94 compared to 73.66 min ± 19.61 (*P* = 0.0002) (Fig. 1).

**Conclusion**

Our novel algorithm for gap-balancing in rTKRs significantly improves the accuracy of achieving surgeons’ target extension and flexion gaps, along with gap-balancing and overall surgical duration. This is highly promising for achieving reproducibility and efficiency in rTKRs.

**Fig. 1 (Abstract O17):**
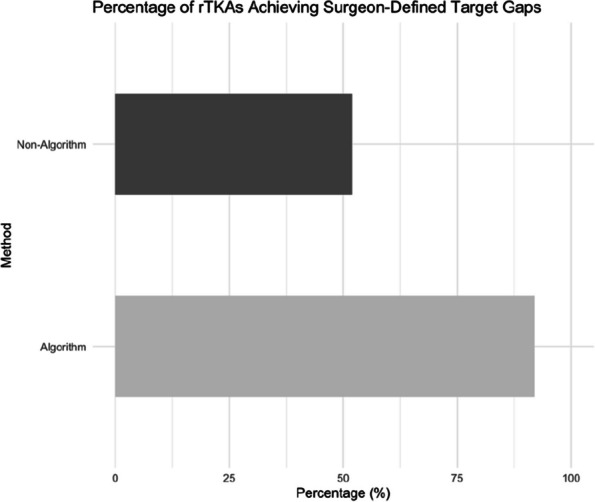
Proportion of rTKAs achieving surgeon-defined target gaps

## O18 Immersive virtual reality simulation training in total knee arthroplasty and transfer of skills to cadaver dissection: a pilot study

### Loh Kwong Weng^1^, Prevheenraj Naidu Thevaraj^1^, Azlina Amir Abbas^1^, Tan Sik Loo^1^, Wong Li Ping^2^, Khairul Anwar Ayob^1^

#### ^1^Joint Replacement Unit, Department of Orthopaedic Surgery, Faculty of Medicine, Kuala Lumpur, Malaysia; ^2^Department of Social and Preventive Medicine, Faculty of Medicine, Universiti Malaya, Kuala Lumpur, Malaysia

##### **Correspondence:** Loh Kwong Weng (melvinloh@um.edu.my)

*Arthroplasty 2024*, **6(Suppl 1):**O18

**Background and Aims**

Although the utilization of immersive virtual reality (iVR) for surgical training is widely recognized as a suitable alternative to traditional methods, the efficacy of iVR in technically demanding orthopedic procedures, such as total knee arthroplasty, remains underexplored. This study aims to evaluate whether immersive VR surpasses traditional technical surgical instructional videos in facilitating the acquisition and transfer of technical skills necessary for performing total knee arthroplasty (TKA) surgery [1]. 

**Methods**

Seven orthopedic surgery trainees were randomized into two groups to undergo surgical training for TKA using either a technical instructional video (CG) or a commercially available iVR training module (VR). The participants subsequently performed TKA on cadavers using standardized surgical instruments and implants. Subjective data were collected before, during, and after the procedure. Additionally, a blinded evaluator was employed to gather objective data during the cadaver dissection. 

**Results**

Seven postgraduate orthopedic surgery trainees from a single institution were randomized into two groups, with three trainees assigned to the CG group and four to the VR group. VR group scored significantly higher than the CG group in the flow of operation category of OSATS. Additionally, problem-based assessments of TKA showed that all VR group participants achieved a higher level of skill compared to the CG group participants. The questionnaire on attitudes towards iVR training revealed positive feedback from both groups, favoring iVR training for surgical education. The VR group completed the targeted steps of TKA faster and with a higher mean score for correct steps than the CR group, but neither difference was statistically significant (time: VR 46 m 30 s, CR 55 m 11 s, *P* = 0.26; score: VR 28.75, CR 20.67, *P* = 0.1) (Figs 1 and 2).

**Conclusion**

Although overall test scores and TKA completion times did not significantly differ between the iVR and control groups, the VR group excelled in specific skill assessments and problem-based evaluations. Positive trainee feedback suggests that iVR training is well-received and has the potential to enhance surgical education.

**Reference**

1. Mao RQ et al., Immersive Virtual Reality for Surgical Training: A Systematic Review. J Surg Res. 2021;268:40–58.

**Fig. 1 (Abstract O18):**
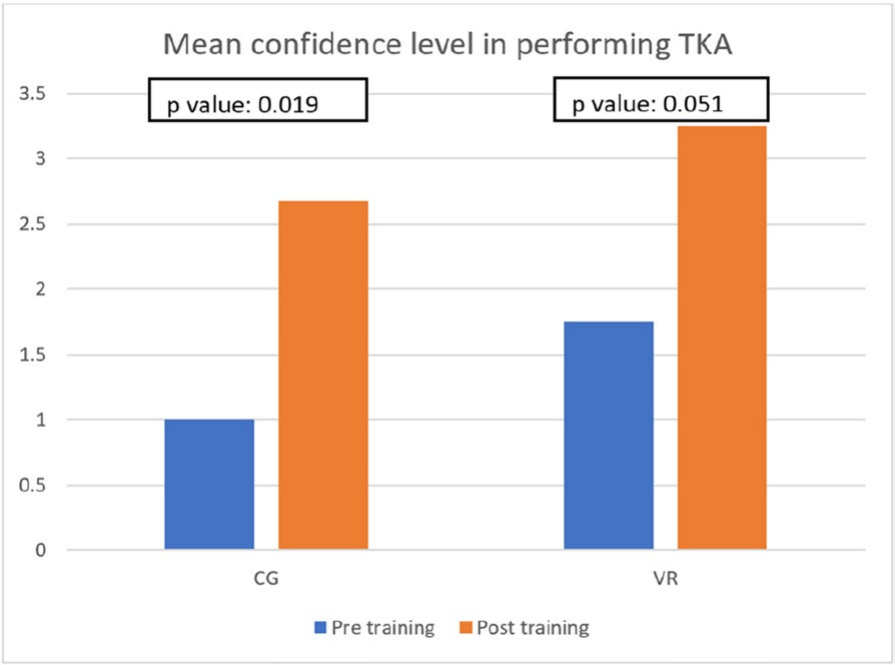
The mean confidence level of participants pre and post-training

**Fig. 2 (Abstract O18):**
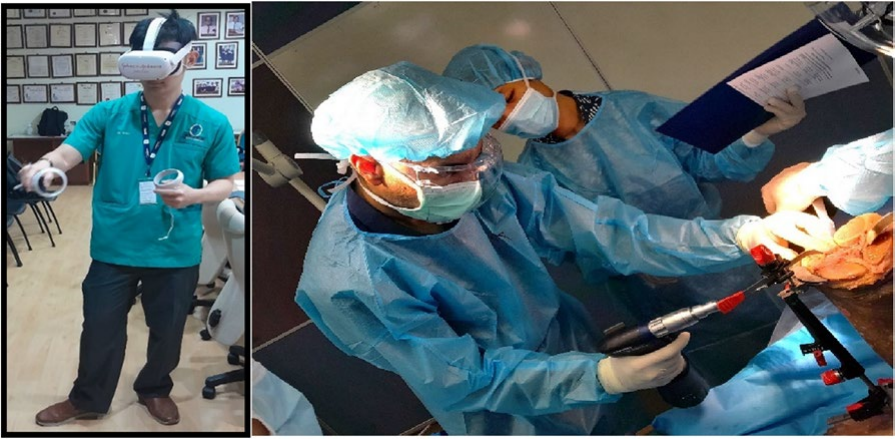
(Left) Participant in iVR training. (Right) Participant performing TKA on a cadaver under evaluation

## O19 Robot-assisted functional alignment total knee arthroplasty results in better early clinical outcomes: a retrospective cohort study

### Yinsing Sun^1^, Dehua Wang^1^, Mingfei Dong^1^, Lu Zhou^2^, Wei Huang^1^, Ke Li^1^

#### ^1^Department of Orthopedics, The First Affiliated Hospital of Chongqing Medical University, Chongqing, China; ^2^Department of Nursing, the First Affiliated Hospital of Chongqing Medical University, Chongqing, China

##### **Correspondence:** Yinsing Sun (18381657668@163.com)

*Arthroplasty 2024*, **6(Suppl 1):**O19

**Background and Aims**

To investigate the accuracy of joint prosthesis placement and early clinical outcomes and overall patient satisfaction with the procedure comparing robotic-assisted functional alignment total knee arthroplasty (RFA-TKA) with conventional manual mechanical alignment total knee arthroplasty (MA-TKA).

**Methods**

Data of patients who underwent TKA in the Department of Orthopaedics of The First Affiliated Hospital of Chongqing Medical University from June 2022 to December 2023, with a postoperative follow-up time of more than 3 months and complete follow-up data, were collected and analyzed retrospectively. The patients were divided into the RFA-TKA group and MA-TKA group according to the surgical procedures. The general information of the patients was recorded, the BMI of the patients was calculated, the overall satisfaction of the patients with the surgery was investigated, and the pre- and post-surgical HKA angle, mLDFA and mMPTA were measured and recorded, and the VAS scores, HSS scores, and WOMAC scores were assessed and recorded preoperatively and at the 3-week and 3-month postoperative follow-up. Comparisons of count data between groups were performed using independent samples t-tests, and the chi-square test was used for measurement data.

**Results**

A total of 210 patients were included in the study, including 105 in the RFA-TKA group and 105 in the MA-TKA group. The age differences (*t* = −1.360, *P* = 0.175) and BMI (*t* = −0.599, *P* = 0.550) between the two groups were not statistically significant. Between the two groups, preoperative HKA (*t* = −0.506, *P* = 0.614), mLDFA (*t* = −0.625, *P* = 0.533), mMPTA (*t* = −1.132, *P* = 0.259), VAS score (*t* = −0.073, *P* = 0.942), HSS score (*t* = 0.597, *P* = 0.551), WOMAC score (*t* =0.286, *P* = 0.775), postoperative HKA (*t* =0.002, *P* = 0.999), mLDFA (*t* =1.658, *P* = 0.099), mMPTA (*t* = 0.064, *P* = 0.949), and 3-month postoperative VAS scores (*t* = −0.959, *P* = 0.339) were not statistically significant. The differences between the two groups were statistically significant in 3-week postoperative VAS score (*t* = −16.094, *P* < 0.001), 3-week postoperative HSS score (*t* = 3.417, *P* = 0.001), 3-week postoperative WOMAC score (*t* = −6.794, *P* < 0.001), 3-month postoperative HSS score (*t* = 4.173, *P* < 0.001), 3-month postoperative WOMAC score (*t* = −5.494, *P* < 0.001), and overall patient satisfaction with surgery (*t* = 7.958, *P* = 0.005) were statistically significant [1–4].

**Conclusion**

RFA-TKA has better early clinical function score performance and higher patient satisfaction compared to MA-TKA.

**References**

1. Oussedik S, Abdel MP, Victor J, Pagnano MW, Haddad FS. Alignment in total knee arthroplasty. Bone Joint J. 2020 Mar;102-B(3):276–279.
2. Zheng H, Chen M, Yang D, Shao H, Zhou Y. Robotic-assisted differential total knee arthroplasty with patient-specific implants: surgical techniques and preliminary results. Arthroplasty. 2024 Jun 10;6(1):34
3. Chang JS, Kayani B, Wallace C, Haddad FS. Functional alignment achieves soft-tissue balance in total knee arthroplasty as measured with quantitative sensor-guided technology. Bone Joint J. 2021 Mar;103-B(3):507–514.
4. Agarwal N, To K, McDonnell S, Khan W. Clinical and Radiological Outcomes in Robotic-Assisted Total Knee Arthroplasty: A Systematic Review and Meta-Analysis. J Arthroplasty. 2020 Nov;35(11):3393–3409.e2.

## O20 Personalized 3D printing osteotomy guide plate assisted artificial total knee replacement for stiff knee

### Hao Chen^1^, Lin Guo^2^, Hongquan Heng^1^, Rui He^1^, Pengfei Yang^1^, Liu Yang^1^

#### ^1^Center for Joint Surgery of the First Affiliated Hospital of the PLA Army Medical University, Chongqing, China; ^2^Center for Orthopaedic Sports Medical of the First Affiliated Hospital of the PLA Army Medical University, Chongqing, China

##### **Correspondence:** Hao Chen (beyondest@126.com)

*Arthroplasty 2024*, **6(Suppl 1):**O20

**Background and Aims**

Artificial joint replacement surgery for stiff knees has considerable technical difficulty, and traditional release and exposure techniques are inefficient and prone to complications. The emerging CAD and 3D printing technologies provide new solutions. This study aims to observe the clinical efficacy and safety of using personalized 3D-printed osteotomy guide plates to assist in artificial joint replacement surgery for stiff knees [1].

**Methods**

From October 2022 to December 2023, our center treated 6 patients who underwent knee replacement surgery. All patients had straight knee joint stiffness before surgery and were assisted with personalized 3D-printed osteotomy guides for artificial joint replacement. Among them, there were 4 males and 2 females, aged 26 to 67 years, with an average age of 52 years. During the surgery, personalized osteotomy guides were used to assist in tibial and femoral condyle osteotomy, forming a straight gap and obtaining operating space to expose the surgical field. Compare knee joint range of motion, pain VAS score, and knee joint HSS score before and after surgery to assess knee joint function.

**Results**

All 6 cases were followed up for 6–9 months, with an average of 7.4 months. The HSS score of the knee joint during the last follow-up increased from 5–33 (15.54 ± 9.53) points before surgery to 55–93 (85.62 ± 7.06) points after surgery. The range of motion of the joint increased from 0° to 40° (13.85° ± 14.60°) before surgery to 80° to 115° (93.46° ± 10.49°) after surgery, and the difference between before and after surgery was statistically significant (*P* < 0.01). Postoperative X-ray measurement indicates satisfactory prosthesis position and force line correction. No complications such as infection, knee elongation, periprosthetic fractures, prosthesis loosening, or vascular and nerve damage occurred. One case of calf intermuscular vein thrombosis occurred, and the thrombus disappeared after anticoagulant treatment according to the guidelines (Figs. 1 and 2).

**Conclusion**

Personalized 3D printed osteotomy guide plate designed based on the patient’s stiffness state and bony structure can significantly optimize the surgical steps, improve surgical efficiency, enhance knee joint mobility, reduce the risks and complications of traditional replacement surgery, improve surgical efficacy, and be reliable and easy to operate in the artificial joint replacement surgery of straight stiff knees. It is worthy of clinical promotion and application.

**Reference**

1. Honoré Fell et al., Customized 3D cutting guides: what applications in orthopedic surgery? *Orthopedics*, 2023, 19(854):2344–2349

**Fig. 1 (Abstract O20):**
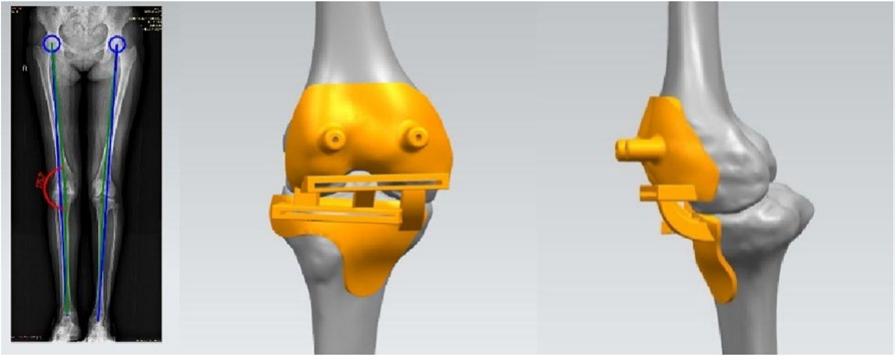
Design of 3D printed osteotomy guide plate

**Fig. 2 (Abstract O20):**
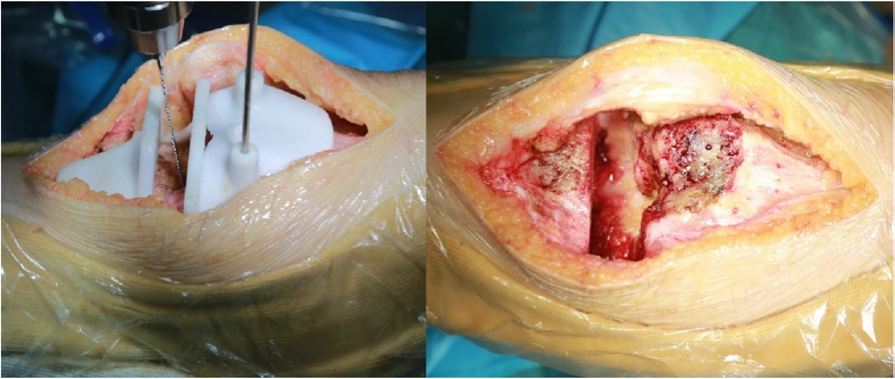
Application of 3D printed osteotomy guide plate during surgery

## O21 Midterm results of kinematic alignment technique in Oxford UKA

### Qidong Zhang, Wanshou Guo, Cheng Huang, Ran Ding, Weiguo Wang

#### Department of Orthopedics, China-Japan Friendship Hospital, Beijing, China

##### **Correspondence:** Qidong Zhang (18901078608@163.com)

*Arthroplasty 2024*, **6(Suppl 1):**O21

**Background and Aims**

Oxford unicompartmental knee arthroplasty (OUKA) requires good kinematics. OUKA bearing movement trajectory follows with femur, and poor trajectory causes complications, such as dislocation and pain. An accurate femoral position is crucial. We proposed an OUKA kinematic technique for femoral preparation based on tibial cut and overall alignment. This study aims to evaluate the midterm result of the novel technology [1].

**Methods**

The first 100 knees using the kinematic alignment technique were performed from June 2015 to January 2017. A tibial cut was performed first. A knee gap was inserted with a gap gauge to restore the native state. The femoral reference central line was drawn on the distal surface vertical to the tibial plane from the midpoint. In extension, the femoral drill reference (line A) was drawn on the anterior surface vertical to the tibial cut. Patients were followed up yearly thereafter. Clinical and radiological assessments were evaluated in terms of knee HSS score, VAS score, range of motion, and complications.

**Results**

Three cases were lost to follow-up and 3 died. The average follow-up was 8.6 ± 1.4 years. There was no revision, no dislocation, periprosthetic infection, or aseptic loosening. Three patients reported unexplained pain. One suffered progression of contralateral compartment arthritis The HSS score increased from preoperative 59.1 to 91.8. The ROM increased from preoperative 122.4° to 125.6°. The VAS score decreased from 6.9 to 1.6. 93% of prostheses were within the ideal range. The preoperative HKAA of weight-bearing X-ray was 173.3 ± 3.7°, and the postoperative HKAA was 177.2 ± 3.0°. The postoperative femoral varus/valgus angle was 2.0 ± 2.8°, and the femoral flexion/extension angle was 4.8 ± 3.4°; The varus/valgus angle of the postoperative tibial prosthesis is 1.0 ± 1.9°, and the posterior tilt angle of the tibia was 6.7 ± 2.3°. The distance between vertical prostheses and bearing lateral wall was 4.29 ± 2.46 mm (Figs 1–4).

**Conclusion**

The midterm result of the kinematic alignment OUKA technique is satisfactory, which conforms to the design principle of restoring the native knee. It is easy to perform with the advantage of no intramedullary interruption, and more rapid recovery. 

**Reference**

1. Zhang Q, et al. A novel extramedullary technique to guide femoral bone preparation in mobile unicompartmental knee arthroplasty based on tibial cut and overall alignment[J]. J Orthop Surg Res,2020,15(1):92.

**Fig. 1 (Abstract O21):**
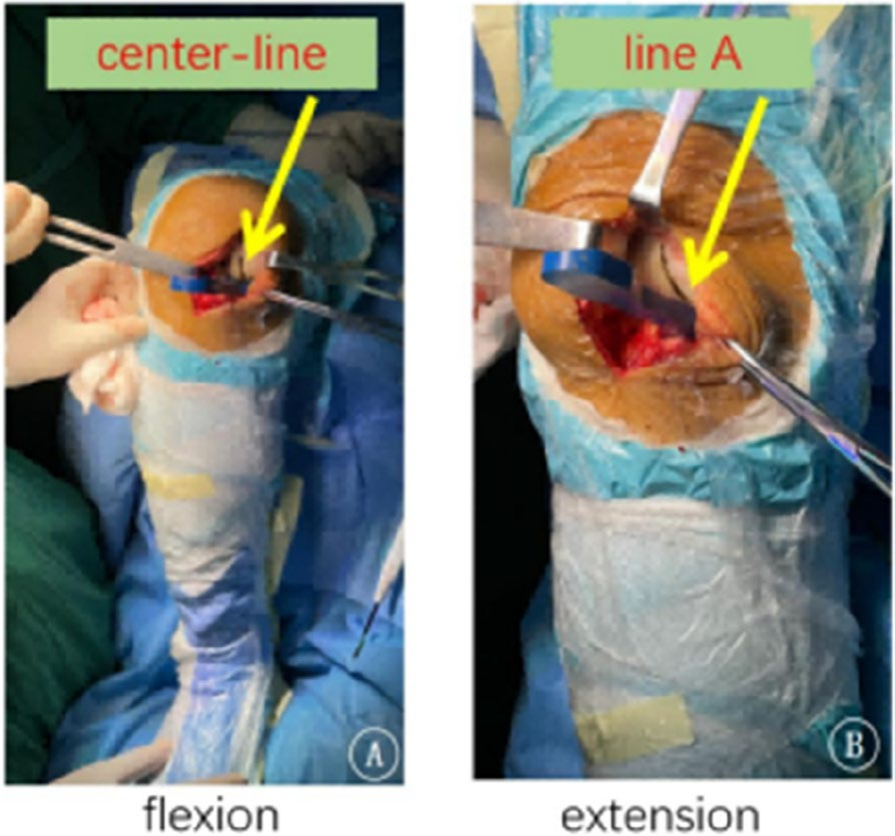
After the accurate tibial cut in flexion (**A**), the knee was brought into extension (**B**). **A** With an appropriate gap gauge (spacer block) in place, limb alignment was corrected to the native knee in flexion position The femoral reference central line was drawn on the distal femoral condyle articular surface which is vertical to the tibial cut plane from the midpoint of the tibial cut plane. **B** With an appropriate spacer block in place, limb alignment was corrected to neutral. The femoral drill reference (line A) was drawn on the anterior femoral surface vertical to the tibial cut plane

**Fig. 2 (Abstract O21):**
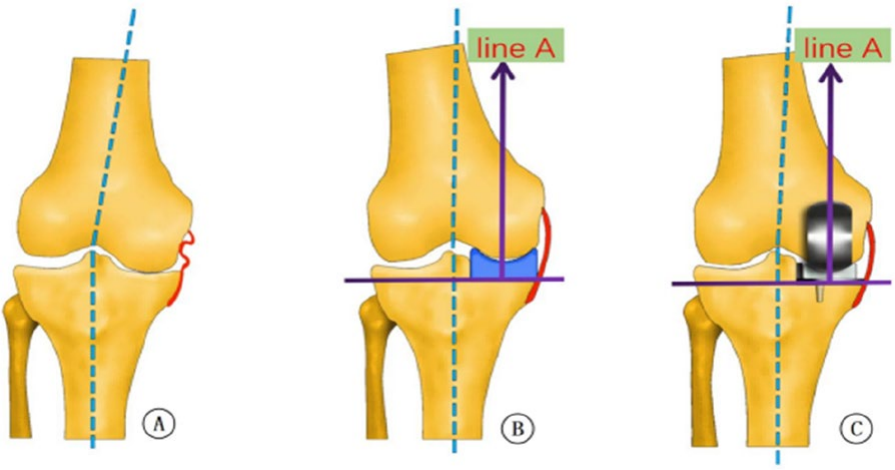
The kinematic alignment technique principle in OUKA. A Cartilage wear of the medial compartment causes the knee varus deformity; B Insert space block to fill fully the medial compartment and restore the tension of the medial collateral ligament, which keeps the knee to its native state before the disease. The femur is positioned with the reference of the tibia cut and the overall alignment. The femoral prosthesis is implanted with the direction of the mechanical axis in extension and central with the tibial implant. Therefore, the femur and tibia implants are compatible, allowing for natural movement recovery of the knee joint. The dashed line represents the overall lower limb mechanical alignment

**Fig. 3 (Abstract O21):**
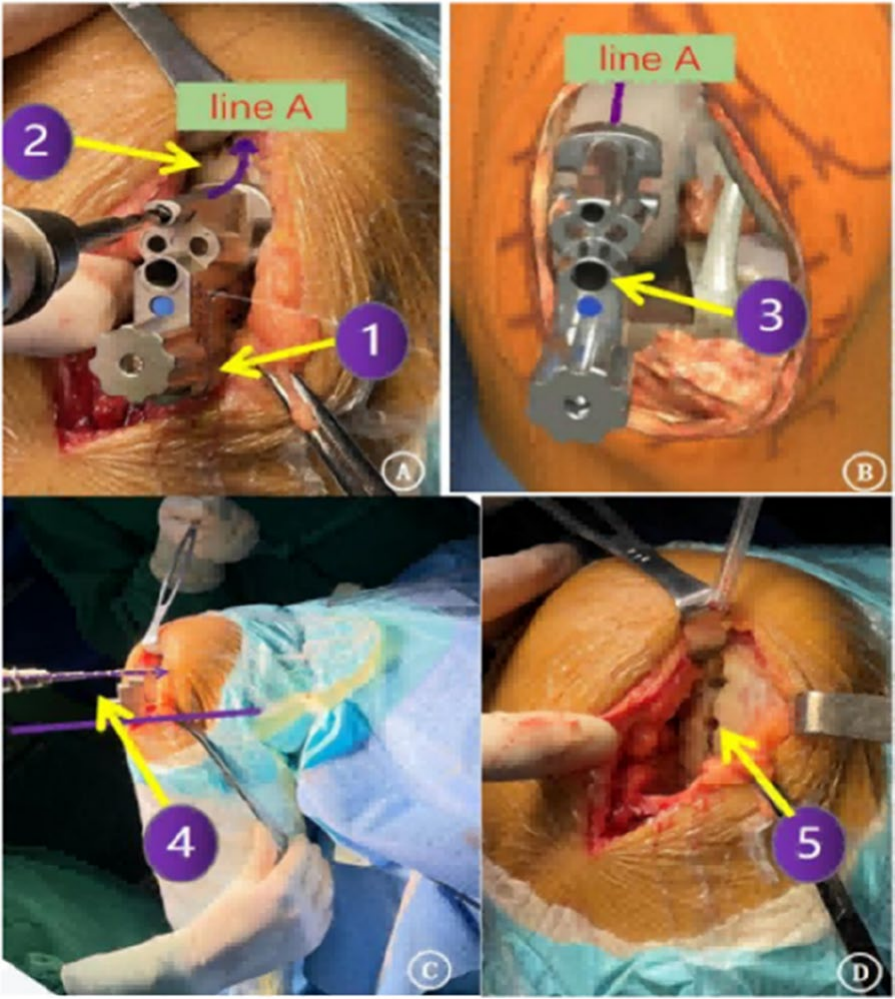
Key points for OUKA kinematic alignment technique: 1) Place the distal femoral drilling guide to the distal femur in the flexion position, the lower surface of the base is flat on the tibial cut plane, and the upper surface of the base is tightly attached to the cartilage surface of the posterior femoral condyle; 2) The drilling direction is parallel to line A; 3) Conform the drilling hole is located on the center-line of the distal femoral condyle through the guide window; 4) On the sagittal plane, the drill is parallel to the tibial cut surface through the drilling guide hole, which is slightly flexed along the anatomical axis of the femoral shaft; The drilling is located at the center-line of the distal articular surface of the femoral condyle

**Fig. 4 (Abstract O21):**
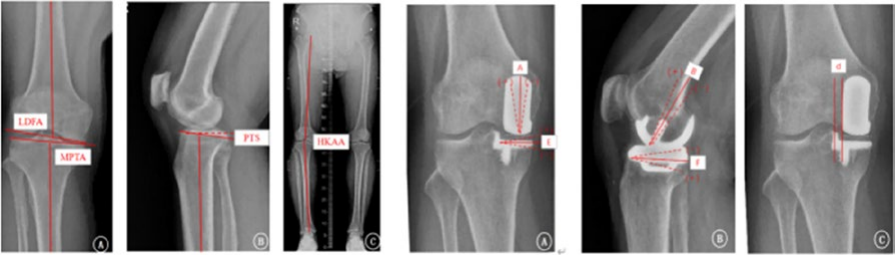
Measurement of radiological parameter

## O22 Comparison of clinical and radiological outcomes between tibia-based functionally and mechanically aligned total knee arthroplasty 

### Jong-Keun Seon, Hong-Yeol Yang

#### Department of Orthopedics, Chonnam National University Hospital, Gwangju, South Korea

##### **Correspondence:** Jong-Keun Seon (seonbell@jnu.ac.kr)

*Arthroplasty 2024*, **6(Suppl 1):**O22

**Background and Aims**

Functional alignment in total knee arthroplasty (TKA) has been introduced to restore the plane and obliquity of the native joint line and it places the balance of the soft tissues as the primary goal of alignment. The present study aimed to compare the clinical and radiological outcomes between patients who underwent conventional mechanically aligned total knee arthroplasty (TKA) and robotic arm-assisted TKA using functional alignment.

**Methods**

This prospective cohort study included 50 consecutive patients who underwent mechanically aligned TKA using conventional jigs followed by 50 consecutive patients who underwent tibia-based functionally aligned TKA. All the operative procedures were performed by a single surgeon using a medial parapatellar approach and identical implant designs. Using full-length standing hip-to-calcaneus radiographs, the hip-knee-ankle angle (HKA), joint line orientation angle (JLOA) in relation to the floor, and weight-bearing line (WBL) ratios of the traditional mechanical axis (MA) and ground mechanical axis (GA) passing through the knee joint were compared between the groups. 

**Results**

There were significant differences in the postoperative HKA angle due to a discrepancy in the targeted alignment strategy between the groups (functional, 2.0° versus mechanical 0.5°; *P* = 0.001). The postoperative JLOA in the functionally aligned group was more parallel in relation to the floor, while it was angled down toward the lateral side in the mechanically aligned group (0.6° versus −2.7°; *P* < 0.001). The GA in the functionally aligned group passed through a neutral position in the “true” condition when considering the calcaneus, while in the mechanically aligned group, the GA showed a lateral position (48.8% versus 53.8%; *P* = 0.001). Patients in the functionally aligned group had better postoperative clinical outcomes in terms of the Forgotten Joint Score and range of motion than those in the mechanically aligned group (all *P* < 0.05). 

**Conclusion**

Tibia-based functionally aligned TKA provided improved joint line parallelism to the floor with more neutral weight-bearing in the GA than mechanically aligned TKA. These differences suggest a more even load distribution across the knee and better clinical outcomes in the functionally aligned group. However, future studies are necessary to document its effect on implant survivorship.

## O23 Lifetime survival of octogenarians and factors affecting the survival following total knee arthroplasty

### Sang Jun Song, Cheol Hee Park, Young-Gook Kim

#### Department of Orthopaedic Surgery, College of Medicine, Kyung Hee University, Seoul, South Korea

##### **Correspondence:** Sang Jun Song (tesstore@empas.com)

*Arthroplasty 2024*, **6(Suppl 1):**O23

**Background and Aims**

The purpose is to analyze the clinical result, radiographic parameters, incidence of complications, and lifetime survival, and to investigate the factors affecting lifetime survival in octogenarians following total knee arthroplasty (TKA). 

**Methods**

We retrospectively reviewed 280 primary TKA procedures performed on octogenarians between 2000 and 2018. The average age was 82.0 years, and the age-adjusted Charlson comorbidity index was 4.4. Clinically, the WOMAC score and range of knee motion were evaluated. Radiographically, the hip-knee-ankle angle and component positions were measured. The incidence of postoperative complications, intensive care unit (ICU) admission, and readmission within postoperative 90 days were investigated. The lifetime survival (endpoint: death of patients), factors affecting survival, and the cutoff value for the affecting factors associated with death within 5 years postoperatively were analyzed.

**Results**

The clinical results improved, and radiographic parameters were appropriate at the final outpatient follow-up. Six patients (2.1%) experienced TKA-specific complications, including one wound dehiscence, one periprosthetic joint infection, one periprosthetic tibial fracture, and three deep vein thrombosis/pulmonary thromboembolism. Common nonspecific systemic complications included delirium (7.4%) and adverse urological events (5.7%). Overall, three cases (1.1%) were admitted to the ICU, and three (1.1%) were readmitted within 90 days postoperatively. The lifetime survival rates were 88.6% and 70.2% at postoperative 5- and 10 years, respectively. Age affected lifetime survival (Odds ratio 1.146, *P* = 0.037) (Fig. 1); the cutoff value for age-associated with 5-year mortality was 83 years. 

**Conclusion**

TKA is an acceptable treatment option for octogenarians, considering the clinical results, complication rates, and lifetime survival. However, this procedure should be performed carefully in octogenarians, especially those older than 83 years, which affects lifetime survival.

**Fig. 1 (Abstract O23):**
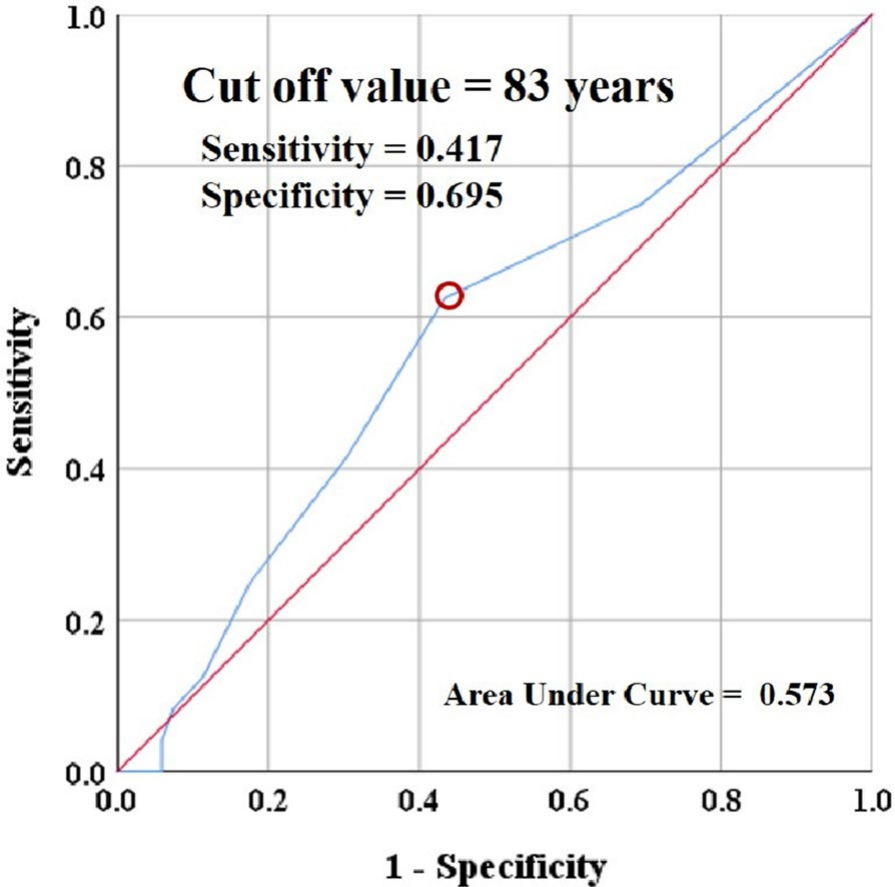
Cut-off value for age-associated with 5-year death

## O24 Evaluation of intraoperative soft tissue balance in journey II BCS with anatomically reconstructed articular surface geometry

### Maeda Takuma, Tomoyuki Matsumoto, Masanori Tsubosaka, Ryosuke Kuroda

#### Department of Orthopaedic Surgery, Kobe University Graduate School of Medicine, Kobe, Japan

##### **Correspondence:** Maeda Takuma (maetaku_ba68@yahoo.co.jp)

*Arthroplasty 2024*, **6(Suppl 1):**O24

**Background and Aims**

In recent years, implants that replicate the physiological articular surface shape have been used in total knee arthroplasty (TKA). However, intraoperative tensor measurements for these implants and the acceptable range of lateral laxity remain unclear. This study aimed to compare intraoperative soft tissue balance using the Journey II (Smith and Nephew) implant, which replicates the physiological articular surface shape, with conventional femoral trial components in the same patients [1].

**Methods**

The study included 30 knees undergoing primary TKA using the measured resection method. After osteotomy, the Journey II trial component was attached. The component gap (CG) and varus angle (VA) were measured using the OFR tensor at flexion angles of 0°, 10°, 30°, 45°, 60°, 90°, 120°, and 135°. These measurements were defined as Tensor Measurement 1 (TM1). The CG and VA measurements taken with a conventional trial component were defined as Tensor Measurement 2 (TM2). Differences were analyzed using the Wilcoxon rank sum test, with *P* < 0.05 indicating statistical significance.

**Results**

CG was significantly larger in TM1 than in TM2 at all flexion angles except 0° and 135°. VA was significantly larger in TM1 across all flexion angles (Figs. 1 and 2). 

**Conclusion**

Caution is required when using the Journey II implant for tensor measurements, as it may give the appearance of a larger VA, which could be mistakenly interpreted as increased lateral laxity compared to conventional components.

**Reference**

1. Grieco TF. et al., In Vivo Kinematic Comparison of a Bi-Cruciate Stabilized TKA and the Normal Knee Using Fluoroscopy. The Journal of Arthroplasty. 2017. 10.1016/j.arth.2017.09.035.

**Fig. 1 (Abstract O24):**
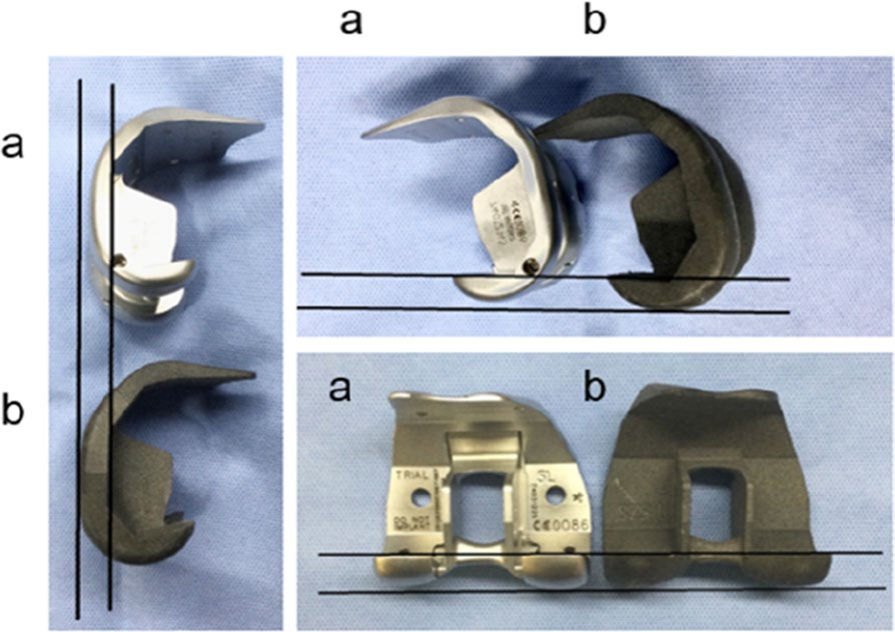
The shape of the Journey II femoral trial component (**a**) with a conventional trial component (**b**) from various angles

**Fig. 2 (Abstract O24):**
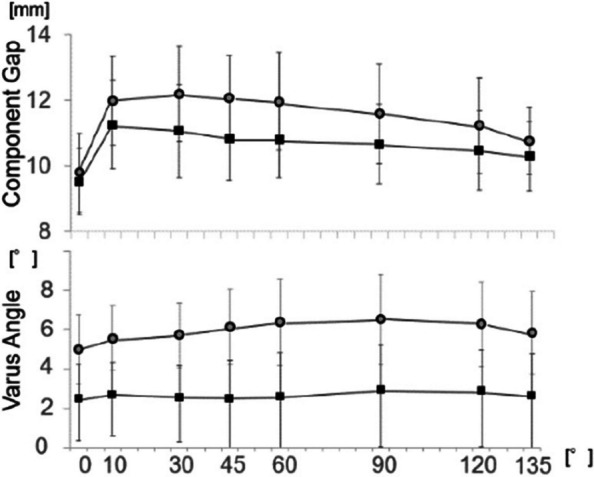
The component gap and varus angle at various flexion angles using the ORF tensor

## O25 Comparative study of postoperative outcomes in concurrent vs. sequential bilateral total knee arthroplasty: a retrospective review

### Vorawit Atipiboonsin, Atiwich Sangroungrai, Weerachai Kosuwon, Kamolsak Sukhonthanmarn

#### Khonkaen University, Khon Kaen, Thailand

##### **Correspondence:** Vorawit Atipiboonsin (billvorawit@gmail.com)

*Arthroplasty 2024*, **6(Suppl 1):**O25

**Background and Aims**

The safety and advantages of simultaneous bilateral total knee arthroplasty (SBLTKA) have been increasingly demonstrated in recent studies. Postoperative complications and mortality rates are lower than previously anticipated in selected patients. However, most studies focus on sequential SBLTKA (one team). Concurrent SBLTKA (two-team) remains less explored. This study aims to compare postoperative complications, readmission, and reoperation rates between concurrent SBLTKA (conSBLTKA) and sequential SBLTKA (seqSBLTKA) [1–3].

**Methods**

The retrospective review included patients who underwent SBLTKA at a single institution with at least 1 year of clinical follow-up between 2015 and 2023. From a total of consecutive reviews, 160 patients were eligible, comprising 103 conSBLTKA and 57 seqSBLTKA. Medical records were reviewed to collect demographic data, comorbidities, operative time, and postoperative events such as unplanned ICU admissions, blood transfusions, emergency department revisits, and readmissions for any cause.

**Results**

The overall mean age was 68.0 ± 7.7 years, and BMI was 26.7 ± 4.1. Operative time was significantly reduced in the conSBLTKA group at 138.2 ± 21.8 minutes compared to seqSBLTKA at 237.7 ± 33.7 minutes (*P* < 0.0001). The length of stay was shorter for conSBLTKA (5.5 ± 2.1 days) compared to seqSBLTKA (6.7 ± 3.0 days) (*P* = 0.004). Moreover, the total cost was significantly lower for conSBLTKA at 4400 ± 580 USD compared to seqSBLTKA at 5114 ± 1,382 USD (*P* < 0.001). Only one patient in the seqSBLTKA group required unplanned ICU admission. The readmission rate was 0.97% (*n* = 1) for conSBLTKA and 5.26% (*n* = 3) for seqSBLTKA (*P* = 0.096). Blood transfusion rates were 8.7% for conSBLTKA and 14.0% for seqSBLTKA (*P* = 0.298) (Figs 1 and 2).

**Conclusion**

Concurrent SBLTKA offers significant benefits for appropriately selected patients, including reduced operative time, shorter hospital stays, and lower costs, without increasing the risk of postoperative complications. Implementing a team-based approach and critical workflow is essential for success.

**References**

1. Warren JA, Siddiqi A, Krebs VE, Molloy R, Higuera CA, Piuzzi NS. Bilateral Simultaneous Total Knee Arthroplasty May Not Be Safe Even in the Healthiest Patients. Journal of Bone and Joint Surgery 2021;103:303-11. 10.2106/JBJS.20.01046.
2. Bullock DP, Sporer SM, Shirreffs TG. COMPARISON OF SIMULTANEOUS BILATERAL WITH UNILATERAL TOTAL KNEE ARTHROPLASTY IN TERMS OF PERIOPERATIVE COMPLICATIONS: The Journal of Bone and Joint Surgery-American Volume 2003;85:1981-6. 10.2106/00004623-200310000-00018.
3. Yong TM, Young EC, Molloy IB, Fisher BM, Keeney BJ, Moschetti WE. Long-Term Implant Survivorship and Modes of Failure in Simultaneous Concurrent Bilateral Total Knee Arthroplasty. The Journal of Arthroplasty 2020;35:139-44. 10.1016/j.arth.2019.08.011.

**Fig. 1 (Abstract O25):**
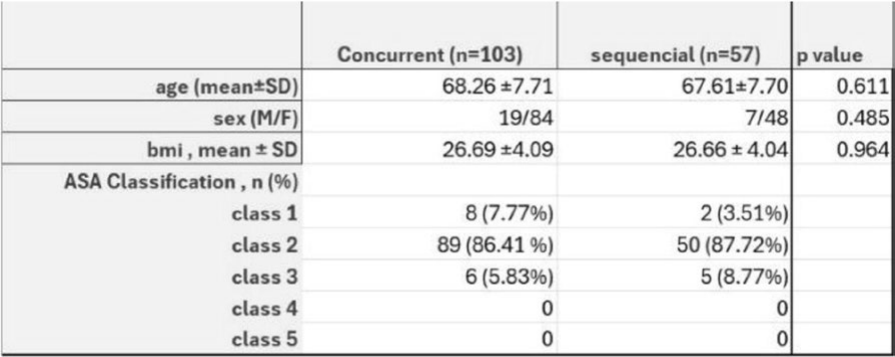
Demographic data

**Fig. 2 (Abstract O25):**
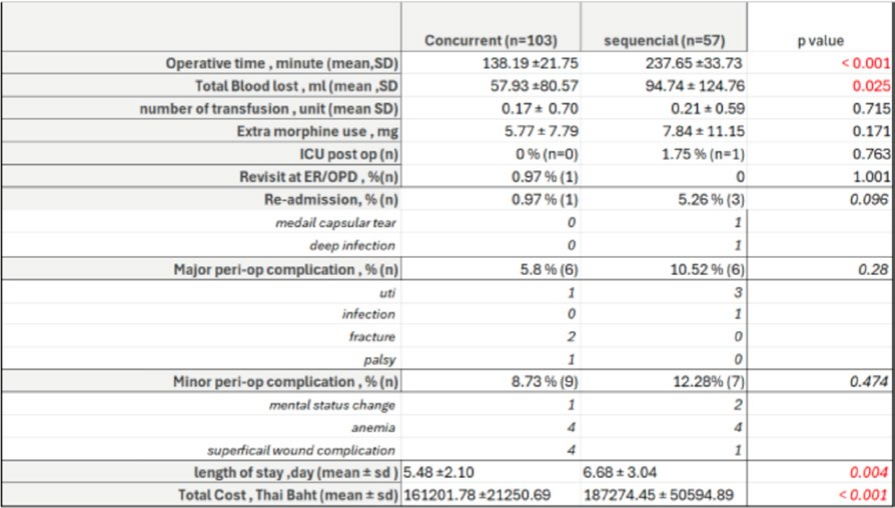
Postoperative outcome between conSBLTKA and seqSBLTKA

## O26 The estimated lifetime risk of revision after primary knee arthroplasty is influenced by implant design and patient age: data from National Joint Registries

### Nick Clement, Chloe Scott, Liam Yapp, Bin Chen

#### Orthopaedics Department, Royal Infirmary of Edinburgh, Little France, Edinburgh, UK

##### **Correspondence:** Bin Chen (chenbin@suda.edu.cn)

*Arthroplasty 2024*, **6(Suppl 1):**O26

**Background and Aims**

This study aimed to determine the lifetime risk of revision surgery after primary knee arthroplasty (KA) according to implant choice and patient age.

**Methods**

The risk of revision according to the implant type (unicondylar, unconstrained, semi, and fully constrained) was obtained from the National Joint Registry of England and Wales. Mortality risk according to age was estimated by the Scottish Arthroplasty Project (1998–2019). The cumulative incidence of revision and death was calculated up to twenty years. The lifetime risk was calculated as a percentage for patients aged 45–99 years using multiple decrement lifetable methodology according to the implant type.

**Results**

The lifetime risk of revision varied according to the implant, with unconstrained having the lowest risk and fully constrained and unicondylar having the highest risks, which increased with younger age. For an “average” patient aged between 65 and 69 years the revision risk for an unconstrained (3.61%, 95% confidence interval [CI] 3.35 to 3.90), semi-constrained (7.18%, 95%CI 3.90 to 13.60), unicondylar (13.67%, 95%CI 12.35 to 15.20) KA and fully constrained (15.42%, 95%CI 9.16 to 25.65) total KA gradually increased. Relative to an unconstrained KA the risk of revision for a semi-constrained implant was similar in patients aged <65 years (relative risk < 2), whereas when this was employed in those ≥65 years the relative risk was more than double (relative risk ≥ 2). Relative to an unconstrained KA the risk of revision for a fully constrained or a unicondylar KA was more than double in patients aged <55 years (relative risk > 2) and more than triple (relative risk ≥ 3) in patients ≥55 years (Fig. 1).

**Conclusion**

The estimated lifetime risk of revision following KA, when accounting for the competing risk of death, was dependent on patient age and implant type.

**Fig. 1 (Abstract O26):**
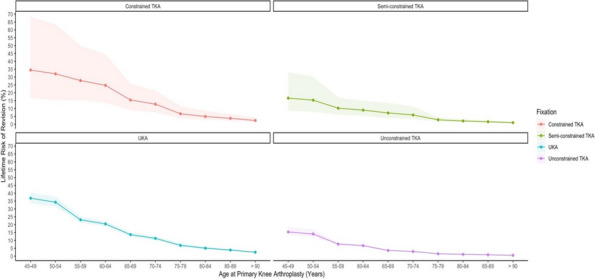
Lifetime risk of revision of knee according to implant and patient age at time of surgery

## O27 Perioperative body fluid retention following modern pain management protocol in TKA

### Pakpoom Somrak, Aree Tanavalee, Srihatach Ngarmukos, Chotetawan Tanavalee, Nonn Jaruthien, Wirinaree Khampitak

#### Department of Orthopaedics Surgery, Faculty of Medicine, Chulalongkorn University, Bangkok, Thailand

##### **Correspondence:** Pakpoom Somrak (pakpoomsomrak@gmail.com)

*Arthroplasty 2024*, **6(Suppl 1):**O27

**Background**

Perioperative fluid management in patients undergoing total knee arthroplasty (TKA) varies among the treatment protocols of orthopedic surgeons and anesthesiologists, depending on several factors [1]. With the use of multimodal pain control, some medications, especially corticosteroids and NSAIDs widely used in most protocols in arthroplasty surgeries, can precipitate sodium and water retention [2,3], which can compromise cardiopulmonary function, cause interstitial edema, and impair tissue reparation [4-7]. However, no study has focused on the quantitative change of body fluid measurements during this procedure. We inspected the perioperative total body water (TBW) change in patients undergoing TKA [8-20].

**Methods**

A consecutive series of 85 adult patients scheduled to be admitted in the evening underwent primary unilateral TKA in the morning of the following day with a single perioperative multimodal pain control and rehabilitation protocol. All patients underwent five consecutive multi-frequency bioelectrical impedance analysis (BIA) scans at baseline, postoperative day 1 (POD 1), postoperative day 3 (POD 3), two weeks, and six weeks. Changes in TBW, body weight, corticosteroid-fluid retention dose-response relationship, and perioperative complications were evaluated.

**Results**

Seventy patients had completed all five scans and the final follow-up. Female patients were dominant, with a mean age and BMI of 69.5 years and 29.94 kg/m^2^, respectively. There were no perioperative complications. At 24 hours, the mean total fluid input and output were 3695.14 mL and 1983.43 mL, respectively, with a total volume increase of 1711.71 mL and a mean accumulative dosage of dexamethasone of 15.14 mg. The mean TBW increased by 2.61 L on POD 1 and continued up to 3.2 L on POD 3, which gradually decreased at two weeks and reached the baseline level at six weeks postoperatively. Similarly, the mean body weight increased to 2.8 kg on POD 1 and 3.42 kg on POD 3 and returned to baseline at six weeks. The mixed-effect linear regression analysis of accumulative dexamethasone dosage and water retention dose-response relationship showed that the beta coefficient was 0.1 with *P*-value = 0.008 for fluid volume change following TKA on POD 1 (Fig. 1).

**Conclusion**

Current peri-operative fluid management and multimodal pain control in TKA caused fluid retention, which occurred from POD 1, declined at two weeks, and returned to the baseline level at six weeks. Medication, especially doses and amounts of corticosteroids, were related to fluid retention. Surgeons should be aware of and pay more attention to fluid balance in TKA patients.

**References**

1. Liu MM, Reidy AB, Saatee S, Collard CD. Perioperative steroid management: Approaches based on current evidence. Anesthesiology 2017;127:1:166-72
2. Akerberg D, Ansari D, Bergenfeldt M, Andersson R, Tingstedt B. Early postoperative fluid retention is a strong predictor for complications after pancreatoduodenectomy. HPB (Oxford) 2019;21:12:1784-9
3. Frishman WH. Effects of nonsteroidal anti-inflammatory drug therapy on blood pressure and peripheral edema. Am J Cardiol 2002;89:6A:18D-25D
4. Glatz T, Kulemann B, Marjanovic G, Bregenzer S, Makowiec F, Hoeppner J. Postoperative fluid overload is a risk factor for adverse surgical outcome in patients undergoing esophagectomy for esophageal cancer: A retrospective study in 335 patients. BMC Surg 2017;17:1:6
5. Voldby AW, Brandstrup B. Fluid therapy in the perioperative setting-a clinical review. J Intensive Care 2016;4:27.
6. Holte K, Kristensen BB, Valentiner L, Foss NB, Husted H, Kehlet H. Liberal versus restrictive fluid management in knee arthroplasty: A randomized, double-blind study. Anesth Analg 2007;105:2:465-74
7. Holte K, Klarskov B, Christensen DS, Lund C, Nielsen KG, Bie P et al. Liberal versus restrictive fluid administration to improve recovery after laparoscopic cholecystectomy: A randomized, double-blind study. Ann Surg 2004;240:5:892-9
8. Jennings JM, Mejia M, Williams MA, Johnson RM, Yang CC, Dennis DA. The james a. Rand young investigator's award: Traditional intravenous fluid vs. Oral fluid administration in primary total knee arthroplasty: A randomized trial. J Arthroplasty 2020;35:6S:S3-S9
9. Srinivasa S, Taylor MH, Singh PP, Yu TC, Soop M, Hill AG. Randomized clinical trial of goal-directed fluid therapy within an enhanced recovery protocol for elective colectomy. Br J Surg 2013;100:1:66–74
10. Cecconi M, Fasano N, Langiano N, Divella M, Costa MG, Rhodes A et al. Goal-directed haemodynamic therapy during elective total hip arthroplasty under regional anaesthesia. Crit Care 2011;15:3:R132
11. Ward LC. Bioelectrical impedance analysis for body composition assessment: Reflections on accuracy, clinical utility, and standardisation. Eur J Clin Nutr 2019;73:2:194-9
12. Mulasi U, Kuchnia AJ, Cole AJ, Earthman CP. Bioimpedance at the bedside: Current applications, limitations, and opportunities. Nutr Clin Pract 2015;30:2:180-93
13. Fischer M, Matsuo K, Gonen M, Grant F, Dematteo RP, D'Angelica MI et al. Relationship between intraoperative fluid administration and perioperative outcome after pancreaticoduodenectomy: Results of a prospective randomized trial of acute normovolemic hemodilution compared with standard intraoperative management. Ann Surg 2010;252:6:952-8
14. Kataoka H. A new monitoring method for the estimation of body fluid status by digital weight scale incorporating bioelectrical impedance analyzer in definite heart failure patients. J Card Fail 2009;15:5:410-8
15. Marinier MC, Ogunsola AS, Elkins JM. Body composition changes in the immediate peri-operative period following total joint arthroplasty. J Electr Bioimpedance 2022;13:1:39–44
16. Ledford CK, Ruberte Thiele RA, Appleton JS, Jr., Butler RJ, Wellman SS, Attarian DE et al. Percent body fat more associated with perioperative risks after total joint arthroplasty than body mass index. J Arthroplasty 2014;29:9 Suppl:150-4
17. Pichonnaz C, Bassin JP, Currat D, Martin E, Jolles BM. Bioimpedance for oedema evaluation after total knee arthroplasty. Physiother Res Int 2013;18:3:140-7
18. Kertkiatkachorn W, Kampitak W, Tanavalee A, Ngarmukos S. Adductor canal block combined with ipack (interspace between the popliteal artery and the capsule of the posterior knee) block vs periarticular injection for analgesia after total knee arthroplasty: A randomized noninferiority trial. J Arthroplasty 2021;36:1:122-9 e1
19. Gibon E, Mouton A, Passeron D, Le Strat V, Graff W, Marmor S. Doctor, what does my knee arthroplasty weigh? J Arthroplasty 2014;29:11:2091-4
20. Loyd BJ, Burrows K, Forster JE, Stackhouse SK, Hogan C, Stevens-Lapsley JE. Reliability and precision of single frequency bioelectrical impedance assessment of lower extremity swelling following total knee arthroplasty. Physiother Theory Pract 2021;37:1:197–203

**Fig. 1 (Abstract O27):**
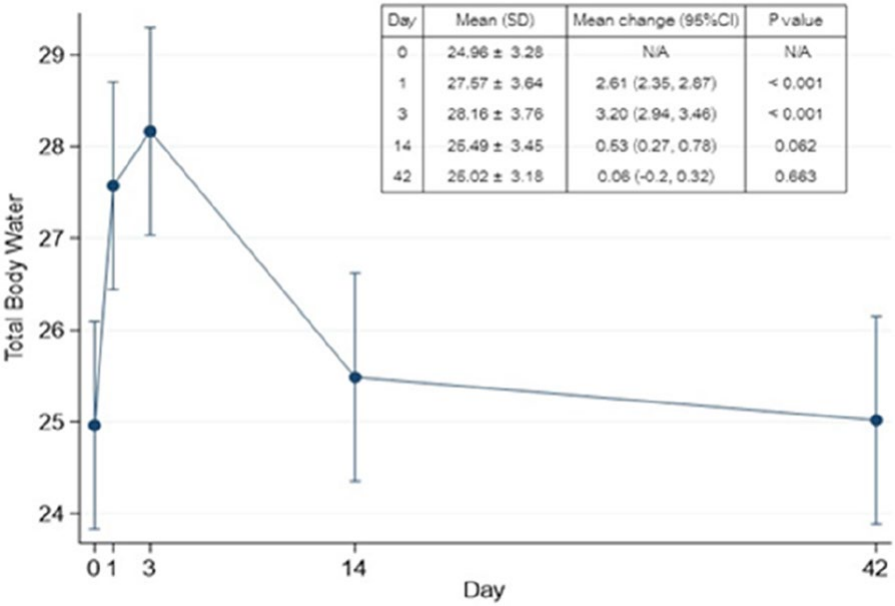
The mean ± standard deviation (SD) with a 95% confidence interval (CI) of total body water (TBW) showed significant changes from the baseline level (POD 0) on POD 1 and POD 3. It slowly decreased to the baseline level on POD 42

## O28 Repeated knee flexion maneuver after arthrotomy closure improved early active knee flexion in one-stage bilateral total knee arthroplasty: a double-blinded randomized controlled trial

### Chatchapol Ongkosit^1^, Noraworn Jirattikanwong^2^, Phichayut Phinyo^2^

#### ^1^Division of Orthopaedics, Nongbua Lamphu Hospital, Nongbua Lamphu, Thailand; ^2^Department of Family Medicine Research Areas Clinical Epidemiology, Faculty of Medicine, Chiang Mai University, Chiang Mai, Thailand

##### **Correspondence:** Chatchapol Ongkosit (ongkosit.c@gmail.com)

*Arthroplasty 2024*, **6(Suppl 1):**O28

**Background and Aims**

Restoring knee joint range of total knee arthroplasty (TKA). The arthrotomy closure's position and method are key factors and need to be watertight to avoid leakage, which can lead to increased tension and soft tissue imbalance, even with the knee in a flexed position. We propose that repeated knee flexion maneuvers might help rebalance soft tissue tension in the sutured area after arthrotomy closure. This study aims to investigate the effect of this technique on active knee flexion after primary TKA.

**Methods**

This prospective, double-blinded, randomized controlled trial was conducted in 46 patients (92 knees) undergoing single-setting, bilateral TKA. We conducted a randomized trial to allocate the intervention side. The intervention consisted of 15 repetitions of deep knee flexion and full extension, while the other side did not receive the intervention as the control. All patients followed the same postoperative pain control and rehabilitation protocols. The primary outcome measurement was active knee flexion on the first three days, the 2nd, 6th, and 12th weeks, six months, and one year postoperatively. Secondary outcomes were the degree of extension lag (EL), and postoperative pain at 6, 12, 24, 48, 72, and 96 hours, and at 2 and 6 weeks after surgery (Table 1).

**Results**

Active knee flexion was significantly greater in the intervention group compared to the control group on postoperative days 1, 2, 3, and at the 2-week follow-up. (97.4 ± 12 vs 91.2 ± 12 on day 3; *P* < 0.001, 112.2 ± 1.6º vs. 107.0 ± 1.6º at 2 weeks; *P* < 0.001). No significant differences between the groups were observed up to the one-year follow-up. There was only a significant difference for postoperative extension lag on postoperative day 1 and for VAS on postoperative week 2. (1.9 ± 2.1 vs 2.5 ± 2.1; *P* < 0.035).

**Conclusion**

Repeated knee flexion maneuvers, requiring minimal time to perform, demonstrated a significant improvement in active knee flexion within the first two weeks following TKA, as well as a slight reduction in pain at postoperative week 2.

**Table 1 (Abstract O28):** Comparison of postoperative degree of knee flexion, VAS pain score

Outcome	Intervention (Mean ± SD)	Control (Mean ± SD)	Mean difference* (95% CI)	*P*-value
**Postoperative degree of knee flexion**				
**Day 1**	94.3 (12.0)	87.4 (12.0)	6.9 (3.8, 10.0)	<0.001**
**Day 2**	94.1 (12.0)	89.3 (12.0)	4.8 (1.7, 8.0)	0.003**
**Day 3**	97.4 (12.0)	91.2 (12.0)	6.3 (3.1, 9.4)	<0.001**
**Week 2**	112.3 (12.4)	106.9 (12.4)	5.4 (2.3, 8.6)	<0.001**
**Week 6**	94.3 (12.0)	87.4 (12.0)	6.9 (3.8, 10.0)	<0.001**
**Postoperative VAS pain scores**				
**36 hours**	3.8 (1.9)	4.2 (1.9)	−0.6 (−1.2, 0.0)	0.058
**2 weeks**	1.9 (2.1)	2.5 (2.1)	−0.6 (−1.2, 0.0)	0.035**

## O29 No difference of immediate outcome after total knee arthroplasty in octogenarians compared to sexagenarians and septuagenarians: a propensity-matched cohort study

### Vorasilp Cheeva-akrapan^1^, Srihatach Ngarmukos^2^, Aree Tanavalee^2^, Chavarin Amarase^1^, Chotetawan Tanavalee^1^, Nonn Jaruthien^1^

#### ^1^Department of Orthopaedics, King Chulalongkorn Memorial Hospital, The Thai Red Cross Society, Bangkok, Thailand; ^2^Department of Orthopaedics, Faculty of Medicine, Chulalongkorn University, Bangkok, Thailand

##### **Correspondence:** Vorasilp Cheeva-akrapan (vorasilp.c@gmail.com)

*Arthroplasty 2024*, **6(Suppl 1):**O29

**Background and Aims**

Advancements in healthcare and positive lifestyle practices have significantly increased life expectancy in the Asian population. These factors may influence outcomes in arthroplasty procedures for octogenarians. In this study, we primarily aimed to compare the incidence of early hospital discharge (within three days post-operation) after total knee arthroplasty (TKA) between octogenarians and their sexagenarian and septuagenarian counterparts. Additionally, we sought to identify factors contributing to early discharge within these age groups.

**Methods**

A total of 150 patients undergoing primary TKA between 2020 and 2023 were included; 75 octogenarians were matched with 75 sexagenarians and septuagenarians. Groups were matched by sex, body mass index (BMI), American Society of Anesthesiologists (ASA) classification, comorbidities, and smoking history. The incidence of successful early hospital discharge after TKA was compared between the two groups using non-inferiority testing. Factors contributing to early hospital discharge in each group were analyzed using effect measure modification.

**Results**

There was no statistically significant difference in the incidence of successful early hospital discharge after TKA in octogenarians compared to sexagenarians and septuagenarians (84.7% and 91.5%, respectively; *P*-value 0.129). Factors including BMI, ASA classification, pre-operative hemoglobin level, operative time, and intra-operative blood loss did not significantly affect the odds of early discharge in either cohort.

**Conclusion**

Octogenarians can achieve immediate outcomes after TKA that are comparable to those of sexagenarians and septuagenarians. This finding supports providing surgical options to the increasing elderly population using existing resources. Standard and reproducible treatment protocols for TKA can be used to optimize patient outcomes in octogenarians.

## O30 Accuracy of surgeon-derived versus CT-based bony landmark registration during robotic-assisted TKA: a comparative study

### Rajeev Reddy Kikkuri

#### Sunshine Bone and Joint Institute Hyderabad, Hyderabad, India

*Arthroplasty 2024*, **6(Suppl 1):**O30

**Background**

Robot-assisted total knee arthroplasty (RA-TKA) aims to improve implant positioning accuracy and reduce outliers in achieving limb alignment. Concerns about potential errors during landmark registration in imageless system registration have mostly been studied on cadavers or saw-bone models. This is one of the first studies to compare the accuracy and reproducibility of bony landmark registration using CT-based and surgeon-derived landmarks during robotic-assisted TKA.

**Methods**

This study was a prospective observational analysis of 90 patients undergoing RA-TKA. After completion of bone registration with an error of less than 0.2 mm, the surgeon was then required to register pre-defined anatomical landmarks using the bone registration probe entirely relying on the tactile and visual feedback from the operating field. A Python script was then used to analyze and compare the corresponding landmarks between the surgeon-derived points and the CT data.

**Results**

84 patients with a mean age of 62.6 years (SD-8.9) were eligible for final analysis. Trans epicondylar axis (TEA) error with surgeon-derived points ranged from 7.9 degrees of internal rotation to 9.7 degrees of external rotation compared to CT-based clinical TEA. The mean 3-D distance error of the medial and lateral epicondyle was 6.1 mm (SD-3.1) and 5.4 mm (SD-2.3) respectively. Along the coronal plane, the majority of the surgeon-derived points were found to be over-cutting both the medial (N-72, 85.7%) and lateral femoral condyle (N-61, 72.6%); and also, the medial (N-60, 71.4%) and lateral posterior femoral condyle (N-53, 63%). Tibial resection error ranged from 2.4 mm proximally to 3.6 mm distally in the medial plateau and 3.8 mm proximally to 6.4 mm distally along the lateral tibial plateau (Fig. 1).

**Conclusion**

Surgeon-derived bony landmarks showed a wide variability when compared to 3D CT image-based landmarks, which may need to be further evaluated in larger and systematic cohorts to reduce the chances of potential risk of component malalignment in image-less RA-TKA.

**Fig. 1 (Abstract O30):**
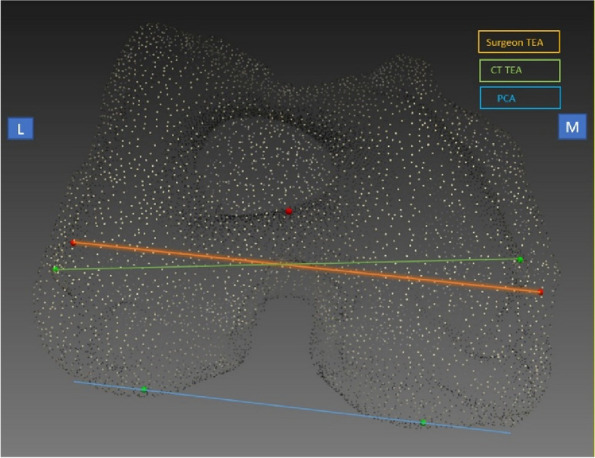
Novel software-generated post-surgical 3D model showing surgeon-derived versus Ct-derived TEA and PCA

## O31 Polypropylene mesh used as sheet provides decent reconstruction of quadriceps muscle tear in revision TKR setting

### Gahlot Nitesh

#### Department of Orthopaedic, All India Institute of Medical Sciences (AIIMS), Jodhpur, India

*Arthroplasty 2024*, **6(Suppl 1):**O31

**Background and Aims**

Literature for quadriceps tendon repair describes a Mesh to be used by passing through tendon and tuberosity. In this case, we have used it as a sheet for uniform continuous repair of the tendon and joint capsule [1].

**Case description**

A 76-year-old female, a known case of rheumatoid arthritis and hypertension, presented to the OPD with complaints of pain in her right knee and difficulty in walking after a fall at home 3 months back. The patient had been walking with a knee immobilizer brace and support since then. She had undergone primary bilateral total knee arthroplasty in 2020. Diagnostic imaging revealed Implant loosening along with a quadriceps tendon tear, resulting in an inability to extend her knee. She Underwent Stage I revision TKA. Intraoperatively infection was present around the implant, hence it was replaced with antibiotic cement moulds. The quadriceps tendon was partially reduced to the patella upper border, it was repaired using nonabsorbable sutures. But the rest of the tendon had retracted and was not of good quality for repair. To cover the defect, we used a polypropylene mesh and sutured it as a sheet over the complete extensor mechanism from the quadriceps muscle proximally to the tibial tuberosity distally and to the remaining retinaculum on the sides. Second-stage revision surgery was done after 3 months when all the infection markers came back negative. Due to poor collateral support checked intraoperatively, a hinged knee was inserted. The quadriceps tendon had healed over the mesh forming a continuous fibrous sheath, thus enabling a complete closure of the capsule. The patient was started on full weight bearing with brace support postoperatively (Figs 1 and 2). The patient gave their informed written consent to publish their information in an Open access journal.

**Conclusion**

This case highlights that reconstruction of the extensor mechanism of the knee after a quadriceps tendon tear with synthetic mesh applied as the sheet is an affordable, user-friendly, and widely accessible method. It can address large defects effectively while minimizing the risks of disease transmission and graft lengthening, resulting in satisfactory outcomes. The mesh facilitated the formation of a continuous sheet of thick fibrous tissue which is continuous with the extensor mechanism. 

**Reference**

1. Abdel MP, Pagnano MW, Perry KI, Hanssen AD. Extensor Mechanism Reconstruction with Use of Marlex Mesh. JBJS Essent Surg Tech. 2019 Jun 26;9(2):e21.10.2106/JBJS.ST.18.00106. PMID: 31579539; PMCID: PMC6687488.

**Fig. 1 (Abstract O31):**
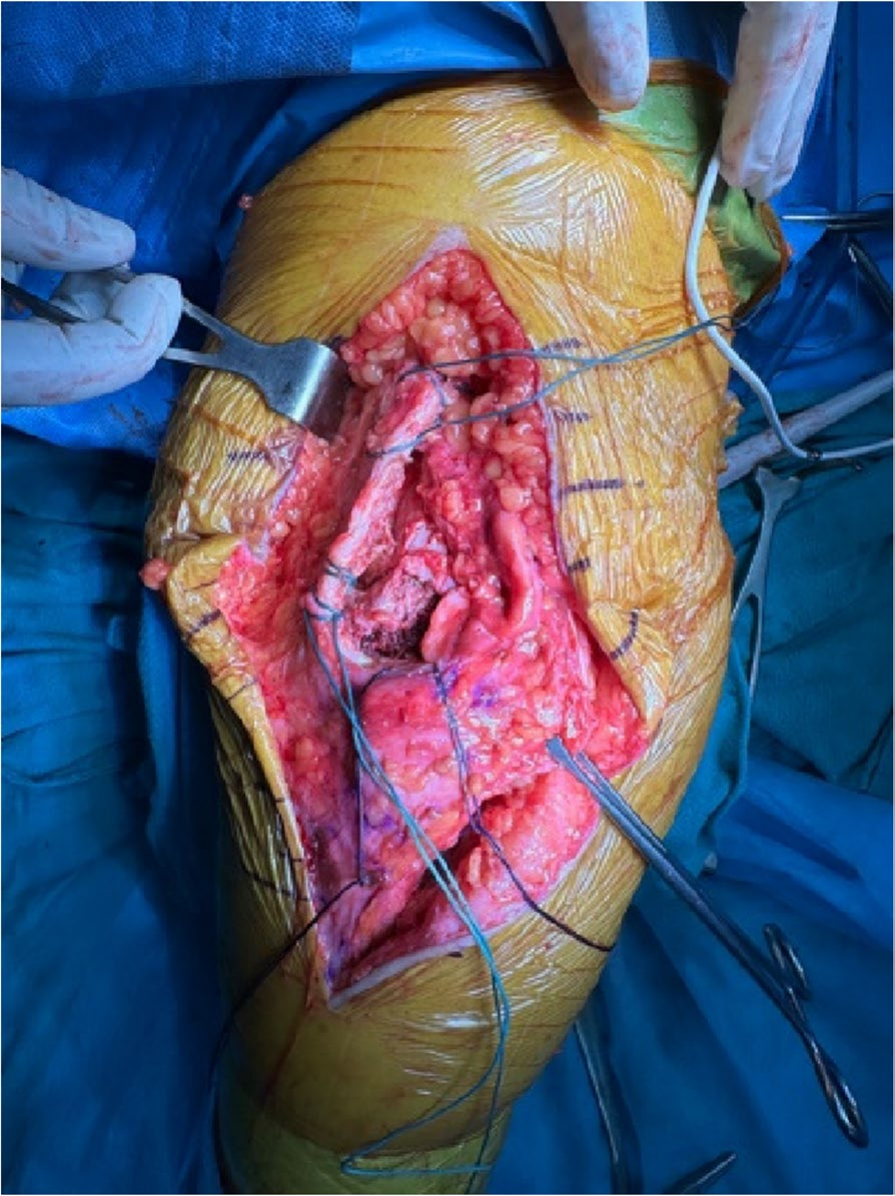
Extensor mechanism tear

**Fig. 2 (Abstract O31):**
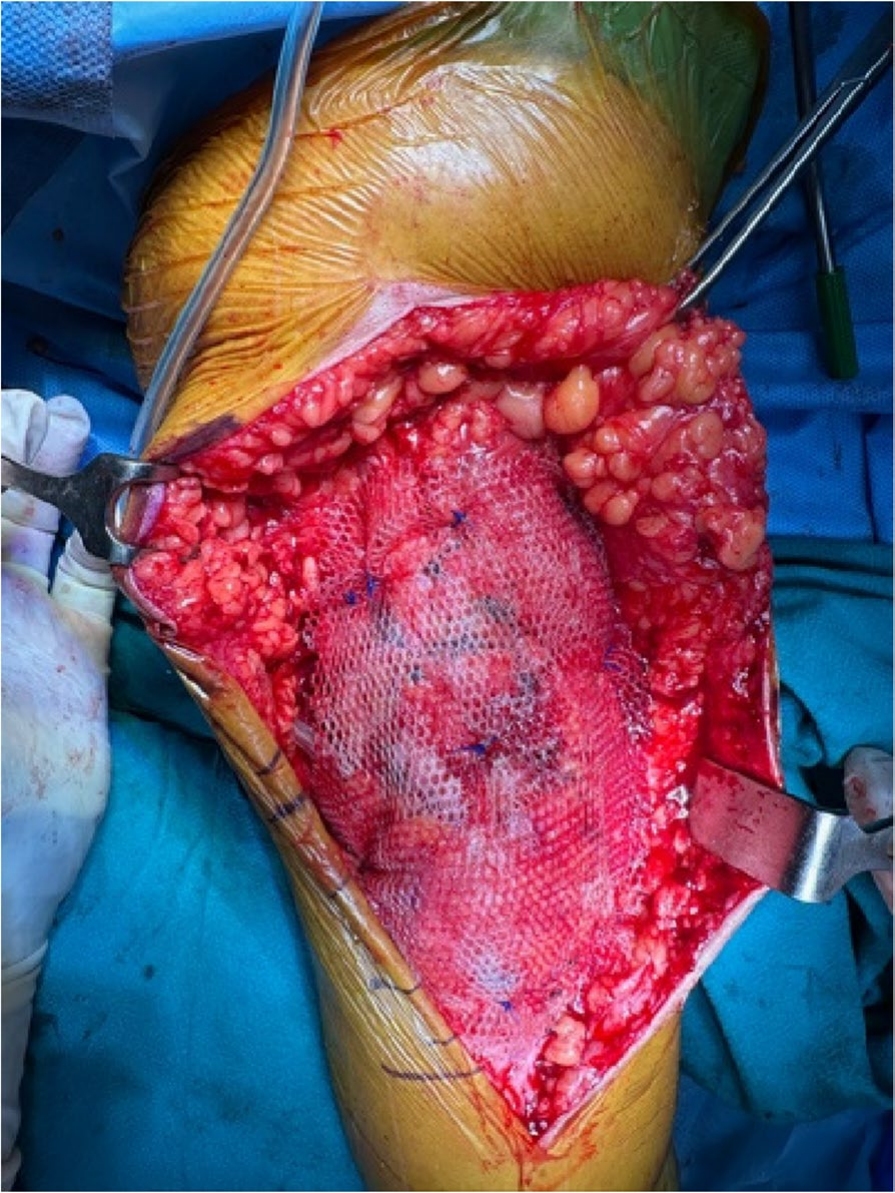
Repair using mesh as a sheet

## O32 Long-term results of short stem total hip arthroplasty in patients with osteonecrosis of the femoral head

### Bankchart Lajuntuk, Yingyong Suksathien

#### Department of Orthopedics, Maharat Nakhon Ratchasima Hospital, Nakhon Ratchasima, Thailand

##### **Correspondence:** Yingyong Suksathien (bankchartortho@gmail.com)

*Arthroplasty 2024*, **6(Suppl 1):**O32

**Introduction**

Short stem total hip arthroplasty (THA) is an alternative to conventional stem, designed to facilitate minimal-invasive surgery, physiological loading and preserve bone stock. However, there is insufficient evidence of the long-term outcomes in patients with osteonecrosis of the femoral head (ONFH). This study aims to analyze the clinical and radiographic results with a minimum follow-up of 10 years. Furthermore, the survival rate of the short stem was calculated.

**Methods**

There were 81 patients (101 hips) with a mean age of 45 years (range, 21–68) in this study. The Harris Hip Scores (HHS) and Forgotten Joint Scores (FJS) were recorded to evaluate the clinical results. The appearance of osteointegration and radiolucent line were reviewed. The mean follow-up period was 129.3 months (range, 120–154).

**Results**

At the latest follow-up, the mean HHS and FJS were 96.9 ± 5.7 (range, 69–100) and 95 ± 7 (range, 75–100) points respectively. The radiographic changes around the stem showed osteointegration mainly in the proximal part, zones 1 (94.1%), 2 (84.2%), 6 (99%), and 7 (92.1%). One stem underwent revision due to a periprosthetic fracture, using a conventional stem with a plate and screws. Survivorship of the stem with the endpoint for any reason was 99.01% (95% confidence interval [CI], 93.18%–99.86%), and for aseptic loosening was 100% at 10 years.

**Conclusion**

Short stems showed promising long-term clinical outcomes in patients with ONFH. The radiographic results demonstrated physiological proximal load transfer.

## O33 Early dislocation rate in a consecutive series of 1093 primary total hip arthroplasties using imageless navigation

### Sirawitz Khamphaeng, Yingyong Suksathien , Pattawat Chuvanichanon

#### Department of Orthopedics, Maharat Nakhon Ratchasima Hospital, Nakhon Ratchasima, Thailand

##### **Correspondence:** Yingyong Suksathien (sir_zeal@hotmail.com)

*Arthroplasty 2024*, **6(Suppl 1):**O33

**Background**

Postoperative hip dislocation remains a major complication in total hip arthroplasty. Various studies have demonstrated that several factors influence dislocation. While computer-assisted navigation has been proposed to enhance component alignment, its impact on dislocation rates remains unclear. This study aimed to investigate the early dislocation incidence and associated risk factors in 1093 primary THAs utilizing imageless navigation.

**Methods**

A retrospective review of patients undergoing imageless-navigated THA between February 2013 and December 2022 at Maharat Nakhon Ratchasima Hospital was conducted. Inclusion criteria comprised primary THA with a minimum 6-month follow-up. Imageless navigation guided acetabular cup placement, while femoral component positioning relied on surgeon expertise. Statistical analysis included univariate regression to identify dislocation risk factors.

**Results**

1093 THAs were analyzed. Dislocation occurred in 16 cases (1.5%), primarily non-traumatic and within 3.2 weeks postoperatively. Femoral neck fracture emerged as a significant risk factor (OR = 4.418, *P* = 0.004), while age, gender, femoral head size, and adherence to Lewinnek’s safe zone did not significantly affect dislocation rates.

**Conclusion**

Despite imageless navigation in THA, dislocation remains a concern. Femoral neck fractures significantly increase the risk of dislocation, emphasizing the importance of meticulous planning, surgical technique, post-operative care, and patient education in such cases.

## O34 Proximal femur biomechanics: a comparative study of prophylaxis wiring and non-wiring in hip arthroplasty

### Atiwich Sangroungrai, Rit Apinyankul, Kamolsak Sukhonthamarn, Vorawit Atipiboonsin, Thewarid Berkban, Surasith Piyasin, Teerawat Laonapakul, Weerachai Kosuwon

#### Department of Orthopedic, Faculty of Medicine, Khon Kaen University, Khon Kaen, Thailand

##### **Correspondence:** Atiwich Sangroungrai (yangsaan@gmail.com)

*Arthroplasty 2024*, **6(Suppl 1):**O34

**Background**

Intraoperative periprosthetic femur fracture presents a significant complication during total hip arthroplasty, demanding prompt recognition for optimal patient outcomes. This study aims to investigate proximal femur biomechanics and compare failure loads between prophylactic wiring and non-wiring techniques.

**Method**

Using Ansys, a finite element model was developed to assess biomechanical differences in the area and technique of wiring, identifying the strongest area and optimal wiring technique. Twenty fresh cadaveric femurs underwent standard preparation followed by cerclage wiring on the left femur. Biomechanical evaluations were conducted under axial loading on the femoral stem until catastrophic failure. Shapiro-Wilk test and independent *t*-tests were utilized for energy absorption, ultimate load, seeding load, and subsidence analyses.

**Result**

The wiring group exhibited higher absorption force before femoral stem failure (41.9 ± 18.1 Nm) compared to the non-wiring group (41.0 ± 19.1 Nm) (*P* = 0.918). However, ultimate load and seeding load were lower in the wiring group (7.7 ± 2.1 kN and 3.1 ± 0.7 kN, respectively) than in the non-wiring group (7.7 ± 2.0, *P* = 0.901), (3.4 ± 1.4, *P* = 0.589). Both groups demonstrated comparable subsidence distances: the wiring group (7.7 ± 2.6 mm) and the non-wiring group (7.7 ± 3.8 mm) (*P* = 0.978).

**Conclusion**

The wiring group exhibited higher energy absorption before fracture occurrence, but no significant difference in ultimate load to fracture was observed. Additionally, comparable subsidence distances suggest no substantial advantage of wiring for prophylaxis. It's noted that wiring may deform proximal morphology, potentially altering stem load distribution to the proximal femur.

## O35 Effects of knee center registration errors on reported alignment in robotic-assisted TKA

### Supitchakarn Cheewasukanon, Nonn Jaruthien, Chotetawan Tanavalee, Chavarin Amarase, Srihatach Ngarmukos, Aree Tanavalee 

#### Department of Orthopedic, Faculty of Medicine, Chulalongkorn University, Bangkok, Thailand 

##### **Correspondence:** Supitchakarn Cheewasukanon (supitchakarn.che@mfu.ac.th)

*Arthroplasty 2024*, **6(Suppl 1):**O35

**Introduction**

The global prevalence of knee osteoarthritis (OA) among individuals aged 40 and over is 22.9%, affecting approximately 654.1 million people. Total knee arthroplasty (TKA) is a proven treatment for end-stage knee OA, with 480,958 primary procedures performed in the USA in 2019. Robotic-assisted TKA, developed to enhance surgical precision, offers better accuracy and consistency compared to conventional TKA. However, errors in robotic-assisted TKA, particularly in registering anatomical landmarks, can affect surgical outcomes. This study investigates the impact of registration errors using the CORI Surgical System (Smith & Nephew, USA) on knee joint alignment. 

**Methods**

Conducted on four cadavers, deviations in anatomical landmark registration of 5 mm and 10 mm were introduced and analyzed. 

Results 

Results indicate that a 5 mm deviation in the femur led to changes of 1 ± 0.7 degrees in varus/valgus angulation and 0.9 ± 0.9 degrees in flexion/extension. A 10 mm deviation increased these changes to 2.4 ± 0.7 degrees and 2.2 ± 0.9 degrees, respectively. For the tibia, a 5 mm deviation caused changes of 0.8 ± 0.8 degrees in varus/valgus angulation and flexion/extension, increasing to 1.3 ± 0.7 degrees and 1.5 ± 0.9 degrees with a 10 mm deviation. 

**Conclusion**

These findings highlight the critical importance of precise anatomical mapping in robotic-assisted TKA to ensure optimal surgical outcomes and implant longevity. The study underscores the need for meticulous surgical techniques to minimize registration errors and their potential impact on knee joint alignment.

## O36 Comparative effect of mechanical and functional alignment in bilateral robotic total knee arthroplasty: a randomized controlled trial

### Utain Ketkaewsuwan, Patcharavit Ploynumpon, Thakrit Chompoosang

#### Hip & Knee Arthroplasty Unit, Department of Orthopedic, Rajavithi Hospital, Collage of Medicine, Rangsit University, Bangkok, Thailand

##### **Correspondence:** Utain Ketkaewsuwan (fluk.zhaoyun@gmail.com)

*Arthroplasty 2024*, **6(Suppl 1):**O36

**Background**

Functional alignment (FA) in total knee arthroplasty (TKA) can achieve soft tissue balance by fine-tunning adjustments of bony resections and component alignment with less soft tissue release. However, joint line orientation relative to the floor in the knee and ankle after TKA is not well studied. 

**Methods**

A randomized controlled trial was performed in 30 patients with robotic-assisted bilateral TKA using FA and mechanical alignment (MA) in the same patient. The outcome measures were as follows: (1) standing radiographic knee and ankle alignment; (2) clinical outcomes at 1, 3, and 6 months postoperatively (including forgotten joint score (FJS), KOOS, knee range of motion); (3) patient satisfaction score; and (4) soft tissue release.

**Results**

Postoperative hip-knee-ankle angles between the FA and MA groups were similar (2.4° versus 2.4°, *P* = 0.952). Knee joint line orientation was significantly more parallel to the floor in the FA group (3.0° versus 4.7°, *P* < 0.001). There was no significant difference in ankle joint line orientation relative to the floor in the FA and MA groups (91.0° versus 92.4°, *P* = 0.099 for tibial plafond inclination, and 92.5° versus 93.2°, *P* = 0.564 for talar dome inclination). However, in knees with preoperative varus with apex distal joint line orientation (coronal plane alignment of the knee (CPAK) classification type I), FA significantly achieved a more parallel knee and ankle joint line orientation relative to the floor (3.1° versus 5.1°, *P *= 0.002 for knee and 91.0° versus 93.5°, *P *= 0.028 for tibial plafond inclination). FA can obtain a balanced knee with significantly lower posteromedial releases (23.3% versus 76.7%, *P *< 0.001) with no superficial MCL release needed (0% versus 6.67%, *P *< 0.01). The FA group achieved significantly higher FJS at 3 months (53.3 versus 46.0, *p *= 0.015) and 6 months (67.8 versus 57.8, *P *< 0.001) with higher patient satisfaction scores (79.2 versus 84.3, *p *= 0.001).

**Conclusion**

Functional alignment can control the overall lower limb alignment similarly to mechanical alignment, with a knee joint line more parallel to the floor. Additionally, the ankle joint line was more parallel in the knees with CPAK type I. FA can also provide a more balanced knee with less soft tissue release, a higher functional score, and greater patient satisfaction compared to mechanical alignment.

## O37 The changing of flexion gap on quadricep snip and tibial tubercle osteotomy compare with medial parapatellar approach under computer axis TKA: a cadaveric study

### Pasit Wangsuekul, Chavanon Tsumanasrethakul, Kanik Saksupa, Puthi Tantikosol, Suttinont Surapuchong

#### Department of Orthopedics, Lerdsin Hospital, Bangkok, Thailand

##### **Correspondence:** Pasit Wangsuekul (pasitwangsuekul@gmail.com)

*Arthroplasty 2024*, **6(Suppl 1):**O37

**Background**

Quadricep snip (QS) and tibial tubercle osteotomy (TTO) approaches are used in revision total knee arthroplasty(rTKA). In our experience, some patients faced limited flexion after rTKA although equal gap measurement before the fixation of the tibial tubercle or re-approximate the quadricep. We persume that QS or TTO may alter the flexion gap in rTKA.

**Method**

The cadaveric study of 12 knees using computer axis TKA (CORI). All knees are done with a medial parapatellar approach (MP). The tension gap at all ranges of motion was measured after the component trial (Smith & Nephew, Legion) was inserted. 6 knees were done with TTO (TTO group) and others with QS (QS group) and measured the tension gap. We compare the different tension gaps between MP and TTO, MP and QS at 90 degrees of flexion.

**Result**

The mean flexion gap of MP in the TTO group is 0.55 mm and 0.46 mm in the QS group. The mean flexion gap compared with MP in the TTO group is 0.11 mm and the QS group is 1.04 mm (*P*-value = 0.001, 95%CI = 0.46 to 1.42). The mean different gap at 90–120 degrees in the TTO group is 0.11 mm and the QS group is 1.19 mm (*P*-value = 0.001, 95%CI = 0.53 to 1.64).

**Conclusion**

According to the cadaveric study, QS may alter the flexion gap by 1.04 mm. We suggest that before re-approximating the quadricep, the flexion gap may be +1 with the gap balance technique before re-approximating the quadricep in rTKA.

## O38 A comparative study of clinical outcomes between cruciate-retaining and posterior-stabilized in novel design gradually reducing radius total knee arthroplasty: a propensity score-matched cohort study

### Krittin Nimsirireungphol, Sakkadech Limmahakhun, Warakorn Jingjit, Kasisin Klunklin

#### Department of Orthopedic, Faculty of Medicine, Chiang Mai University, Chiang Mai, Thailand

##### **Correspondence:** Krittin Nimsirireungphol (krittin_fame@hotmail.com)

*Arthroplasty 2024*, **6(Suppl 1):**O38

**Background**

Total knee arthroplasty (TKA) is a widely utilized surgical intervention for end-stage knee osteoarthritis. The Cruciate-Retaining (CR) and Posterior-Stabilized (PS) techniques represent two primary approaches in TKA. Previous studies have shown no significant differences in clinical outcomes between these techniques in multi-radius designs. However, the novel gradually reducing femoral radius design in TKA aims to enhance knee kinematics and stability, potentially offering improved clinical outcomes.

**Objective**

This study aims to compare the clinical outcomes between the CR and PS techniques in a novel gradually reducing femoral radius design TKA among primary knee osteoarthritis patients.

**Methods**

A retrospective study was conducted including 176 primary knee osteoarthritis patients who underwent TKA (CR: 69 knees, PS: 107 knees) using the Attune prosthesis (Johnson & Johnson) by a single surgeon from January 2019 to December 2022. Patients with secondary knee osteoarthritis, extra-articular deformity, or septic arthritis were excluded. Clinical outcomes were assessed using the Knee Osteoarthritis Outcome Score (KOOS) questionnaire at preoperative, 3, 6, and 12-month postoperative intervals. Secondary outcomes included range of motion (ROM).

**Results**

Propensity score matching was utilized to ensure a balanced comparison. Functional outcomes, as measured by KOOS, indicated no significant differences between the CR and PS groups at 3, 6, and 12 months postoperatively. However, the CR group showed a statistically significant improvement in sports activity scores at the 12-month follow-up (*P* = 0.01). ROM outcomes also showed similar results between the two groups with no significant differences.

**Conclusion**

The novel gradually reducing femoral radius design in TKA provides comparable clinical outcomes for both CR and PS techniques. The CR technique demonstrated a significant advantage in sports activities at 12 months postoperatively, potentially due to more natural knee mechanics. These findings suggest that both techniques are effective, but patient lifestyle and activity goals should be considered when choosing the appropriate TKA approach. Further randomized controlled trials are warranted to confirm these results.

## O39 Comparison of outcomes between mechanical and kinematic plan in functionally aligned robotic-assisted total knee arthroplasty for severe varus osteoarthritis knee

### Rukthanin Ruktrakul, Rapeepat Narkbunnam, Chaturong Pornrattanamaneewong, Keerati Chareancholvanich

#### Department of Orthopaedic Surgery, Faculty of Medicine, Siriraj Hospital, Mahidol University, Bangkok, Thailand 

##### **Correspondence:** Rukthanin Ruktrakul (rruktrakul@gmail.com)

*Arthroplasty 2024*, **6(Suppl 1):**O39

**Purpose**

To compare outcomes after robotic-assisted total knee arthroplasty (MAKO) between using kinematic alignment and mechanical alignment in severe varus osteoarthritis knee.

**Material and methods**

A retrospective data was collected from 74 patients with severe varus osteoarthritic knee (mHKA varus ≥10°) who underwent unilateral Robotic-assisted TKA (MAKO) using functional alignment with the initial kinematic plan in comparison to the initial Mechanical plan (37 patients with MA plan, 37 patients with KA plan). Baseline characteristics, including age, BMI, ASA, implant design, and preoperative HKA angle were recorded. Post-operative alignment including HKA angle, joint line orientation angle, Femoral coronal alignment, Tibial coronal alignment, and Femoral rotation was recorded. Forgotten joint score, OKS, KOOS-PS, and EQ- 5D-5L were collected at 6 months postoperatively.

**Results**

Patients in the MA plan group had more varus alignment mHKA 13° (10.5°–15°) and KA plan 11° (10°–13°). Post-operative alignment including HKA angle, joint line orientation angle, Femoral coronal alignment, Tibial coronal alignment, and Femoral rotation was equivalent between groups. Patients reported outcomes including Forgotten joint score, OKS, KOOS-PS, and EQ-5D-5L were similar in both groups.

**Conclusion**

Severe varus osteoarthritis knee, TKA using functional alignment with kinematic alignment plan or mechanical alignment plan can provide similar outcomes.

## O40 The effect of joint line change on medial unicompartmental knee arthroplasty 

### Chanavee Jirapornkul, Nonn Jaruthien, Chotetawan Tanavalee, Chavarin Amarase, Srihatach Ngarmukos, Aree Tanavalee 

#### Department of Orthopedic, Faculty of Medicine, Chulalongkorn University, Bangkok, Thailand

##### **Correspondence:** Chanavee Jirapornkul (chanaveej@gmail.com)

*Arthroplasty 2024*, **6(Suppl 1):**O40

**Introduction**

Joint line change was a risk factor for unicompartmental knee arthroplasty (UKA) failures. There were many measurement methods for evaluating joint lines after medial UKA. We used a reliable and standardized measurement tool for the first time, the lateral cortex of the femur as a reference line to create an angle to compare the change in joint line. The study aimed to investigate the impact of change in joint line on outcomes and failure rates after medial UKA. 

**Methods**

This was a retrospective cohort study encompassed patients who underwent medial UKA between 2015 to 2019 at one institution. Radiological evaluation of the patients at one year after UKA included change in joint line and tibiofemoral angle. Categorize the study population into two cohorts: changes in joint lines measuring less than four millimeters and those with changes exceeding four millimeters. We calculated functional outcomes by Knee Society Knee Score (KSKS) and Knee Society Functional Score (KSFS) at one year after UKA. Survival rate was recorded at least five years after UKA.

**Results**

There were 206 patients who met the inclusion and exclusion criteria. There were 123 patients in a group joint line change of less than 4 mm and 83 patients in a group joint line change of more than 4 mm. Patient demographics data were not statistically significantly different between both groups. There were statistically significant differences between KSKS and KSFS between both groups. KSKS in group joint line change less than 4 mm was 83.86 ± 7.61 and KSKS in group joint line change more than 4 mm was 81.55 ± 7.06 (*P* = 0.029). KSFS in group joint line change less than 4 mm was 78.98 ± 7.86 and KSFS in group joint line change more than 4 mm was 73.49 ± 8.29 (*P* = 0.000). 9 patients had reoperation after UKA in 5 years postoperative period. The mean revision rate was 4.36%. There were no statistically significant different complications between both groups. 

**Discussion and Conclusion**

This study reported the mean prosthesis survival rate was 95.63%. The change in joint lines more than 4 mm affects the worse functional outcomes and should be avoided.

## O41 Cadaveric biomechanical analysis of multiple drilling on proximal tibia in total knee arthroplasty

### Vorawit Atipiboonsin, Kamolsak Sukhonthamarn, Weerachai Kosuwon, Rit Apinyankul, Atiwich Sangroungrai, Nataya Sritawan

#### Department of Orthopedics, Srinagarind Hospital, Khon Kaen University, Khon Kaen, Thailand 

##### **Correspondence:** Vorawit Atipiboonsin (vorawit.ati@gmail.com)

*Arthroplasty 2024*, **6(Suppl 1):**O41

**Introduction**

Multiple drilling in the proximal tibia is a standard procedure during total knee arthroplasty to enhance cement-bone interdigitation in a sclerotic medial tibial plateau. However, drilling may potentially weaken the proximal tibia leading to periprosthetic fracture or decrease prosthesis longevity. This study aimed to investigate the biomechanical strength after multiple drills the effect of different drill sizes and the number of drill holes [1-5].

**Methods**

The 3D model of the tibia was simulated for compression load using finite element analysis (FEA). Validation testing was conducted on 28 synthetic sawbones and 20 cadaveric tibia. Each test was allocated into 4 groups: control (no drill hole), group A (2 mm, 3 holes/cm^2^, group B (4 mm, 3 holes/cm^2^), and group C (2 mm, 8 holes/cm^2^) (Fig. 1). Multiple drills proceeded over the sclerotic bone of the medial tibial plateau with the same depth of 1 cm (Fig. 2). Compression axial load was applied at the medial tibial plateau until fracture or bone failure occurred (Fig. 3).

**Results**

Multiple drilling decreased the average ultimate strength of the tibia was observed in the FEA model, synthetic sawbone, and cadaveric testing across all groups. The control group has the highest ultimate strength (2672.0 ± 787.0 N), followed by group A (1765.5 ± 362.9 N), group C (1722.3 ± 505.6), and group B (1441.9 ± 693.1 N), respectively. The lowest strength was observed in group B (4 mm, 3 holes/cm^2^) compared to the control (*P* = 0.034). However, no significant difference was identified between drilling groups in the cadaveric study.

**Discussion and Conclusion**

Multiple drilling could weaken the tibial compression strength. Hole density might have less effect on bony strength than the pore size. Therefore, a smaller drill bit and proper pore density might be suggested to enhance bone-cement interdigitation on the sclerotic area for tibial implantation.

**References**

1. Cawley DT, Kelly N, McGarry JP, Shannon FJ. Cementing techniques for the tibial component in primary total knee replacement. The Bone & Joint Journal 2013; 95-B: 2955-300. 10.1302/0301-620X.95B3.29586.
2. Refsum AM, Nguyen UV, Gjertsen J-E, Espehaug B, Feenstad AM, Lein RK, et al. Cementing technique for primary knee arthroplasty: a scoping revievv. Acta Orthopaedica. 2019; 90: 582-9.10.1080/17453674.2019.1657333.
3. Van De Groes SAW, De Waal Malefijt MC, Verdonschot N. Influence of preparation techniques to the strength of the bone-cement interface behind theflange in total knee arthroplasty. *The Knee.* 2013; 20: 186-90. 10.1016/j.knee.20112.08.002
4. Damsgaard CW, Gad BV, Bono OJ, Anderson MC, Brown JM, Bono JV, et al. Intraoperative Proximal Tibia Periprosthetic Fractures in Primary Total Knee Arthroplasty. *I. Knee Surg.* 2021; 34: 1269-74. 10.1055/s-0040-1708037.
5. Ahn JH, Jeong SH, Lee SH. The effect of multiple drilling on a sclerotic proximal tibia during total knee arthroplasty. *International Orthopaedics (SICOT).* 2015; 339: 1077-83. 10.1007/s00264-014-2551-3.

**Fig. 1 (Abstract O41):**
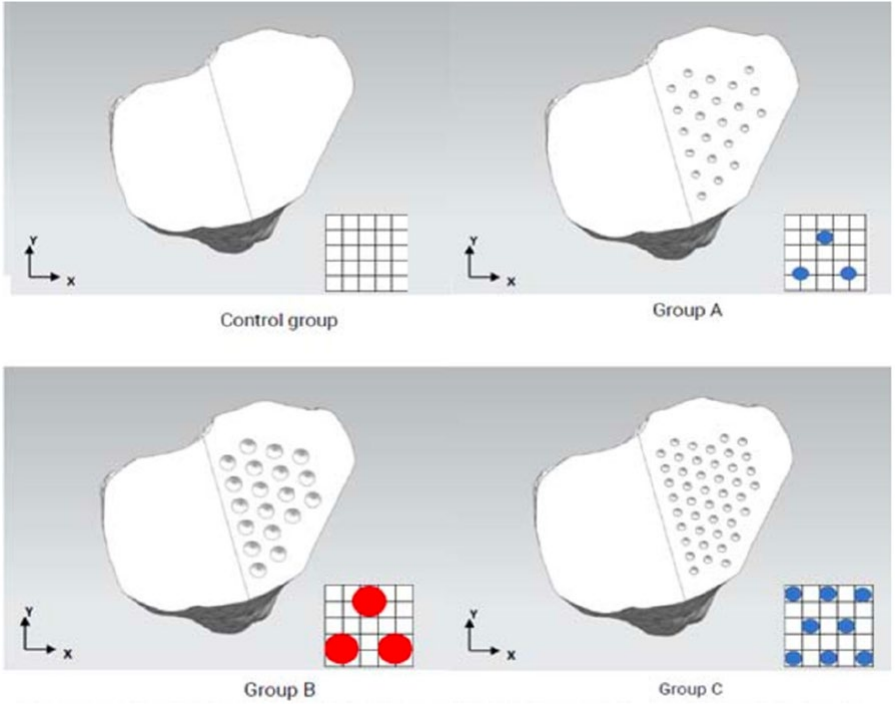
Illustrate of the drilling pattern and variation indensity on the medial tibial plateau for each group

**Fig. 2 (Abstract O41):**
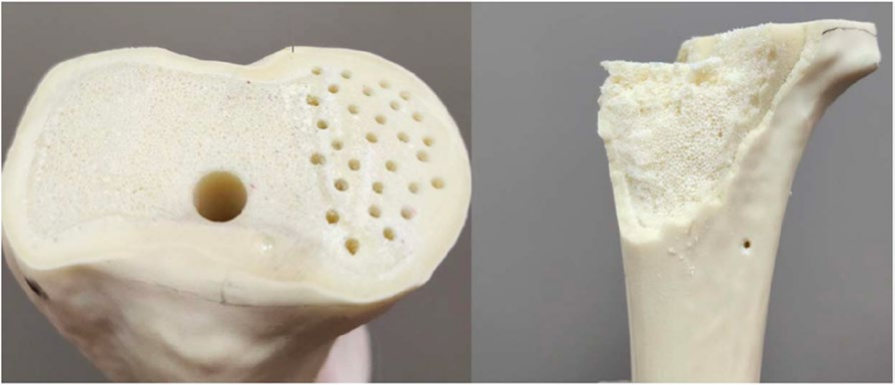
Demonstrate the drilling pattern from group A synthetic sawbone study before (left) and after (Right) compression load to failure

**Fig. 3 (Abstract O41):**
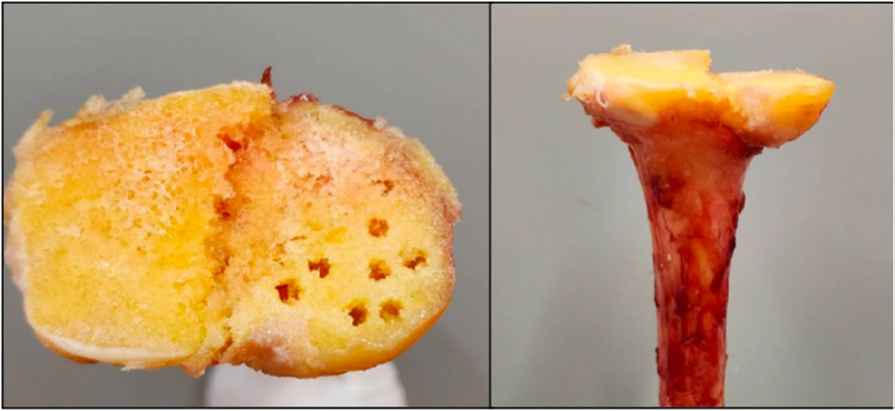
Show the pattern of failure that occur in right caadaveric tibia from group B in axial view (left) and coronal view (right)

## O42 Comparative analysis of cemented and cementless total knee arthroplasty implants in robotically assisted procedures

### Zhen Jonathan Liang^1^, Ryan Loke^1^, Xinyi Lim^2^, Glen Zi Qiang Liau^3^

#### ^1^NUS Yong Loo Lin School of Medicine, Singapore; ^2^Department of Orthopaedic Surgery, Alexandra Hospital, Singapore; ^3^Department of Orthopaedic Surgery, National University Hospital, Singapore

##### **Correspondence:** Zhen Jonathan Liang (liangzjonathan@gmail.com)

*Arthroplasty 2024*, **6(Suppl 1):**O42

**Background**

Total knee arthroplasty (TKA) is a widely performed surgical procedure for patients with severe knee arthritis. Recent advancements in technology have introduced robot-assisted techniques, offering potential benefits in terms of surgical outcomes. There has also been a revived interest in cementless fixation as a result of improvements in implant design and manufacturing technology^1^. This study aimed to compare the surgical time and postoperative haemoglobin levels between cemented and cementless TKA implants in robotically assisted procedures [1].

**Methods**

A retrospective analysis was conducted on patients who underwent cemented or cementless robot-assisted (ROSA^®^ Knee System) TKA between January 2022 and November 2023 performed by three different surgeons using Persona^®^ implants from Zimmer Biomet. Data on surgical time and postoperative haemoglobin levels were collected and analyzed using R Statistical Software.

**Results**

A total of 30 patients who underwent cemented and 26 who underwent cementless robotic TKA procedures were included in the analysis. The cementless group exhibited a statistically significant (*P* = 0.011) decrease in postoperative haemoglobin levels (Hb change = 2.15 ± 1.00), indicating greater blood loss compared to the cemented group (Hb change = 1.47 ± 0.98). Clinical blood loss was insignificant as none of the patients required transfusion. Conversely, the cementless group demonstrated statistically significant (*P* = 0.0056) shorter operative times of 1 hour and 44 minutes (±24 minutes and 40 seconds) compared to the cemented group of 2 hours and 1 minute (±19 minutes and 25 seconds) (Tables 1 and 2).

**Conclusion**

This study provides valuable insights into the intraoperative and perioperative outcomes of cemented and cementless robot-assisted TKA procedures. Surgeons should consider these factors when selecting the most appropriate technique for TKA, considering patient-specific characteristics and surgical goals.

**Reference**

1. Cementless Total Knee Arthroplasty Mackenzie Neumaier, David A Quinzi, Andrew Jeong, Rishi Balkissoon, 2024, Surgical Management of Knee Arthritis (pp.221–230)

**Table 1 (Abstract O42):** Cemented vs cementless TKA Hb levels

	Cemented (Mean, SD)	Cementless (Mean, SD)	*P* Value
Mean pre-operative Hb	13.8 ± 0.88	13.3 ± 1.03	0.0364
Mean post-operative Hb	12.3 ± 1.00	11.1 ± 1.00	<0.0001
Hb change	1.47 ± 0.98	2.15 ± 1.00	0.0112
Transfusion use (number of patients)	0 ± 0	0 ± 0	NA

**Table 2 (Abstract O42):** Cemented vs cementless robotic TKA mean surgical time and robot use time

	Cemented (Mean, HH:MM:SS,SD HH:MM:SS)	Cementless (Mean, HH:MM:SS, SD HH:MM:SS)	*P* Value
Mean total surgical time	13.8 ± 0.88	13.3 ± 1.03	0.0364
Mean ROSA use time	12.3 ± 1.00	11.1 ± 1.00	<0.0001

## P1 Analysis of synovial fluid WBC count and polymorphonuclear percentage after single-stage revision and DAIR with topical antibiotics infusion for periprosthetic hip and knee joint infection

### Wenbo Mu, Siyu Li, Chen Zou, Tuerhongjiang Wahafu, Wentao Guo, Boyong Xu, Li Cao

#### Department of Orthopaedics, First Affiliated Hospital of Xinjiang Medical University, Urumqi, China

##### **Correspondence:** Wenbo Mu (muwenbo8964@163.com)

*Arthroplasty 2024*, **6(Suppl 1):**P1

**Background**

This study introduces a novel approach of one-stage revision combined with intra-articular antibiotic injection for treating chronic PJI. However, the changes in synovial fluid analysis during the treatment are still unknown.

**Methods**

A retrospective analysis was conducted on 109 patients (51 hip joints and 60 knee joints) with chronic PJI who underwent single-stage revision or DAIR with topical antibiotics infusion in our department from January 2019 to March 2022 (successful treatment without recurrence). The trends in white blood cell count and percentage of polymorphonuclear cells in joint fluid before and after surgery (up to 14 days after surgery) were analyzed. The Mann-Whitney U test was used for inter-group comparison, and the statistical significance level was set at bilateral 0.05.

**Results**

The average white blood cell counts and percentage of polymorphonuclear cells in joint fluid before surgery were 16,750 × 10^6^/L and 90.5% respectively. During the 14-day treatment period after surgery, the WBC count in joint fluid decreased from 13,016 × 10^6^/L in the hip group and 18,641 × 10^6^/L in the knee group pre-operatively to 3286 × 10^6^/L and 3329 × 10^6^/L, respectively, on the 14th day after surgery. The percentage of PMN decreased from 87% in the hip group and 93% in the knee group pre-operatively to 84% and 80%, respectively, on the 14th day after surgery, with no statistically significant difference between the two groups. Among all patients, 56% had a general downward trend in joint fluid indicators after surgery, and 44% had a rebound increase after the decrease, with the second peak mainly appearing on the fifth day after surgery. The WBC count decreased from 20,061 × 10^6^/L in the Regain group and 16,050 × 10^6^/L in the Decrease group pre-operatively to 5045 × 10^6^/L and 1704 × 10^6^/L, respectively, on the 14th day after surgery. The percentage of PMN decreased from 85% in the Regain group and 86.5% in the Decrease group pre-operatively to 83% and 62%, respectively, on the 14th day after surgery. Gender, age, infection site, bacterial culture results, and drainage tube retention time were not risk factors for the second peak (Fig. 1).

**Conclusions**

The joint fluid indicators of PJI patients generally decreased within two weeks after surgery, but nearly 50% of patients showed a rebound in the indicators whilst the cause is still unclear and further analysis with a larger sample size is needed.

**Fig. 1 (Abstract P1):**
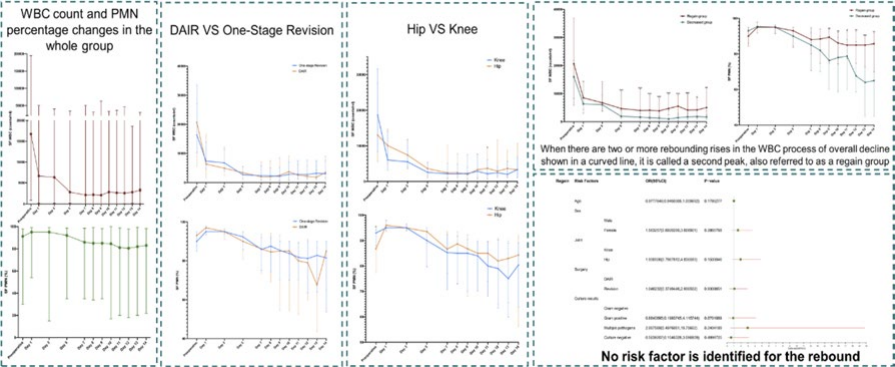
See text for description

## P2 3D printed acetabular prosthesis versus augment/bone graft+revision prosthesis for Paprosky III type bone defects

### Bin-Fei Zhang, Zhi Yang

#### Department of Joint Surgery, Honghui Hospital, Xi’an Jiaotong University, Xi’an, China

##### **Correspondence:** Bin-Fei Zhang (zhangbf07@gmail.com)

*Arthroplasty 2024*, **6(Suppl 1):**P2

**Background**

Type over this 3D-printed prosthesis was increasingly popular in the acetabular revision of THA, especially when the bone defect was irregular and large or osteolysis. This study aimed to evaluate the role of the 3D-printed prosthesis in revision THA in the mid-term, compared to conventional augment/bone graft+revision prosthesis.

**Methods**

In this retrospective study, the aseptic revision THA patients with prosthesis loosening and acetabular bone defects were screened between May 2010 and March 2015, and we included the patients receiving the 3D-printed acetabular prosthesis or augment/bone graft+revision prosthesis. The patients’ demographic characteristics of the patients were collected.

**Results**

A total of 79 patients (81 hips) with an acetabular revision using 3D-printed prosthesis or augment/bone graft+revision prosthesis were included. Thirty-six patients were men (45%), and 43 were women (55%); the mean age was 64.5 years (47 to 85), and the mean follow-up was 101 months (77 to 125). Thirty-eight hips (47%) had a Paprosky III A type defect, and 47 (53%) had a type III B defect. There were 33 (34 hips) and 46 (47 hips) patients in the 3D-printed prosthesis and the augment/bone graft+revision prosthesis groups, respectively. At the last follow-up, all hips in the 3D-printed prosthesis group remained well-fixed, and implant survival was 100%, with the need for re-revision as the endpoint. In contrast, five patients failed in the augment/bone graft+revision prosthesis group. The revision prosthesis aseptic loosening occurred in four patients, and prosthesis joint infection occurred in one patient. The patients with prosthesis joint infection died after one year of revision surgery. Excellent pain relief in all patients (mean WOMAC score pain 89.5 (37.7 to 100)) and functional outcomes (mean WOMAC function 89.3 (33.5 to 100), mean OHS 90.3 (32.1 to 100)) were noted. Patient satisfaction scores were excellent (Fig. 1).

**Conclusion**

This study demonstrated satisfactory mid-term clinical and radiological outcomes of using 3D-printed prosthesis in revision THA, compared to augment/bone graft+revision prosthesis.

**Fig. 1 (Abstract P2):**
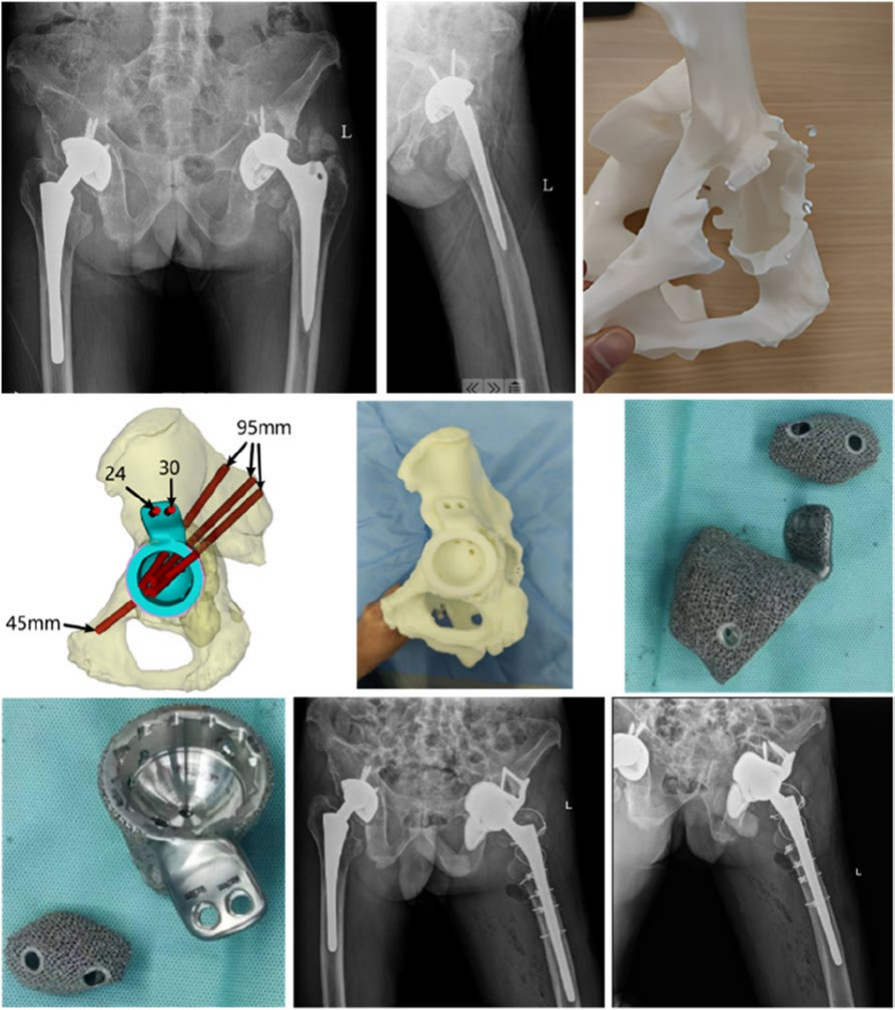
The case receiving 3D-printed acetabular prosthesis

## P3 Impaction bone grafting for both acetabular and femoral reconstructions in revision total hip arthroplasty

### Xinzhan Mao, Liang Xiong, Hui Li, Xianzhe Huang, Shuo Jie

#### Department of Orthopedics, The Second Xiangya Hospital of Central South University, Changsha, China

##### **Correspondence:** Xinzhan Mao

*Arthroplasty 2024*, **6(Suppl 1):**P3

**Background**

Extensive bone loss on both the acetabular and femoral sides has been a considerable challenge during total hip revision operations. Although multiple techniques have been reported in addressing this issue, few of them succeed in restoring bone stock fundamentally. To date, impaction bone grafting (IBG) is thought to be the only method that could effectively preserve bone mass and has showed satisfying outcomes in multiple studies [1]. In this study, we present our institutional experience in dealing with both severe acetabular and femoral defects by using IBG in revision total hip arthroplasty (THA).

**Methods**

Between October 2013 and December 2023, 19 patients (5 males, 14 females; mean age 66 years; range, 51 to 75 years) undergoing IBG revision on both the acetabular and femoral sides at our institution were analyzed. Bone defects were analyzed and classified using the Paprosky classification system. All bone grafts were taken from freeze-dried sliced femoral heads and morselized manually intraoperation. Postoperative complications and re-revision rates were evaluated.

**Results**

On the acetabular side, the defects were classified into Paprosky type IIIB in four cases, IIIA in four cases, IIC in eight cases, and IIB in three cases. On the femoral side, the defects were classified into Paprosky type IV in three cases, IIIB in four cases, IIIA in three cases, and IIB in nine cases. Postoperative complications were observed in six patients. Three developed deep vein thrombosis (DVT), two had femoral fractures and one had delayed wound healing. no patient had re-revision or operations related to the prosthesis at the latest follow-up (Figs. 1 and 2).

**Conclusion**

IBG in combination with cemented prosthesis is a profitable biological reconstruction revision technique that could provide satisfying midterm outcomes. We first propose the use of blood clots mixed with bone grafts for potential bone incorporation enhancement, while its specific effects need to be verified in further studies.

**Reference**

1. Wilson et al. Femoral impaction bone grafting in revision hip arthroplasty 705 cases from the originating centre. Bone Joint J 2016;98:1611e9.

**Fig. 1 (Abstract P3):**
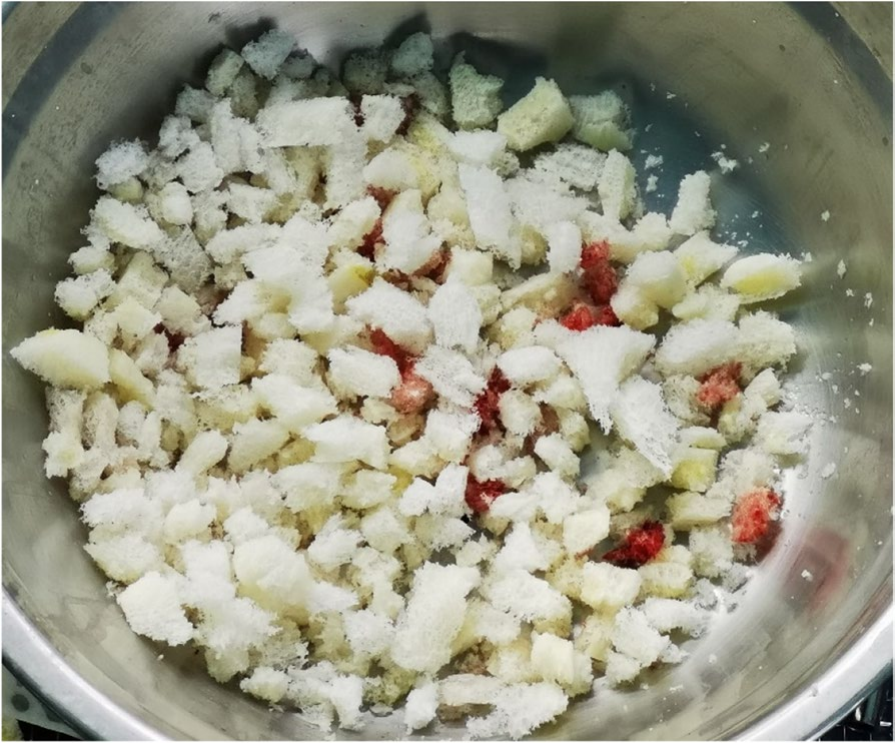
Intraoperative photograph of the prepared bone grafts

**Fig. 2 (Abstract P3):**
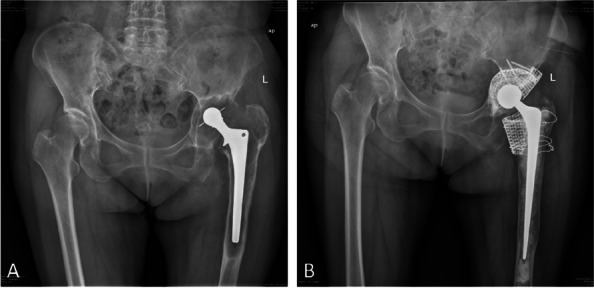
Preoperative (A) and postoperative (B) pelvic X-ray of one selected case

## P4 The combination of intraoperative tranexamic acid and postoperative single-dose anticoagulant protocol effectively reduces postoperative blood loss in revision hip arthroplasty: a retrospective cohort study

### Chenchen Yang, Li Cao

#### Department of Orthopaedics, First Affiliated Hospital of Xinjiang Medical University, Urumqi, China

##### **Correspondence:** Chenchen Yang (2596243576@qq.com)

*Arthroplasty 2024*, **6(Suppl 1):**P4

**Background**

Both intraoperative administration of tranexamic acid (TXA) and postoperative single-dose anticoagulant protocol (SDAP) have been shown to effectively reduce blood loss and transfusion rates in total joint arthroplasty. The purpose of this study is to evaluate the efficacy in blood loss of combining TXA with postoperative SDAP in revision hip arthroplasty.

**Methods**

We conducted a retrospective comparison of 815 patients who underwent revision hip arthroplasty between May 2002 and April 2024 and classified them into three groups: (1) the multiple-dose anticoagulant protocol (MDAP) group (40 mg low-molecular-weight heparin once and oral rivaroxaban for 10 days); (2) the intraoperative TXA combined with MDAP (TXA-MDAP) group (20 mg/kg/h intravenous TXA and 1 g topical TXA intraoperatively, and 40 mg low-molecular-weight heparin once with oral rivaroxaban for 10 days); (3) the intraoperative TXA combined with SDAP (TXA-SDAP) group (20 mg/kg/h intravenous TXA and 1 g topical TXA intraoperatively, and 40 mg low-molecular-weight heparin once). The postoperative decrease in hemoglobin, incidence of venous thromboembolism (VTE), and transfusion rate were recorded for each group.

**Results**

The postoperative decrease in hemoglobin showed significant differences among the three groups (*P* < 0.001, respectively). The transfusion rate was lower in both the TXA-SDAP and TXA-MDAP groups as compared to the MDAP group (*P* < 0.001, respectively). However, there were no significant differences observed between the transfusion rates of the TXA-MDAP and TXA-SDAP groups (*P* = 0.838). There were no significant differences found in VTE incidence between either the MDAP and TXA-MDAP groups (*P* = 0.059) or between the TXA-SDAP and TXA -MDAP groups (*P* = 0.057); however, the TXA-SDAP group reported a higher incidence of VTE compared with the MDAP group (*P* < 0.001) (Table 1).

**Conclusion**

Intraoperative TXA combined with a postoperative SDAP effectively reduced postoperative blood loss and transfusion rate in hip revision, albeit with an increased incidence of VTE.

**Table 1 (Abstract P4):** The postoperative decrease in hemoglobin of three groups

	MDAP	TXA-MDAP	TXA-SDAP	*P* Value
	(*n* = 221)	(*n* = 233)	(*n* = 361)	
Hemoglobin Difference (g/L)	39.35	34.88	27.59	<0.001

## P5 Comparative analysis of hemi-hip replacement outcomes: a study on femoral neck fractures versus intertrochanteric fractures

### Somchai Taosuwan, Varah Yuenyongviwat

#### Department of Orthopedics, Faculty of Medicine, Prince of Songkla University, Kho Hong, Songkhla, Thailand

##### **Correspondence:** Somchai Taosuwan (maximtao2@gmail.com)

*Arthroplasty 2024*, **6(Suppl 1):**P5

**Background**

Bipolar Hemiarthroplasty is the standard treatment for both femoral neck and intertrochanteric fractures in elderly patients. There are no comparative studies of bipolar hemiarthroplasty for femoral neck fractures and intertrochanteric fractures with a posterior approach in the same treatment setting procedure. Therefore, this study is conducted to obtain results that will guide patient preparation and provide advice to patients on making informed decisions [1–10].

**Methods**

A retrospective study was conducted based on collected medical records from Songklanagarind Hospital between 2013 to 2023. The study included two groups: 38 patients with intertrochanteric fractures and 152 patients with femoral neck fractures, all aged over 60 years, who underwent bipolar hemiarthroplasty. Data were collected to compare primary outcomes, including operative time, hospital stay, blood loss, complications, and ambulation between the two groups.

**Results**

Operating time was longer for intertrochanteric fractures (195 compared to 170 minutes). intertrochanteric fractures group underwent more complex procedures with wire, resulting in greater blood loss and more postoperative transfusions. Intraoperative fractures, particularly calcar fractures, were more common in the femoral neck group. The intertrochanteric group had a longer hospital stay (16.3 compared to 9.8 days) due to postoperative complications. Both groups experienced delayed ambulation (Figs. 1 and 2).

**Conclusion**

This research suggests that bipolar hemiarthroplasty for intertrochanteric fractures requires more preparation compared to femoral neck fractures. It involves the use of wire, blood transfusion units, specialized operative teams, and prolonged hospitalization. Hence, hemiarthroplasty for intertrochanteric fractures should be performed by specialists in hip surgery.

**References**

1. Mundi S, Pindiprolu B, Simunovic N, Bhandari M. Similar mortality rates in hip fracture patients over the past 31 years: a systematic review of RCTs. Acta Orthop. 2014;85(1):54-9.
2. American Academy of Orthopaedic surgeons [Internet]. Illinois. Plain Language summary: Hip fractures in older Adults; 2020 [cited 2024 May 15]; [about 2 screen]. Available from: https://www.orthoinfo.org/globalassets/pdfs/hip-fractures-cpg_pls.pdf
3. Gashi YN, Elhadi AS, Elbushra IM. Outcome of Primary Cemented Bipolar Hemiarthroplasty compared with Dynamic Hip Screw in Elderly Patients with Unstable Intertrochanteric Fracture. Malays Orthop J.2018 Mar;12(1): 36–41
4. Kathleen M, Jay M, J. Richard H, John E, and T. Michael. Intertrochanteric Versus Femoral Neck Hip Fractures: Differential Characteristics, Treatment, and Sequelae. J. Gerontol.1999;54A(12): 635–640
5. Qazi W,Misbah M.Early results of primary bipolar hemi arthroplasty in treatment of unstable intertrochanteric fractures in elderly patients: A prospective study. Int. J. Orthop. Sci. 2018; 4(3): 62–65
6. Kosuke T, MasahiroY, Daiki M, Tomoyuki N, Akihiko H, et al. Primary bipolar hemiarthroplasty as a treatment option for unstable intertrochanteric fractures. FMJ.2020: 6(4):122–127
7. Ponraj R, Senthilnathan A, Prabhakar R. Functional Outcome of Bipolar Hemiarthroplasty in Fracture Neck of Femur. Sch. J. Appl. Med. Sci. 2014; 2(5D):1785–1790.
8. Chhabra, Utkal G, Sanjay G. Functional outcome of bipolar hemiarthroplasty in fracture neck of femur. Int. J. Orthop. Sci.2020; 6(3): 32–36
9. Natthapong H, Patarawan W, Lertkong N, Sasivimol R, Ammarin T. Fracture fixation versus hemiarthroplasty for unstable intertrochanteric fractures in elderly patients: A systematic review and network meta-analysis of randomized controlled trials. Orthop Traumatol Surg Res.2022; 108 (1): 1–8
10. Jasveer S, Dinesh K, Sunil K, Ankit M, PradeepK, Ajay K et al. Functional Outcome of Hemiarthroplasty of the Hip for Unstable Intertrochanteric Fractures of the Femur in Elderly Patients: A Prospective Study. Cureus 2022; 14(12): e32526.

**Fig. 1 (Abstract P5):**
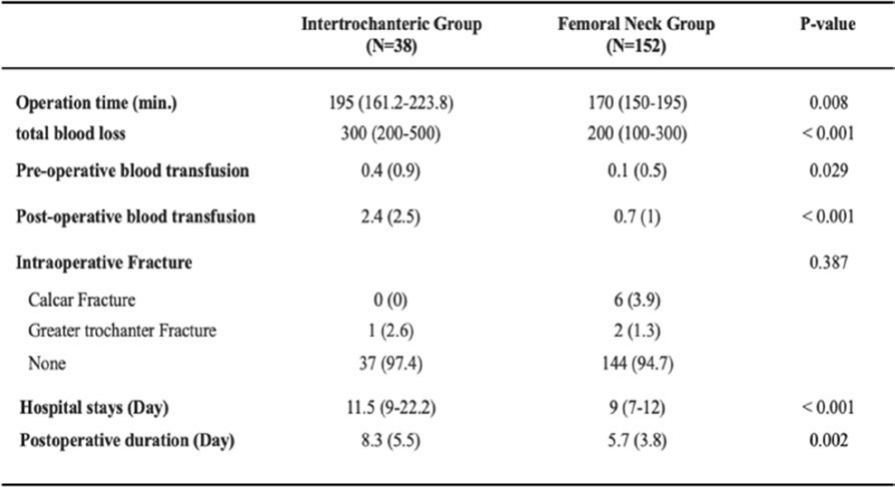
Intraoperative and postoperative

**Fig. 2 (Abstract P5):**
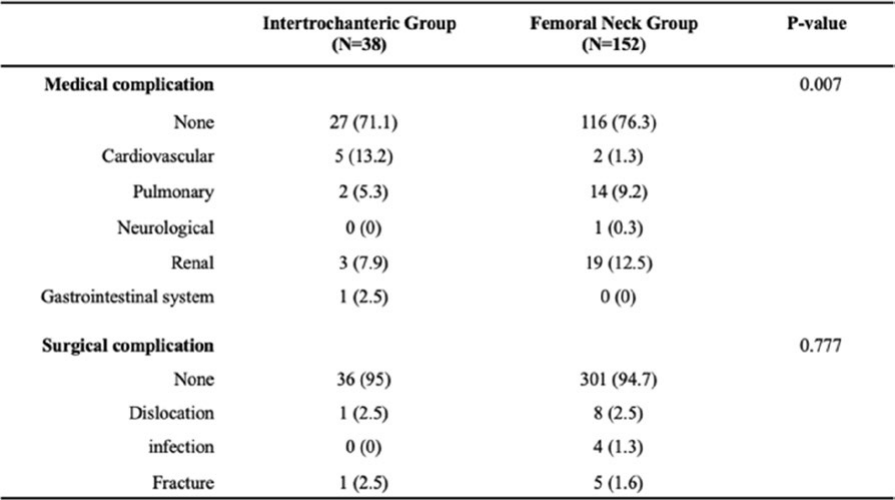
Postoperative complication

## P6 Enhanced CT analysis reveals fracture-specific muscle changes in F/U hip surgery patients: AI-driven artifact reduction and muscle segmentation

### Hyeon Su Kim, Shinjune Kim, Hyunbin Kim, Soojin Kim, Yoohee Lee, Hyun Jin Ju, Jun-Il Yoo

#### Department of Orthopedic Surgery, Inha University Hospital, Incheon, South Korea

##### **Correspondence:** Hyeon Su Kim (lemonjames96@gmail.com)

*Arthroplasty 2024*, **6(Suppl 1):**P6

**Background**

Hip fractures in the elderly pose significant health risks due to high morbidity and mortality. CT imaging is vital for evaluation but is often compromised by artifacts from metal implants and patient movement. This study explores an AI-based approach to enhance post-hip surgery CT scans, combining artifact reduction and muscle segmentation models. The aim is to improve the assessment of individual thigh muscles, potentially leading to better post-operative care and outcomes.

**Methods**

The study enrolled 50 patients, utilizing whole thigh CT scans from L3-L4 to the knee joint, assessing individual muscle volumes and intermuscular adipose tissue percentage pre- and post-operatively. We employed an AI-based artifact reduction model combined with a semantic segmentation model, developed using the UNETR architecture which achieved a Dice Coefficient (DC) of 0.84 [1,2]. This method enabled precise muscle segmentation and analysis of discrepancies between pre- and post-operative scans, focusing on the Piriformis and Obturator externus muscles.

**Results**

Analysis of follow-up CT scans revealed significant muscle disparities, particularly in intertrochanteric fracture patients. These patients exhibited markedly greater changes in piriformis and obturator externus muscles compared to those with femoral neck fractures, suggesting a correlation between fracture type and muscle-specific effects (Figs. 1 and 2).

**Conclusion**

The AI-based approach significantly improved CT scan clarity post-hip surgery. The study revealed that intertrochanteric fractures result in greater disparities in piriformis and obturator externus muscle volume and density compared to femoral neck fractures. These findings emphasize the need for targeted rehabilitation programs and surgical interventions to address the specific impact of fracture types on these muscles, potentially enhancing recovery outcomes.

**References**

1. Wu et al., Unsupervised Polychromatic Neural Representation for CT Metal Artifact Reduction. NeurIPS 2023.
2. Kim et al., Precise Individual Muscle Segmentation in Whole Thigh CT Scans for Sarcopenia Assessment Using U-net Transformer. Scientific Reports. 14:3301

**Fig. 1 (Abstract P6):**
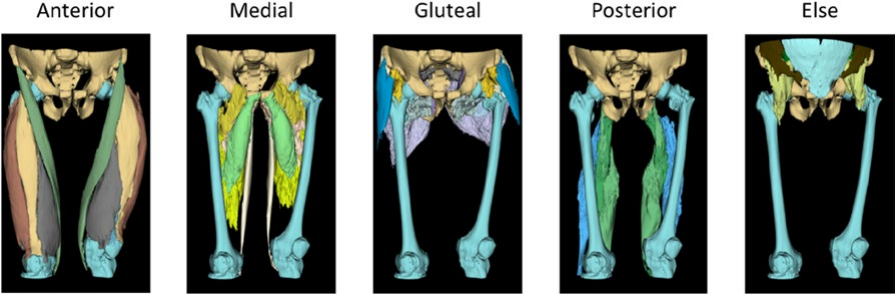
Segmentation 3D Rendering Image

**Fig. 2 (Abstract P6):**
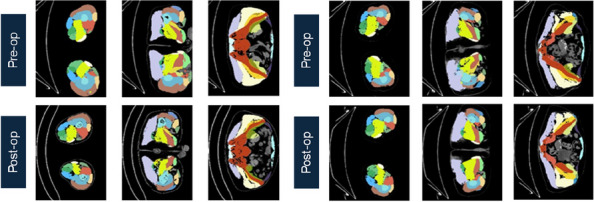
Result example of segmentation

## P7 Clinical efficacy of total hip arthroplasty with or without dual mobility cup versus hemiarthroplasty in elderly Asian patients with acute femoral neck fracture

### Jing Chen

#### Aerospace Center Hospital, Beijing, China

*Arthroplasty 2024*, **6(Suppl 1):**P7

**Objective**

To compare the clinical efficacy of total hip arthroplasty (THA) with or without dual mobility cup (DMC) versus hemiarthroplasty (HA) in elderly Asian patients with acute femoral neck fracture (FNF).

**Methods**

Data on 284 elderly patients who were treated for FNF at our institution from January 2017 to December 2021 were collected. Patients were divided into the DMC-THA group (THA with DMC, *n* = 102), C-THA group (THA without DMC, *n* = 88), and HA group (*n* = 94) according to treatment methods. Perioperative outcomes, hip functional recovery, treatment satisfaction, long-term prognosis, and quality of life were compared among the groups.

**Results**

The Harris scores of the DMC-THA group were significantly higher than those of the C-THA group and the HA group at 6 months, 1 year, and 3 years after surgery (*P* < 0.05). The treatment satisfaction rate of the DMC-THA group (92.2%) was significantly higher than those of the C-THA group (81.8%) and the HA group (80.9%, *P* < 0.05). At 1 year after surgery, the DMC-THA group had a significantly lower dislocation rate (2.0% vs 9.1%) and a significantly better mobility than the C-THA group (*P* < 0.05). Mobility and pain/discomfort were significantly better in the DMC-THA group than in the HA group (*P* < 0.05) (Tables 1 and 2).

**Conclusion**

Compared with conventional THA and HA, THA with DMC has the advantages of better joint function recovery, lower dislocation rate, and higher quality of life, which is expected to become the preferred replacement program for elderly patients with acute FNF.

**Table 1 (Abstract P7):** Comparison of the perioperative outcomes among the three groups

Group	Operation time (min)	Blood loss (mL)	Postoperative drainage (mL)	Blood transfusion	Time to ambulation	Length of stay
DMC-THA group (*n* = 102)	86.4 ± 26.6	285 ± 53	128 ± 24	11 (10.8%)	3.9 ± 1.3	11.4 ± 3.1
C-THA group (*n* = 88)	88.2 ± 24.1	296 ± 60	131 ± 25	10 (11.4%)	4.2 ± 1.4	11.8 ± 3.4
χ^2^/t	0.486	1.342	0.843	0.016	1.531	0.848
*P* value	0.628	0.181	0.400	0.899	0.128	0.398
HA group (*n* = 94)	76.8 ± 24.5	257 ± 48	122 ± 20	9 (9.6%)	4.2 ± 1.1	11.7 ± 2.9
χ^2^/t	2.621	3.865	1.893	0.078	1.737	0.698
*P* value	0.009	<0.001	0.060	0.780	0.084	0.486

**Table 2 (Abstract P7):** Comparison of the recovery of hip function among the three groups

Group	Preoperative	6 months after surgery	1 year after surgery	3 years after surgery
DMC-THA group (*n* = 102)	24.7 ± 6.1	76.6 ± 8.7	84.2 ± 10.0	89.5 ± 9.1
C-THA group (*n* = 88)	23.9 ± 5.4	72.3 ± 10.3	80.6 ± 8.8	86.2 ± 9.7
t	0.950	3.136	2.615	2.417
*P* value	0.343	0.002	0.010	0.017
HA group (*n* = 94)	24.3 ± 5.3	70.1 ± 8.5	80.4 ± 11.3	85.0 ± 10.2
t	0.488	5.316	2.497	3.264
*P* value	0.626	<0.001	0.013	0.001

## P8 A novel method for prosthetic position planning in total hip arthroplasty

### Tao Feng^1^, Xiaogang Zhang^1^, Yali Zhang^1^, Zhongmin Jin^1,2^

#### ^1^Tribology Research Institute, Southwest Jiaotong University, Chengdu, China; ^2^School of Mechanical Engineering, University of Leeds, Leeds, UK

##### **Correspondence:** Tao Feng (sherlockfeng@my.swjtu.edu.cn)

*Arthroplasty 2024*, **6(Suppl 1):**P8

**Background**

Preoperative for total hip arthroplasty (THA) significantly reduces postoperative impingement and dislocation. However, most current preoperative planning considers a single factor. Therefore, we developed an algorithm that quickly calculates the hip’s impingement-free range of motion (IFROM) after THA. Combined with the finite element model (FEM), the algorithm can consider bony impingement, non-standard-shaped prostheses, the pelvic position, the coverage of the cup, and the hip capsule. Based on these methods, we aimed to investigate (1) the effect of stem neck offset on combined anteversion (CA) and (2) the effect of the capsule on the liner’s edge loading and contact stress.

**Methods**

After obtaining 3D models of the patient’s skeleton and prosthesis and initializing their positions, the algorithm used the surface fitting points of these models to perform matrix transformations to simulate the corresponding spatial positions, followed by impingement detection to calculate the patient’s personalized IFROM and impingement-free safe zone (IFSZ). We calculated IFROM and IFSZ for different stem neck offsets (−4, 0, +4 mm) and prosthesis mounting positions. Additionally, FEM was employed to calculate liner contact stresses and edge loading at different mounting positions and different gaits.

**Results**

We found that (1) an increase in stem neck offset under the same IFSZ criteria decreases the difference between the upper and lower limits of the CA at a rate of 1.75 °/mm; (2) preservation of the hip capsule decreases or even retards edge loading on the liner, and (3) excessive cup inclination and anteversion worsen edge loading on the liner (Fig. 1).

**Conclusion**

Adjustment of the stem neck offset in THA is accompanied by adjustment of the CA; preservation and suturing of the hip capsule in THA improve prosthesis contact mechanics and longevity. Most importantly, our method provides a reliable tool for personalized fitting planning of prostheses in hip patients.

**Fig. 1 (Abstract P8):**
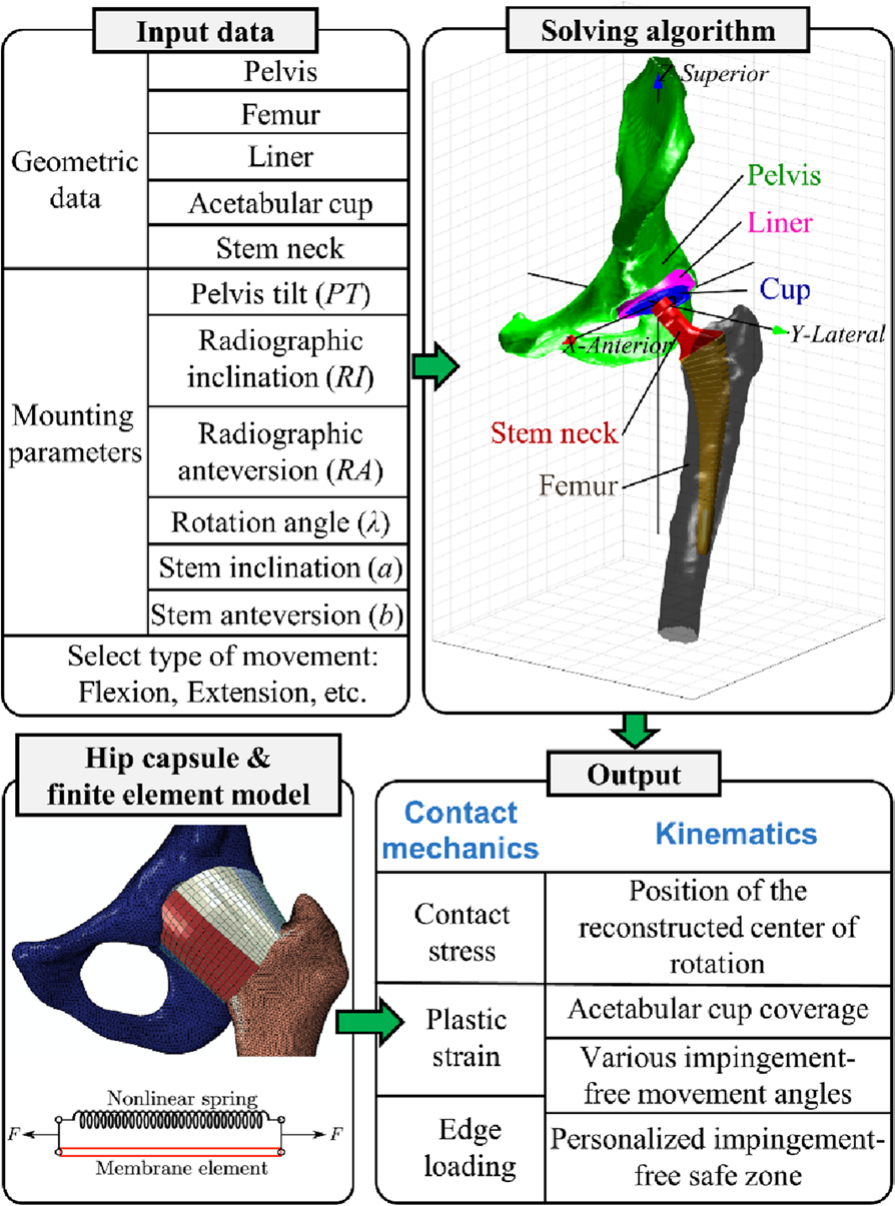
Solution procedure for the prosthetic and contact mechanics of the hip joint

## P9 Patellar replacement in TKA has good long-term effectiveness: a randomized controlled trial

### Te Liu, Runkai Zhao, Ye Tao, Qingyuan Zheng, Guoqiang Zhang, Ming Ni

#### Department of Orthopedics, The Fourth Medical Center, Chinese PLA General Hospital, Beijing, China

##### **Correspondence:** Ming Ni (niming301@163.com)

*Arthroplasty 2024*, **6(Suppl 1):**P9

**Background**

The long-term outcomes of patellar replacement remain unclear. This study aims to investigate the long-term result of patellar replacement in TKA (total knee arthroplasty) [1].

**Methods**

We conducted a single-center, prospective, randomized, controlled trial. We enrolled 36 knees from 18 patients who underwent bilateral TKA between March 2013 and May 2014. One patellar replacement (PR) and another did not undergo patella (NPR) replacement. The joint snapping, satisfaction of the patients, anterior knee pain (AKP), and KSS score were evaluated 6 weeks after surgery and at the last follow-up. Reoperations due to Patellofemoral joint problems, patellar polyethylene wear, and complications of patellar necrosis were recorded. Kaplan-Meier survival analysis was used to analyze patellar survival.

**Results**

The average follow-up time was 10 years. The incidence of knee joint snapping was significantly lower on the replacement side than on the non-replacement side at 6 weeks after surgery and at the last follow-up (*P* < 0.001; *P* < 0.001); Patient satisfaction on the replacement side was significantly higher than that on the non-replacement side 6 weeks after surgery and at the last follow-up (*P* < 0.001; *P* < 0.001). The VAS score of anterior knee pain on the replacement side was lower 6 weeks after surgery, which was statistically significant (*P* = 0.04). The VAS score of anterior knee pain on the replacement side was lower at the last follow-up, but there was no statistical difference (0.71). There was no statistical difference in KSS scores between the two sides at 6 weeks after surgery and the last follow-up (*P* = 0.16; *P* = 0.32); All patients had no patellar complications, and the survival rate of both knees was 100% when the endpoint event was defined as reoperation (Figs. 1 and 2).

**Conclusion**

Patellar replacement in TKA has better long-term overall feeling than non-replacement, and both have good prosthesis survival rates.

**Reference**

1. Gunderson ZJ, Luster TG. The Fate of Unresurfaced PatellaeinContemporary Total Knee Arthroplasty: Early to Midterm Results. J Arthroplasty2024:S0883540324000822. 10.1016/j.arth.2024.01.055.

**Fig. 1 (Abstract P9):**
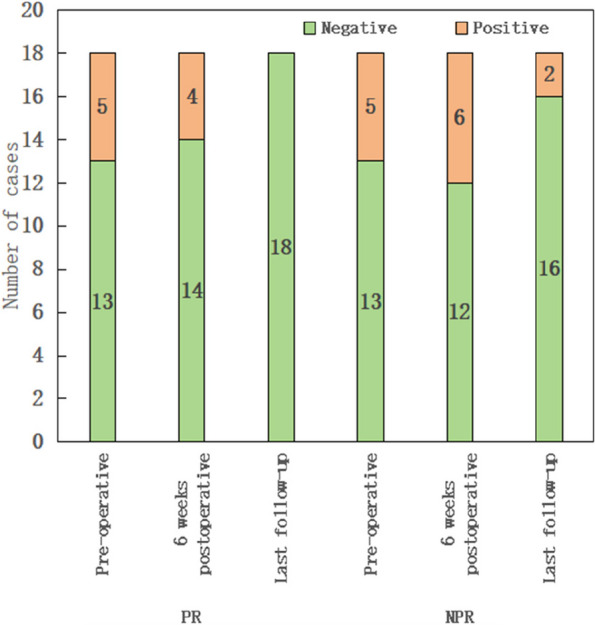
Comparison of patients’ joint snapping

**Fig. 2 (Abstract P9):**
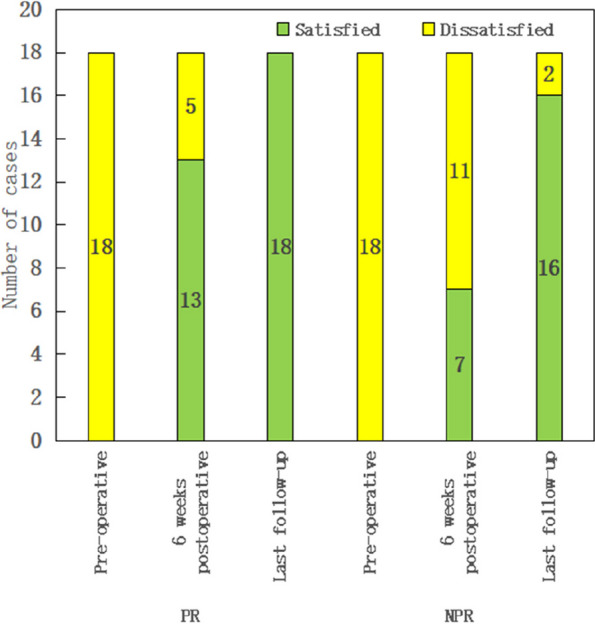
Comparison of patient satisfaction

## P10 A multi‐phase approach for developing a conceptual model and preliminary content for patient-reported outcome measurement in TKA patients: from a Chinese perspective

### Chao Xu, Shuxin Yao, Jianbing Ma

#### Department of Knee Joint Surgery, Honghui Hospital, Xi’an Jiaotong University, Xi’an, China

##### **Correspondence:** Jianbing Ma (drmajianbing@163.com)

*Arthroplasty 2024*, **6(Suppl 1):**P10

**Objective**: Patient-reported outcome measures (PROMs) are being used more frequently in total knee arthroplasty (TKA). By utilizing high-quality scales, surgeons can achieve a more comprehensive and accurate evaluation of the effectiveness of TKA surgery. Currently, there is no widely accepted conceptual model for TKA PROMs. The objective of this study is to fill this gap by developing a conceptual model and preliminary content for a PROM that is specifically designed for TKA patients in mainland China.

**Methods**: The study design consisted of three stages: 1) a targeted literature review followed by the formation of a conceptual model pool; 2) qualitative data collection involving experts and patients, leading to the development of the preliminary Chinese TKA PROM (CTP); and 3) review of the CTP by experts using the Delphi method, along with cognitive debriefing interviews with patients.

 Results: 64 patients and 28 experts took part in this study. The conceptual model focused on six key concepts: pain, symptom, function, quality of life, expectation, and satisfaction. To match the model, a total of 35 items were created in the CTP.

**Conclusion**: A conceptual model and preliminary content for CTP was developed with substantial participation from patients and a multidisciplinary group of experts. The integration of patient and clinical perspectives ensured a comprehensive representation of all relevant disease experiences and the focus of clinical practice. With further refinement through psychometric testing, the CTP is positioned to provide a standardized, comprehensive measure for research specific to Chinese TKA patients.

## P11 Home-based is safe as traditional rehabilitation following TKA: a systematic review and meta-analysis of randomized controlled trials

### P.I. Fiore^1^, M. Oldrini^1^, D. Previtali^1^, S. Tamborini^1^, G. Filardo^1,2^, C. Candrian^1,2^

#### ^1^Service of Orthopaedics and Traumatology, Department of Surgery, EOC, Lugano, Switzerland; ^2^Faculty of Biomedical Sciences, Università della Svizzera Italiana, Lugano, Switzerland

##### **Correspondence:** P.I. Fiore (lorenzomassimo.oldrini@eoc.ch)

*Arthroplasty 2024*, **6(Suppl 1):**P11

**Background**

To directly compare home-based vs traditional rehabilitation programs following TKA, to prove if an unsupervised approach leads to similar clinical and functional results as the usual rehabilitation standard-of-care.

**Methods**

A comprehensive literature search was performed on the Pubmed, Web of Science, and Wiley Cochrane Library databases up to November 18, 2022. RCTs describe addressing home-based vs inpatient rehabilitation following TKA. A systematic review and meta-analysis were performed on clinical and functional outcomes. Assessment of the risk of bias and quality of evidence was performed with the “Cochrane Collaboration Risk of Bias tool”.

**Results**

Twenty-three studies including 3946 patients were included. The home protocol was used by 1986 patients, while 1960 patients underwent supervised rehabilitation. In the latter group, short-term showed significant improvement (*P* < 0.05) in terms of SF-36, OKS, and ROM downturn compared with baseline values, whereas with the home protocol, these outcomes were not found. In contrast, in the long term, all outcomes analyzed showed statistically significant improvement over baseline values in both groups (*P* < 0.05).

**Conclusion**

This meta-analysis and systematic review did not demonstrate the non-inferiority of unsupervised rehabilitation compared with supervised rehabilitation post-TKA. However, it did find that supervised rehabilitation is associated with superior functional outcomes and faster quality-of-life recovery than unsupervised rehabilitation.

## P12 A case of simultaneous bilateral MRSA infection after TKA successfully treated with continuous local antibiotics perfusion

### Kohei Motono, Tomoyuki Matsumoto, Masanori Tsubosaka, Ryosuke Kuroda

#### Department of Orthopaedic Surgery, Kobe University Graduate School of Medicine, Kobe, Japan

##### **Correspondence:** Kohei Motono (mmoko0422@gmail.com)

*Arthroplasty 2024*, **6(Suppl 1):**P12

**Background**

Continuous local antibiotics perfusion (CLAP) is a procedure in which a bone marrow needle and a double-lumen tube are placed in the infected area, and an appropriate concentration agent is continuously administered and perfused. CLAP has shown good outcomes for bone and soft tissue infections. However, reports of using CLAP for prosthetic joint infection (PJI) after total knee arthroplasty (TKA) are rare. We present a case of simultaneous bilateral Methicillin-Resistant Staphylococcus Aureus (MRSA) infection after TKA was successfully treated with CLAP.

**Case presentation**

The patient is a 69-year-old male with cerebral palsy. He had a history of left TKA one year ago and right TKA nine months ago. He had experienced difficulty walking for the last two weeks and had a fever for three days. Both knees show swelling, pain, and redness without wound drainage. The leukocyte count was 12,820/μL, and the C-reactive protein was 16.27 mg/dL. Bilateral joint aspirations revealed gram-positive cocci, leading to a diagnosis of bilateral PJI. Imaging shows no implant loosening. We performed simultaneous debridement, irrigation, and insert exchange surgery on both knees, combined with CLAP using two double-lumen tubes placed on both sides of the knee joint. Intraoperative culture identified MRSA. Gentamicin was administered intra-articular via the double-lumen tubes for 14 days postoperatively, and vancomycin was administered intravenously for 40 days. The patient was switched to oral minocycline after that. At least six months postoperatively, blood tests and clinical findings haven’t shown an elevated inflammatory response that would raise suspicion of infection, and there has been no recurrence of PJI (Fig. 1). The patient gave their informed written consent to publish their information in an open access journal.

**Discussion**

Simultaneous bilateral PJI after TKA is rare, and PJI caused by MRSA has a high infection recurrence rate. Although long-term observation is necessary, this case demonstrates the possibility that CLAP can effectively control PJI caused by MRSA and retain knee implants.

**Conclusion**

CLAP could be a viable treatment option for PJI caused by MRSA after TKA.

**Fig. 1 (Abstract P12):**
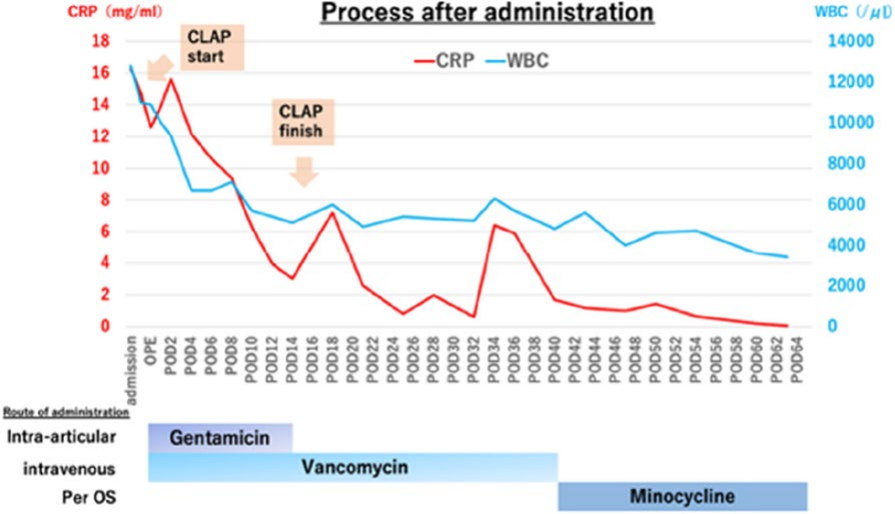
The course of blood test data after surgery, the route, and the time of antibiotic administration

## P13 Single-stage debridement and arthroplasty for septic arthritis in native end-stage knee osteoarthritis: minimal two-year follow-up

### Weijun Wang, Minghao Zhang, Yuhao Yang, Qing Jiang

#### Nanjing Drum Tower Hospital, The Affiliated Hospital of Nanjing University Medical School, Nanjing, China

##### **Correspondence:** Weijun Wang (drwilliamwang@163.com)

*Arthroplasty 2024*, **6(Suppl 1):**P13

**Background**

Two-stage treatment protocol, irrigation, and debridement, followed by second-staged joint arthroplasty, has been widely used to treat septic arthritis (SA) in patients suffering from end-stage knee osteoarthritis. Limitations of this protocol included at least twice hospital stay and operation, increased cost, and may lead to poor functional outcomes. High success rate in single-stage revision for periprosthetic joint infection has been achieved in our center. Hence, the same single-stage arthroplasty was applied in managing SA associated with end-stage knee osteoarthritis. Whether single-stage debridement and arthroplasty could be safe and effective in treating SA in native end-stage knee osteoarthritis?

**Methods**

A retrospective analysis of the prospective nature of patients who suffered SA and end-stage knee osteoarthritis treated with single-stage debridement and arthroplasty were carried out. Patients with at least two-year follow-ups were recruited. SA was diagnosed by elevated serum ESR, CPR and positive finding in synovial fluid culture. The surgical procedure started with aggressive debridement together with bone cutting for reconstructive arthroplasty. Sensitive antibiotics were used in both intravenous and topical approaches after the operation, and the serum ESR, CRP, synovial fluid cell count, and CRP were used to monitor the control of infection. Oral antibiotics administration was used till 3 months after operation. Functional outcome was assessed by KSS.

**Results**

Seven patients with a mean age of 61.3 years were included. All patients were implanted with primary prosthesis. The mean operation time was 156 min, mean duration of hospital stay was 18.4 days. The pathogens included staphylococcus in six patients and staphylococcus combined with streptococcus in one. The vancomycin and rifampicin were given for infection treatment. Patients were followed for a mean of 27.9 months. No recurrence of postoperative infection and no reoperation after prosthesis implantation was observed. The mean KSS significantly improved from 32.1 ± 19.3 points pre-operatively to 83.4 ± 10.9 points at final follow-up (*P* = 0.000036).

**Conclusion**

The single-stage debridement and arthroplasty could be employed for treating SA in native end-stage knee osteoarthritis in surgeons experienced in managing periprosthetic joint infection with single-stage revision.

## P14 The natural progression of white blood-cell counts and the percentage of polymorphonuclear in synovial fluid during the perioperative period following primary total knee arthroplasty: a longitudinal study

### Xiaobin Guo, Xiaogang Zhang, Li Cao

#### Department of Orthopaedics, First Affiliated Hospital of Xinjiang Medical University, Urumqi, China

##### **Correspondence:** Li Cao (xjbone@sina.com)

*Arthroplasty 2024*, **6(Suppl 1):**P14

**Background**

Periprosthetic joint infection (PJI) is a severe complication following arthroplasty. As the most widely used biomarkers in synovial fluid for diagnosing PJI, the natural progression of white blood-cell (WBC) counts and percentage of polymorphonuclear (PMN) cells following primary TKA have never been adequately documented and described. This prospective longitudinal study aims to elucidate the dynamic changes of WBC counts and PMN percentage in synovial fluid during the perioperative period in patients undergoing primary TKA, and to explore their clinical significance.

**Methods**

From April 2023 to April 2024, 118 patients voluntarily participated in this prospective clinical study. Serial knee aspirations were performed on these patients at multiple time points: prior to primary TKA and subsequently on postoperative days 3, 5, 14, 21, and 28 days. All these samples were subjected to count analysis to dynamically record the natural progression of WBC counts and PMN percentage during the perioperative period of TKA.

**Results**

All 108 patients were free from PJI during the 3-month follow-up period after surgery. Compared to preoperative levels, Sy-WBC peaked on the third day postoperatively and then gradually decreased (857.51 ± 1794.76; 8702.13 ± 5832.22; 3434.69 ± 2593.86; 1305.39 ± 1536.87; 645.99 ± 804.09; 613.77 ± 863.32, respectively). There were no statistically significant differences in Sy-WBC levels on the 14th, 21st, and 28th days postoperatively compared to preoperative levels. Rheumatoid arthritis patients had higher Sy-WBC counts than osteoarthritis patients, showing statistically significant differences preoperatively and during the first three weeks postoperatively. The PMN percentage similarly peaked at 3 days post-operation, then gradually decreased (23.23 ± 18.17; 94.47 ± 8.99; 88.08 ± 7.59; 64.15 ± 20.20; 41.13 ± 20.10; 30.12 ± 20.02, respectively). By 28 days post-operation, there was no statistically significant difference compared to preoperative levels. There was no statistically significant difference in the percentage of multinucleated cells in rheumatoid arthritis patients (Figs 1 and 2).

**Conclusion**

This comprehensive approach of analyzing synovial fluid components at various perioperative time points provides valuable data for assessing inflammatory and immune responses, enabling effective monitoring of postoperative recovery and potential complications following total knee arthroplasty.

**Fig. 1 (Abstract P14):**
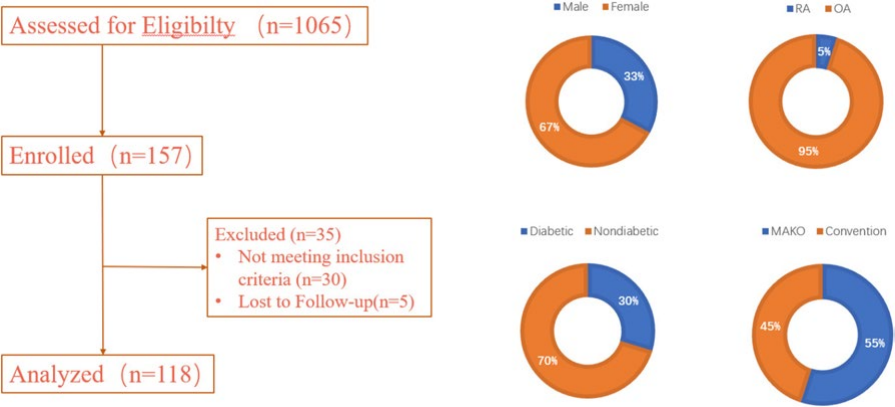
Flowchart showing the constitution of the cohort

**Fig. 2 (Abstract P14):**
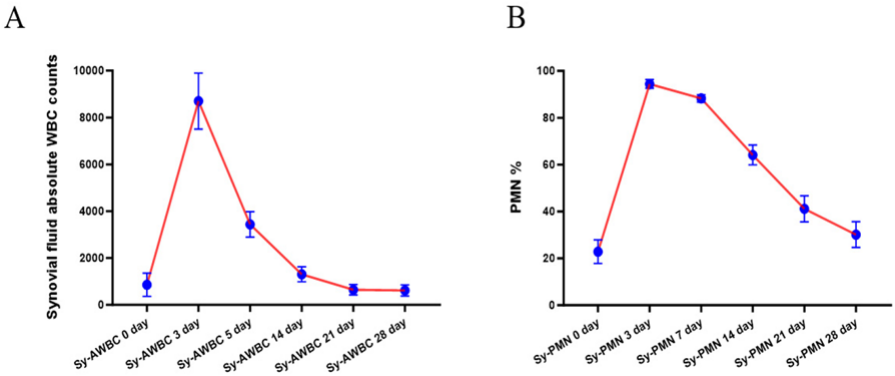
Perioperative trends in Sy-AWBC(**A**) and Sy-PMN(**B**)

## P15 Clinical outcomes of autologous adipose-derived mesenchymal stem cell therapy in treating knee osteoarthritis are correlated with stem cell quantity and quality

### Houyi Sun^1,2^, Peilai Liu^1,2^

#### ^1^Department of Orthopaedics, Qilu Hospital, Cheeloo College of Medicine, Shandong University, Jinan, China; ^2^Department of Orthopaedics, Qilu Hospital of Shandong University Dezhou Hospital, Dezhou, China

##### **Correspondence:** Houyi Sun (15051514605@163.com)

*Arthroplasty 2024*, **6(Suppl 1):**P15

**Background**

Mesenchymal stem cells (MSCs) have been proposed to treat osteoarthritis (OA) for many years. However, clinical outcomes have been inconsistent due to biological variation between patients, differences in tissue source and preparation of the MSCs, and type of donor (e.g., allogenic versus autologous). Here, we test the hypothesis that inconsistent clinical outcomes are related to variations in the quantity and quality of the injected autologous adipose-derived (AD) MSCs.

**Methods**

Forty-five knee OA patients were divided into 2 groups: Group 1 (*n* = 22) patients treated with high tibial osteotomy (HTO) alone and Group 2 (*n* = 23) patients treated with HTO followed by intra-articular injection of autologous AD-MSCs (HTO+AD-MSCs). We observed the proliferation and stemness of AD-MSCs selected from the 5 patients showing the most improvement and from the 5 patients with the least improvement and completed further in vitro experiments including beta-galactosidase activity, reactive oxygen species, and bioinformatic Analysis.

**Results**

The results showed that patients treated with HTO+AD-MSCs significantly reduced knee OA severity compared to patients treated with HTO alone. Moreover, we discovered that the proliferation and colony-forming efficiency of AD-MSCs selected from the 5 patients showing the most improvement performed significantly better than cells selected from the 5 patients with the least improvement. AD-MSCs from the patients with the most improvement also had lower amounts of senescent cells and intracellular reactive oxygen species (Figs 1 and 2).

**Conclusion**

Clinical outcomes of autologous AD-MSCs therapy in knee osteoarthritis are correlated with stem cell quantity and quality. Our study highlights emerging opportunities and trends in precision medicine that could potentially improve autologous MSC-based therapies.

**Fig. 1 (Abstract P15):**
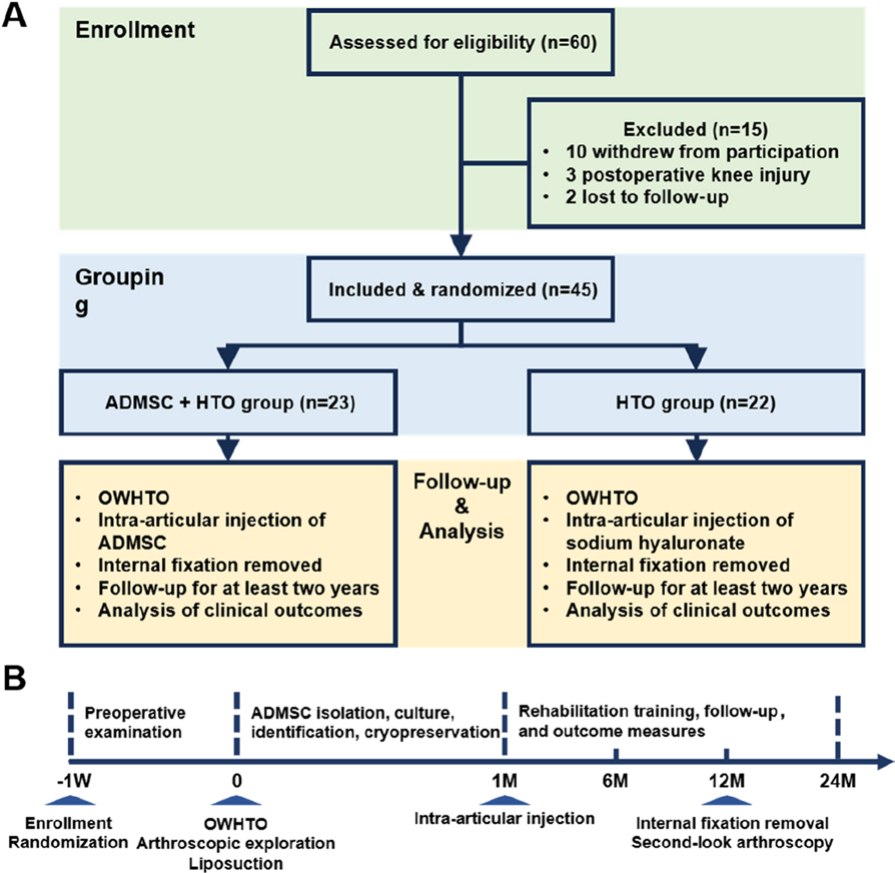
Flow chart of OA patients’ enrollment and follow-up protocol

**Fig. 2 (Abstract P15):**
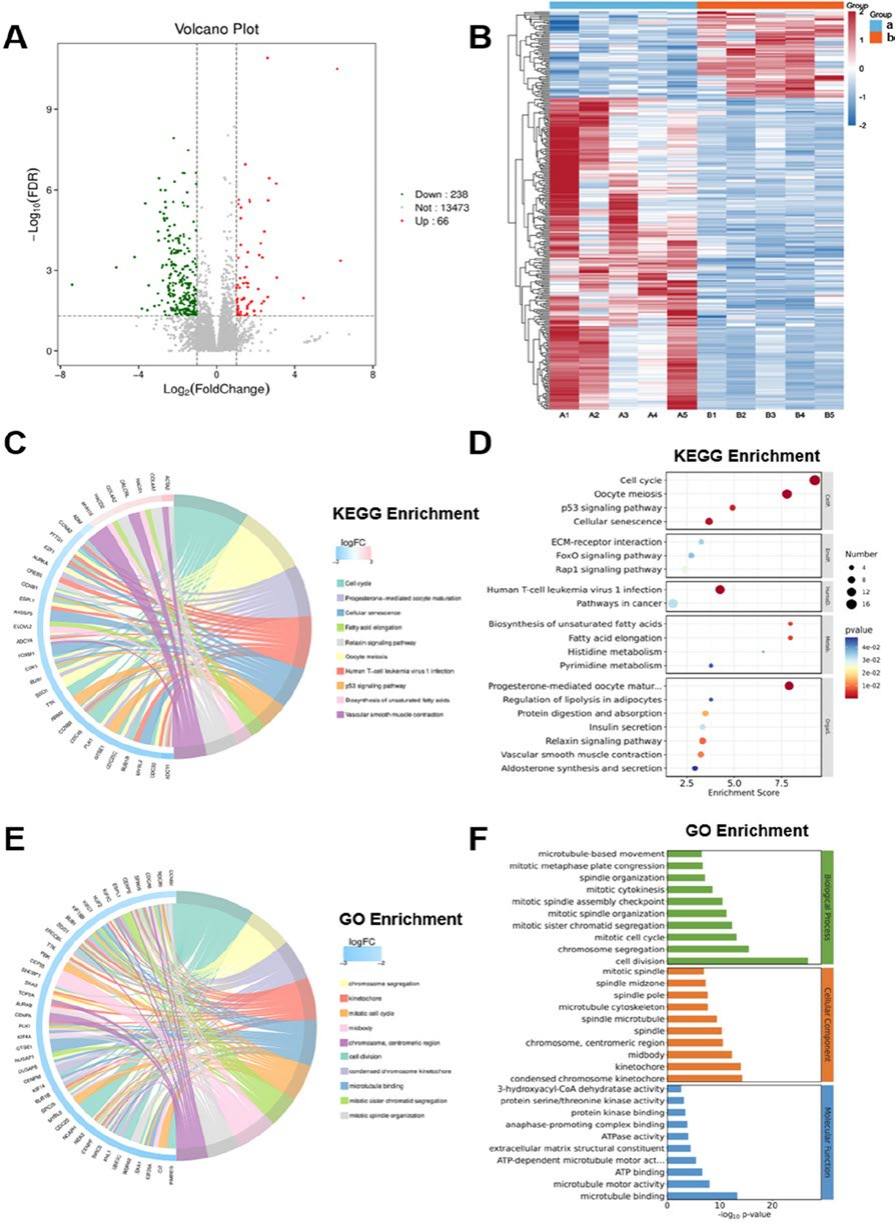
RNA-Sequencing of AD-MSCs from sub-groups a and b

## P16 Comparison of the clinical results with three different dosages of topical tranexamic acid injection during total knee arthroplasty

### Jae Ang Sim, Kyung-Sik Kong, Byung Hoon Lee

#### Department of Orthopaedics Surgery, Gachon University College of Medicine, Incheon, South Korea

##### **Correspondence:** Byung Hoon Lee (oselite@naver.com)

*Arthroplasty 2024*, **6(Suppl 1):**P16

**Background**

To compare three-dose regimens based on clinical effectiveness and safety within 30 days of surgery of three different doses of IA-TXA used in unilateral and simultaneous bilateral TKA.

**Methods**

A retrospective search of 689 patients who underwent either unilateral or simultaneous bilateral primary TKA between 2021 and 2023, was performed. Patients were divided into three groups: 500 mg, 1-g, and 2-g intra-articular TXA during wound closure. This regimen was applied equally in the simultaneous bilateral TKAs. Postoperative hemoglobin levels, transfusion rates, and the amount of drained blood were assessed to determine the clinical effectiveness of topical IA-TXA. The VTE incidence was also analyzed to compare the safety of different dosages of IA-TXA.

**Results**

The group of 1-g dose (Hb decline 1.6–2.2 g/dL) or 2-g dose (Hb decline 1.7–2.5 g/dL) of IA-TXA showed significantly lower postoperative Hb drop at 2 days postoperative than the group of 500 mg dose (Hb decline 2.5–3.0 g/dL) of IA-TKA in unilateral TKA. Also, in simultaneous bilateral TKA, the group of 500 mg dose (Hb decline 1.1–4.6 g/dL) of IA-TKA showed significantly higher postoperative hemoglobin declines than both 1-g group (Hb decline 0.5–2.8 g/dL) and 2-g group (Hb decline 0.6–2.4 g/dL) of IA-TXA for postoperative 7 days. However, no notable differences were observed between the 1-g and 2-g group in either unilateral or bilateral TKA. Transfusion rate in unilateral TKA was higher in the 500 mg dose group (11.3%) than in the 1-g dose (5.2%) and in the 2-g dose group (8.1%) (all *P* < 0.001), with no significant difference between the 1-g dose and 2-g dose groups (*P* = 0.460). There were no significant differences in VTE prevalence among the three groups. Despite doubling the topical volume for simultaneous bilateral TKA, the results were consistent with those of unilateral TKA (Figs 1 and 2).

**Conclusion**

The application of ≥1.0 g of topical TXA was more effective than 0.5 g in reducing blood loss and transfusion occurrences without increasing the VTE risk, whether for a unilateral or bilateral TKA.

**Fig. 1 (Abstract P16):**
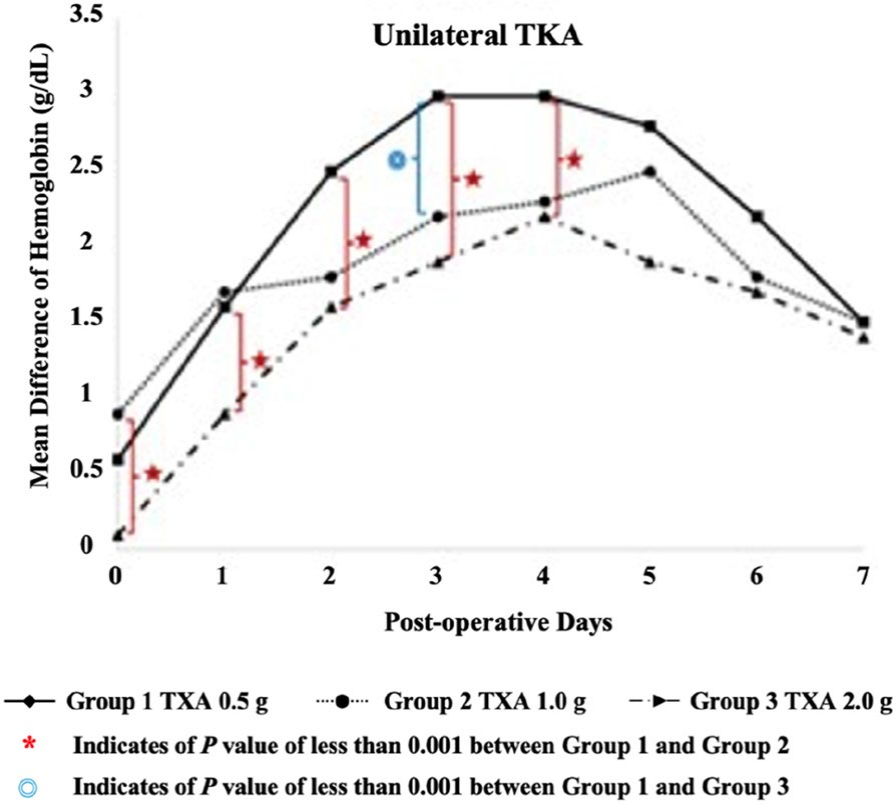
A scatter plot comparing the mean decline in hemoglobin (Hb) on each postoperative day among different doses of intra-articular Tranexamic Acid (TXA): Group 1 (0.5 g TXA), Group 2 (1.0 g TXA), and Group 3 (2.0 g TXA) in simultaneous bilateral TKA. An asterisk (*) indicates a significant difference between Group 1 and Group 2, whereas a circle (◎) indicates a significant difference between Group 1 and Group 3

**Fig. 2 (Abstract P16):**
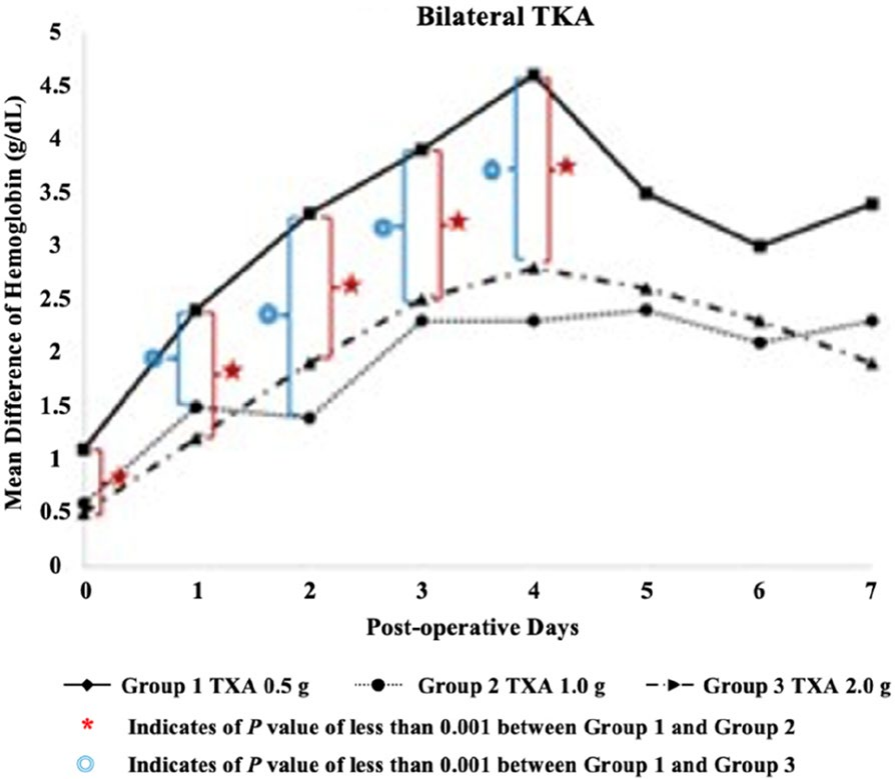
A scatter plot comparing the mean decline in hemoglobin (Hb) on each postoperative day among different doses of intra-articular Tranexamic Acid (TXA): Group 1 (0.5 g TXA), Group 2 (1.0 g TXA), and Group 3 (2.0 g TXA) in unilateral TKA. An asterisk (*) indicates a significant difference between Group 1 and Group 2, whereas a circle (◎) indicates a significant difference between Group 1 and Group 3

## P17 Remote neuromuscular electrical stimulation effects on bilateral TKA rehabilitation

### Runkai Zhao, Te Liu, Quanbo Ji, Guoqiang Zhang

#### Department of Orthopedics, The Fourth Medical Center, Chinese PLA General Hospital, Beijing, China

##### **Correspondence:** Guoqiang Zhang (gqzhang301@163.com)

*Arthroplasty 2024*, **6(Suppl 1):**P17

**Background**

Total knee arthroplasty (TKA) is a common surgical intervention for end-stage knee osteoarthritis, aiming to alleviate pain and improve function. Postoperative rehabilitation plays a crucial role in achieving optimal outcomes. NMES is a technique that involves applying electrical impulses to stimulate specific muscles, which can help prevent muscle atrophy and promote muscle strength and joint mobility. This study aimed to investigate whether integrating home-based neuromuscular electrical stimulation (NMES) with conventional rehabilitation methods could lead to superior short-term results compared to standard care alone.

**Methods**

The study enrolled patients who underwent primary bilateral TKA at the First Medical Center of the People’s Liberation Army General Hospital between April 2023 and October 2023. Patients were randomly assigned to either the control or experimental group. The control group received a rehabilitation manual for home exercises and attended regular outpatient follow-ups for rehabilitation guidance. In contrast, the experimental group underwent a combined rehabilitation approach, incorporating NMES along with the standard regimen. Outcome measures included knee extension strength, knee joint range of motion（ROM), functional assessments (Five Times Sit-to-Stand Test (5 × SST), Single-Leg Stance Test (SLST), patient-reported outcomes (Western Ontario and McMaster Universities Osteoarthritis Index(WOMAC), Knee Society Score(KSS), The Medical Outcomes Study 36-Item Short-Form Health Survey (SF-36)), complication rates, and 90-day readmission rates.

**Results**

A total of 60 patients (30 in each group) completed the study. At the 12-week follow-up, the experimental group demonstrated statistically significant improvements over the control group in several key parameters. Specifically, the experimental group exhibited better knee joint range of motion (126.8 ± 7.6 vs. 121.1 ± 6.8, *P* = 0.005), higher physical function scores (63.4 ± 20.4 vs. 53.6 ± 19.6, *P* = 0.003), increased single-leg standing time (14.1 ± 11.8 vs 8.9 ± 6.2 seconds, *P* = 0.041), and quicker Five Times Sit-to-Stand Test (16.2 ± 3.4 vs. 19.8 ± 4.1 seconds, *P* = 0.000). However, there were no significant differences between the groups in terms of knee extension strength, WOMAC score, KSS score, 90-day readmission rates, or adverse event occurrences.

**Conclusion**

The findings of this study underscore the potential benefits of integrating NMES into the rehabilitation protocol following bilateral TKA. By complementing traditional rehabilitation methods with NMES, patients may achieve enhanced functional outcomes, as evidenced by improvements in joint range of motion and functional performance tests. The superior results observed in knee joint range of motion and functional assessments in the experimental group suggest that NMES contributes positively to postoperative recovery. The timed up-and-go test and single-leg standing time are critical measures of functional independence and balance, indicating that NMES may facilitate quicker recovery of mobility and stability post-TKA. Moreover, the SF-36 scores reflecting overall health-related quality of life were significantly higher in the experimental group, highlighting the broader impact of NMES on patient well-being beyond physical function alone. In conclusion, this study supports the incorporation of home-based NMES into the rehabilitation regimen following bilateral TKA as an adjunctive therapy. While traditional rehabilitation remains essential, the addition of NMES offers a promising avenue to optimize short-term outcomes, particularly in terms of joint mobility, functional ability, and overall quality of life. Future research could explore the long-term effects and cost-effectiveness of NMES in post-TKA rehabilitation to further validate its role in enhancing patient recovery and satisfaction.

## P18 Total knee arthroplasty: a study on tibial and femoral coronal osteotomy configuration in varus alignment

### Cheng Liang^1,2^, Xiaogang Zhang^1^, Zhongmin Jin^1^

#### ^1^Tribology Research Institute, School of Mechanical Engineering, Southwest Jiaotong University, Chengdu, China; ^2^Department of Orthopedics, The Affiliated Hospital of Southwest Medical University, Luzhou, China

##### **Correspondence:** Cheng Liang

*Arthroplasty 2024*, **6(Suppl 1):**P18

**Background**

Total knee arthroplasty is a primary treatment for end-stage knee osteoarthritis. Neutral mechanical axis alignment is commonly used in clinical practice, but approximately 20% of patients experience suboptimal outcomes. Moderate varus alignment of the knee tightens the lateral collateral ligament, enhancing knee joint stability. This moderate varus alignment benefits the restoration of knee proprioception. Both tibial and femoral coronal osteotomies can induce varus alignment. Therefore, this study primarily investigates the changes in the physical structure of the knee joint and the loading on the knee prosthesis at various degrees of flexion under different tibial and femoral coronal osteotomy configurations.

**Methods**

A knee replacement model was developed, and a numerical model was established based on varus characteristics to study the changes in the physical structure of the knee joint during flexion. Finite element analysis was used to investigate the changes in the physical structure of the knee joint and the loading on the knee prosthesis at various degrees of flexion under different varus conditions.

**Results**

During knee flexion (0° to 120°), varus alignment created solely by femoral osteotomy results in a stable varus, with the lateral collateral ligament remaining taut, which benefits knee proprioception. However, as the flexion angle increases, the knee joint’s varus moment arm lengthens, exerting greater force on the lateral collateral ligament. In contrast, varus alignment created solely by tibial osteotomy is unstable; beyond a critical angle, the varus moment reverses, causing the lateral collateral ligament to shift from being taut to becoming slack. After varus alignment, the stress on the medial side of the polyethylene insert increases, showing a positive correlation between the stress on the medial side of the polyethylene insert and the knee varus moment (Fig. 1).

**Conclusion**

Appropriate varus alignment of the knee joint can enhance stability and improve proprioception. Clinically, it is not advisable to create varus alignment solely through tibial osteotomy. Instead, varus alignment can be achieved through femoral osteotomy alone or a combination of tibial and femoral osteotomies, while avoiding the reversal of the varus moment. Using a combination of tibial and femoral osteotomies to create varus alignment can reduce the stress on the polyethylene insert compared to using femoral osteotomy alone.

**Fig. 1 (Abstract P18):**
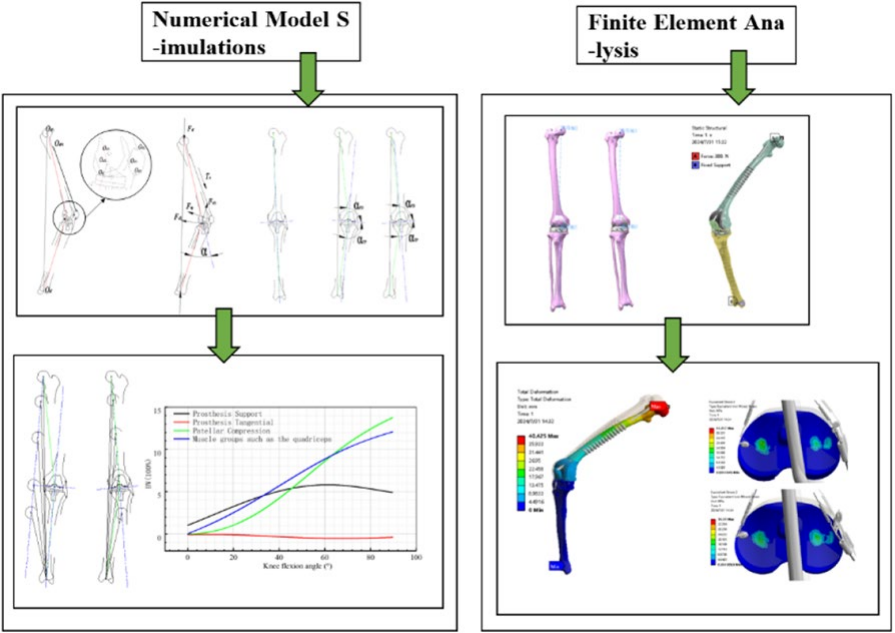
The simple flowchart

## P19 High success rate could be achieved in single-stage revision for periprosthetic joint infection: a multidisciplinary team approach

### Weijun Wang, Minghao Zhang, Yuhao Yang, Qing Jiang

#### Nanjing Drum Tower Hospital, The Affiliated Hospital of Nanjing University Medical School, Nanjing, China

##### **Correspondence:** Weijun Wang (drwilliamwang@163.com)

*Arthroplasty 2024*, **6(Suppl 1):**P19

**Background**

Single-stage revision has shown comparable clinical outcomes and reduced cost and length of hospital stay in managing periprosthetic joint infection (PJI), compared to traditional two-stage treatment. However, the single-stage approach has been used only in selected patients with strict indications in the majority centers. A multidisciplinary team (MDT) focusing on PJI treatment might facilitate the single-stage revision strategy.

**Methods**

The MDT was established including the joint surgeon, microsurgeon, microbiologist, pharmacist, rehabilitation specialist, infectious disease specialist, pathologist, and anesthesiologist, and was coordinated by the joint surgeon. The MDT would participate in the diagnosis, surgical strategy and procedure, post-operative antibiotics usage, and rehabilitation. Patients treated in our center before and after MDT conception were retrospectively reviewed, and those with a minimal one-year follow-up were recruited. The incidence of single-stage procedures, length of hospital stay (LOS), cost, and functional outcome were analyzed.

**Results**

One hundred twenty-one patients were included, including 22 consecutive cases before the MDT and 99 after the MDT. The mean follow-up duration was 53 m and 15 m, and the incidence of single-stage procedure was 31.8% (7/22) and 72.87% (72/99) in pre-MDT and post-MDT, respectively. The total LOS was reduced from 37.4 days to 28.6 days in two-stage revision, and from 23.4 days to 16.3 days in single-stage revision, and the mean LOS was 26.7 days and 18.9 days in pre-MDT and post-MDT, respectively. The total cost was reduced by MDT in both two-stage and single-stage revision, although the difference was not significant. The mean cost was significantly lower after MDT when compared to pre-MDT (US$21,035 vs US$12,983). The failure rate defined by reinfection was comparable pre- and post-MDT (4.5%, 1/22 vs 2.0%, 2/99). Functional outcomes assessed by HSS and KSS were also improved but didn’t show significant differences pre- and post-MDT.

**Conclusion**

An MDT approach involving specialists from different fields in managing patients with PJI is strongly recommended to allow individualized treatment. A high success rate could be expected by using the single-stage protocol, thereby reducing postoperative complications and failure rates.

## P20 Survivorship in robotic total knee arthroplasty compared with conventional total knee arthroplasty: a systematic review and meta-analysis

### Jiawei Chen^1^, Ryan Loke Wai Keong^1^, Katelyn Lim Kaye-Ling^1^, Barry Tan^2^

#### ^1^Yong Loo Lin School of Medicine, National University of Singapore, Singapore; ^2^Department of Orthopaedic Surgery, National University Hospital, Singapore

##### **Correspondence:** Jiawei Chen (chen.jiawei@u.nus.edu)

*Arthroplasty 2024*, **6(Suppl 1):**P20

**Background**

Total knee arthroplasty (TKA) is the gold standard surgical management for end stage knee osteoarthritis. Recently, due to enhanced accuracy and reproducibility without the need for soft tissue releases, aligning with the popularisation of functional alignment philosophy, robotic-assisted TKA (rTKA) has become very prevalent. Although its clinical outcomes are favorable compared to the conventional TKA (cTKA), due to the recency of its popularisation, there has been insufficient literature on rTKA’s effect on long-term survivorship compared to cTKA [1].

**Methods**

A random-effects meta-analysis was conducted on comparative studies between robotic-assisted TKAs and conventional TKAs in patients undergoing TKA for primary knee osteoarthritis. We searched MEDLINE, Embase, Cochrane Library, and SCOPUS from inception to 30 July 2024. Pooled proportions were obtained with random-effects modeling, 95% confidence intervals estimated using the Clopper-Pearson method, and the Dersimonian-and-Laird estimator for between-study variance.

**Results**

14 comparative studies were included in the meta-analysis. A total of 2641 knees underwent cTKA while 2314 underwent rTKA. Demographics were pooled for age, BMI, and surgery duration. Studies were subdivided into short-term (≤5 years) and long-term (≥10 years) follow-up. Regarding our primary outcome, the difference in short-term survivorship rates between cTKA (96.49% [95% CI: 94.45–97.80]) and rTKA (98.08% [95% CI: 95.63–99.17]) was insignificant (*P* = 0.21). Differences in long-term survivorship rates for cTKA (95.98% [95% CI: 92.94–97.75]) and rTKA (97.33% [95% CI: 95.91–98.26]) were also insignificant (*P* = 0.27). Regarding our secondary outcome, the difference between total complication rates for cTKA (4.97% [95% CI: 2.74–8.83]) and rTKA (3.89% [95% CI: 2.15–6.96]) was also insignificant (*P* = 0.57) (Figs 1 and 2).

**Conclusion**

cTKA is non-inferior to rTKA at short-term and long-term follow-ups with regards to implant survival, revision, and complications.

**Reference**

1. Zhang J et al. 2022. Robotic-arm assisted total knee arthroplasty is associated with improved accuracy and patient reported outcomes: a systematic review and meta-analysis [published correction appears in Knee Surg Sports Traumatol Arthrosc. 2022 Aug;30(8):2696–2697.

**Fig. 1 (Abstract P20):**
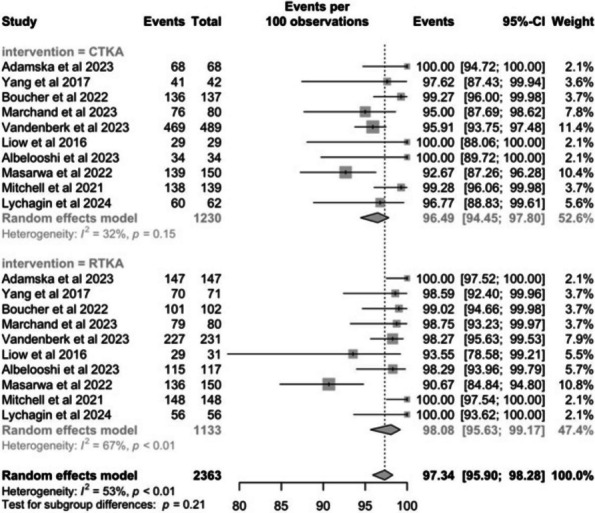
Short-term (≤5 years) survivorship

**Fig. 2 (Abstract P20):**
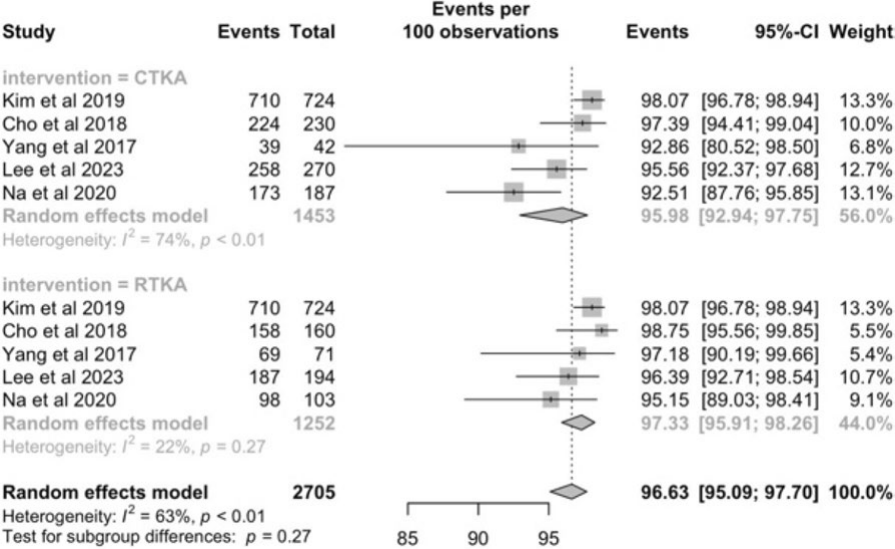
Long-term (≥10 years) survivorship

## P21 Cardiac function during tourniquet application in simultaneous bilateral knee arthroplasty: an orthopaedic perspective on ejection fraction

### Kittitat Jongjarukawin, Pat Laupattarakasem, Praew Kotruchin, Weerachai Kowsuwon, Kamolsak Sukonthaman

#### Department of Orthopaedics, Khon Kaen University, Khon Kaen, Thailand

##### **Correspondence:** Kittitat Jongjarukawin (kelvinzw9429@gmail.com)

*Arthroplasty 2024*, **6(Suppl 1):**P21

**Background**

The use of tourniquets during bilateral knee arthroplasty is a common practice aimed at reducing intraoperative blood loss and improving the surgical field. However, the impact of tourniquet application on cardiac function, particularly on the ejection fraction (EF), remains a concern, especially in patients with pre-existing cardiac conditions. This study aims to evaluate the changes in ejection fraction (EF) at baseline and during the tourniquet phase in patients undergoing bilateral knee arthroplasty and to understand the potential clinical implications of these changes [1–12].

**Methods**

A cohort of patients scheduled for bilateral knee arthroplasty was assessed using echocardiography to measure EF at baseline and during the tourniquet phase. The primary outcome was the change in EF, and secondary outcomes included other echocardiographic parameters such as E/E’ ratio, left ventricular internal dimensions, and pulmonary capillary wedge pressure (PCWP) (Table 1).

**Results**

The results show that the mean EF during the tourniquet phase (Eftour) was 0.70 ± 0.09, compared to the baseline EF (Efbase) of 0.75 ± 0.07. This decrease in EF during the tourniquet phase suggests a temporary impairment of cardiac function, which maybe attributed to the increased afterload and reduced venous return associated with the tourniquet application. The results for both base-tourniquet and post-tourniquet EF are not significantly different which means the patients are safe on bilateral tourniquet in simultaneous bilateral total knee arthroplasty (Fig. 1).

**Conclusion**

This research provides valuable insights into the cardiac implications of tourniquet use during bilateral knee arthroplasty, emphasizing the need for careful monitoring and management of ejection fraction to ensure optimal patient outcomes. The result is ensuring that the patients are safe during bilateral tourniquet in simultaneous bilateral total knee arthroplasty.

**References**

1. Luscombe JC, Theivendran K, Abudu A, Carter SR. The relative safety of one- stage bilateral total knee arthroplasty. Int Orth (SICOT) 2009;33:101-4
2. Memtsoudis SG, Besculides MC, Reid S, Gaber LK, Gonzalez Della Valle A. Trends in bilateral total knee arthroplasties: 153,259 discharges between 1990 and 2004. Clin Orthop Relat Res;467:1568-76
3. Cohen GR, Forrest JC, Benjamin JB. Safety and efficacy of bilateral total knee arthroplasty. J Arthroplasty 1997;12:497–501
4. Huang GS, Wang CC, Hu MH, Cherng CH, et al. Bilateral passive leg raising attenuates and delays tourniquet deflation-induced hypotension and tachycardia under spinal anaesthesia. Eur J Anaesthesiol 2014; 31:15–22
5. Memtsoudis SG, Ma Y, Chiu Y, Poultsides L, Gonzalez Della Valle A. Bilateral total knee arthroplasty: risk factors for major morbidity and mortality. Anesth Analg 2011;113:784-90
6. Powell RS, Pulido P, Tuason MS, Colwell CW, Ezzet KA. Bilateral vs unilateral total knee arthroplasty: a patient-based comparison of pain levels and recovery of ambulatory skills. J Arthroplasty 2006;21:642-8
7. Girardis M, Milesi S, Donato S, Raffaelli M, Spasiano A, Antonutto G, Pasqualucci A, Pasetto A. The hemodynamic and metabolic effects of tourniquet application during knee surgery. Anesth Analg 2000;91:727-31
8. Cohen RG, Forrest CJ, Benjamin JB. Safety and efficacy of bilateral total knee arthroplasty. J Arthroplasty 1997;12:497–502
9. MacMillan DP, Larson CM, Lachiewicz PF. Thromboembolism after total Knee Arthroplasty: The role of pneumatic compression and aspirin prophylaxis. The Journal of Arthroplasty. 1998;13(2):241. 10.1016/s0883-5403(98)90162-0.
10. Enns P, Garceau S, Teo G, Pollock S, Long WJ. Comparing sequential vs simultaneous tourniquet inflation in bilateral total knee arthroplasty. Arthroplasty Today. 2021;8:132-7. 10.1016/j.artd.2021.02.005
11. Liu P, Li D, Zhang Y, Lu Q, Ma L, Bao X, et al. Effects of unilateral tourniquet used in patients undergoing simultaneous bilateral total knee arthroplasty. Orthopaedic Surgery. 2017;9(2):180-5. 10.1111/os.12329
12. Stanley D, Stockley I, Getty C. Simultaneous or staged bilateral total knee replacements in rheumatoid arthritis. A prospective study. The Journal of Bone and Joint Surgery British volume. 1990;72-B(5):772-4. 10.1302/0301-620x.72b5.2211753

**Table 1 (Abstract P21):** Patient characteristics

Characteristic	Mean ± SD
Age (years)	67.17 ± 7.82
Gender (Male/Female)	17/1
Height (cm)	154.00 ± 5.85
Weight (kg)	64.55 ± 13.56
BMI (kg/m²)	26.98 ± 5.79
BSA (m²)	1.63 ± 0.17

**Fig. 1 (Abstract P21):**
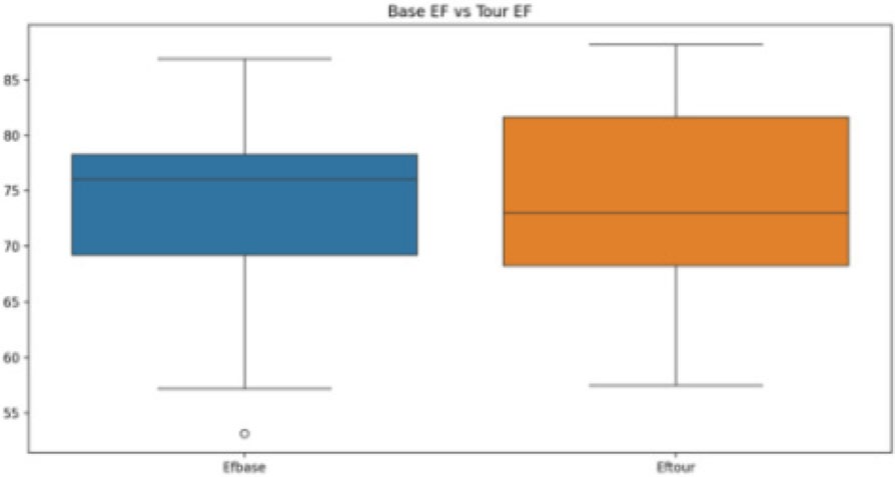
Study results

## P22 Effect of central sensitization state on the minimal clinically important differences of the patient-reported outcome measurement after total knee arthroplasty

### Shuxin Yao, Chao Xu, Jianbing Ma

#### Department of Knee Joint Surgery, Honghui Hospital, Xi’an Jiaotong University, Xi’an, China

##### **Correspondence:** Jianbing Ma (drmajianbing@163.com)

*Arthroplasty 2024*, **6(Suppl 1):**P22

**Objective:** Central sensitization (CS) is a significant risk factor for pain and dissatisfaction following total knee arthroplasty (TKA). Currently, the impact of CS status on the minimal clinically important differences (MCID) of patient-reported outcome scales after TKA is still unclear. This study aims to explore the effect of central sensitization status on the MCID of the Brief Pain Inventory (BPI).

**Methods:** A total of 296 patients with knee osteoarthritis who underwent unilateral TKA were included in this study and completed a 1-year follow-up. The Central Sensitization Inventory (CSI) was used to assess the CS status of the patients, and the BPI scale was used to assess the pain and functional disability of the patients. The MCID of the BPI score in the CS group and the non-CS group was calculated using the anchoring method and the distribution method, respectively. The change difference method was used in the anchoring method to define the MCID as the difference between the preoperative and postoperative changes in the BPI score between the minimal improvement group and the no change group. Additionally, the proportion of patients in the CS group and the non-CS group who achieved the MCID after surgery was compared.

**Results:** According to the change difference method, the MCIDs of the total score, pain dimension, and pain interference dimension of the BPI in the CS group were 11.1 points, 4.8 points, and 6.2 points, respectively. The MCIDs of the total score, pain dimension, and pain interference dimension of the BPI in the non-CS group were 7.9 points, 3.8 points, and 4.1 points, respectively. The proportion of patients in the CS group who achieved the MCID in each dimension and the total score of the BPI was significantly lower than that in the non-CS group (*P* < 0.05).

**Conclusion:** CS status has a substantial impact on the MCID of the BPI score after TKA. The MCID of the BPI score in the CS group was higher, and it was more difficult for them to achieve the MCID after surgery. This finding may partially explain the low satisfaction of CS patients after TKA. 

## P23 Microorganism patterns in prosthetic joint infection from a tertiary hospital in Malaysia

### Muhindra Rao, Muhammad Azhar Bin Abdullah, Ahmad Fauzey Bin Kassim, Mohd Aizat Azfar Bin Soldin

#### Orthopaedic Department, Hospital Sultanah Bahiyah, Alor Setar, Kedah, Malaysia

##### **Correspondence:** Muhindra Rao (muhindra86@gmail.com)

*Arthroplasty 2024*, **6(Suppl 1):**P23

**Background and Aims**

Prosthetic joint infections (PJIs) represent a significant and devastating complication following arthroplasty, often leading to increased morbidity and healthcare costs. Understanding the microbiological profile of PJIs is crucial for improving treatment strategies [1, 2].

**Methods**

A retrospective study was conducted in a tertiary centre in Malaysia on patients that were readmitted due to prosthetic joint infection from 2022 to 2024. Microbiological cultures collected from the tissue of the infected joint either during surgery or via aspirations were analyzed to obtain the predominant pathogens.

**Results**

A total of 80 patients were included in this study. The most common pathogen is Staphylococcus aureus, found in 22 (28%) cases. Coagulase-negative staphylococci followed as the second most common pathogen, accounting for (20%) of infections. The other microorganisms that were found are listed in Table 1.

**Conclusion**

The predominance of Staphylococcus aureus and coagulase-negative staphylococci highlights the necessity for targeted antibiotic therapy and effective preventive strategies in managing prosthetic joint infections. This is also paramount to prevent the development of resistance in Staphylococcus aureus. Future research should investigate the implications of these findings on treatment protocols and the development of resistance patterns in the context of prosthetic joint infection management.

**References**

1. Tande AJ, Patel R. Prosthetic joint infection. *Clin Microbiol Rev.* 2014;27:302–45
2. Parvizi J, Zmistowski B, Berbari EF, Bauer TW, Springer BD, Della Valle CJ, et al.

**Table 1 (Abstract P23):** Study results

Microorganism	Percentage of total infections (%)	Number of cases
Staphylococcus aerus	28	22
coagulase negative staphylococcus	20	16
Pseudomonas aeroginasa	5	4
Streptococcus anginosus	8	6
MSSA	10	8
MRSA	10	8
Enterococcus faecalis	3	2
ESBL	3	2
Acinetobacter Baumannii	3	2
Klebsiella Pneumonia	3	2

## P24 Readmission rates and reasons in patient post total joint replacement in tertiary hospital

### Muhindra Rao, Muhammad Azhar Bin Abdullah, Ahmad Fauzey Bin Kassim, Mohd Aizat Azfar Bin Soldin

#### Orthopaedic Department, Hospital Sultanah Bahiyah, Alor Setar, Kedah, Malaysia

##### **Correspondence:** Muhindra Rao (muhindra86@gmail.com)

*Arthroplasty 2024*, **6(Suppl 1):**P24

**Background and Aims**

Total joint arthroplasty (TJA) is a common surgical intervention that significantly improves the quality of life for patients with joint disorders. Despite being a relatively successful surgery, patients do encounter complication which necessitates readmission to the hospital after the index surgery. Readmission types should be categorized to identify the reasons and take necessary precautions to reduce readmission rates. This study aims to analyse the factors contributing to readmissions after total joint arthroplasty and to identify strategies for reducing these occurrences.

**Methods**

A comprehensive review of patient records was conducted, focusing on individuals who underwent total joint replacement over four years. Data collected included demographic information, comorbidities, surgical details, postoperative complications, and readmission rates. Statistical analyses were performed to identify significant predictors of readmission. 

**Results**

This is a retrospective study done in a tertiary centre in Malaysia for 4 years (2021–2024). This study includes a total of 730 patients who underwent total joint arthroplasty. Among these patients, a total of 46 patients (6.3%) were readmitted due to several causes. The leading etiology for readmission is periprosthetic fracture/dislocation which includes 43% of the cases that were readmitted. This is followed by surgical site infections/septic arthritis of the joint (33%). Other causes of readmission are tabulated in Table 1.

**Conclusion**

Understanding the determinants of readmission after total joint arthroplasty is essential for improving postoperative care and patient education. Targeted interventions, including preoperative risk assessment and enhanced recovery protocols, may help reduce readmission rates and optimize patient outcomes. Further research is needed to develop evidence-based strategies for managing high-risk populations after total joint replacement.

**References**

1. Alexander J. Metoxen, MD Hospital Readmissions After Total Joint Arthroplasty: An Updated Analysis and Implications for Value-Based Care, The Journal of Arthroplasty, Volume 38, Issue 3, p431-436, March 2023
2. Courtney, P.M. Boniello, A.J. Berger, R.A. Complications following outpatient total joint arthroplasty: an analysis of a national database. *J* *Arthroplasty.* 2017; 32:1426–1430

**Table 1 (Abstract P24):** Study results

Factors of readmission	Percentage (%)
Periprosthetic fracture/dislocation	33
Surgical site infections/septic arthritis of the joint	43
Venous thromboembolism	6.5
Joint stiffness	8.7
Pain	8.7

## P25 Double crush: valgus knee and tibial bowing in primary total knee arthroplasty

### Kar Wai Loh, Rui Lin Ki, Ramachandran Rubenandran

#### Department of Orthopaedic and Traumatology, Sungai Buloh Hospital, Sungai Buloh, Selangor, Malaysia

##### **Correspondence:** Kar Wai Loh (drlohkarwai@gmail.com)

*Arthroplasty 2024*, **6(Suppl 1):**P25

**Introduction**

Valgus knee is technically challenging in the hands of low-volume surgeons in performing total knee arthroplasty (TKA). This condition made worse with the presence of medial tibial bowing where tibial component placement following the mechanical axis of the tibia could worsen the valgus knee. Careful preoperative planning is mandatory to prevent component malalignment. 

**Case Report**

A 70-year-old lady with left TKA done 8 years prior was planned for right total knee arthroplasty for right knee osteoarthritis. She has severe right knee valgus with Hip Knee Ankle Angle (HKAA) of 191 degrees and medial tibial bowing of 9 degrees, otherwise, her knee ligaments were intact. Right TKA was performed with semi-constrained posterior stabilized knee arthroplasty. The tibial cut was performed with an extramedullary tibial jig placed medial to the tibial spine following the mechanical axis centered at the ankle joint (Figs.1 and 2). 6 weeks post-operatively she had a 70% reduction in pain score and was ambulating with a stable right knee. The patient gave their informed written consent to publish their information in an open access journal.

**Discussion**

35% of valgus knee had tibia valga and there are positively correlated [1]. Long limb radiographs should be performed for pre-operative planning. A medialized point for extramedullary tibial jig placement was recommended for tibial bowing more than 3 degrees to restore limb alignment [2].

Conclusion

Tibial bowing needs to be taken into consideration in cases of valgus knee in performing TKA successfully. 

**References**

1. Sobhi, S., Khan, R.J.K., Fick, D.P. et al. Prevalence of extra-articular tibia valga morphology in valgus knees and its implications for primary total knee arthroplasty. *J Orthop Surg Res* 17, 531 (2022).
2. Palanisami, Dhanasekararaja, et al. Tibial bowing and tibial component placement in primary total knee arthroplasty in valgus knees: are we overlooking? *Journal of Orthopaedic Surgery* 27.3 (2019).

**Fig. 1 (Abstract P25):**
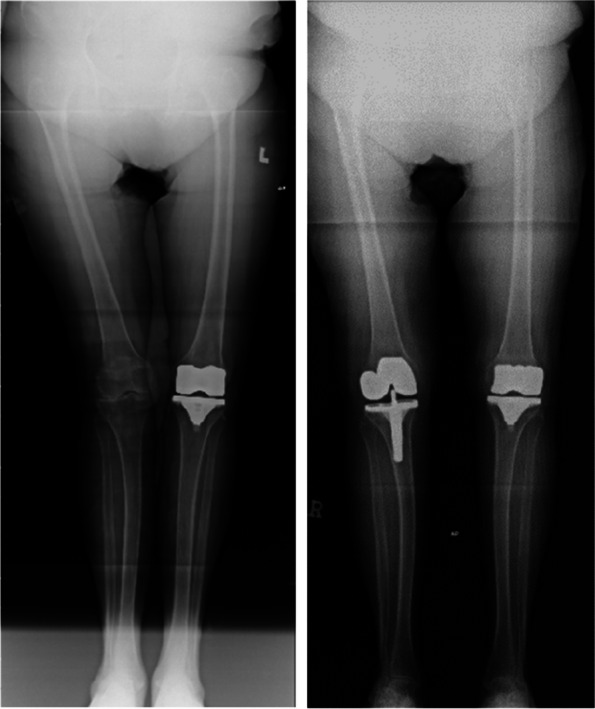
Pre- and post-operative long limb axis view

**Fig. 2 (Abstract P25):**
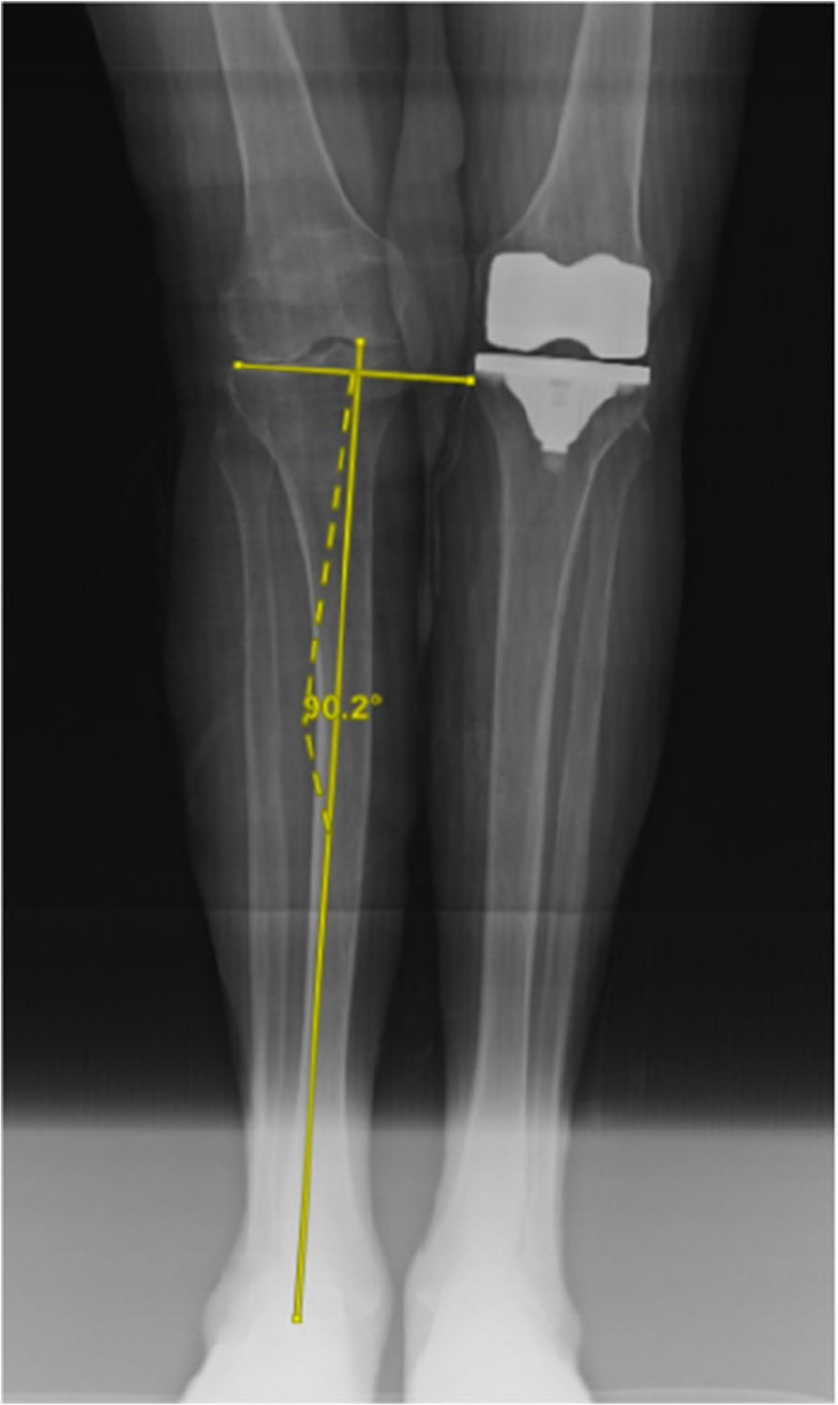
Tibial jig placement medial to the tibial spine centered at the ankle joint

## P26 Comparative study of early recovery and clinical outcome between internal fixation and direct anterior approach hip arthroplasty in elderly femoral neck fractures

### Win Bhudhavudhikrai, Sarit Hongvilai, Wallob Samranvedhya, Sombat Rojviroj, Boonyarak Visutipol, Phonthakorn Panichkul, Panuwat Silawatshananai, Natapha Kiriyapong

#### Hip and Knee Center, Bangkok International Hospital, Bangkok, Thailand

##### **Correspondence:** Win Bhudhavudhikrai (bhudhavudhikrai.win@gmail.com)

*Arthroplasty 2024*, **6(Suppl 1):**P26

**Background**

Femoral neck fractures (FNF) in the elderly population present a significant clinical challenge due to prevalent comorbidities and the inherent fragility of the bone. Two principal surgical interventions, internal fixation (IF) and hip arthroplasty, are commonly employed, each offering distinct benefits and limitations. The direct anterior approach (DAA) in hip arthroplasty, noted for its minimally invasive techniques, has shown potential advantages over traditional surgical approaches. This study aims to compare early recovery outcomes between IF and DAA hip arthroplasty in patients with fragility femoral neck fractures.

**Methods**

A retrospective chart review from 2016–2022 was conducted involving patients aged 50 years and older with FNF. Exclusion criteria included non-ambulatory status, delayed presentation to treatment, and fractures resulting from pathologies other than osteoporosis. The IF group was matched with the DAA hip arthroplasty group in a 1:4 ratio. Descriptive statistics and relevant statistical tests were utilized to compare baseline characteristics and outcomes.

**Results**

The study included 120 patients with femoral neck fractures, comprising 24 cases of IF and 96 cases of DAA hip arthroplasty. The majority of IF procedures employed the Femoral Neck System (FNS) (16/24, 66.7%), while DAA hip arthroplasty predominantly involved partial hip replacement (88/96, 91.7%) and cementless fixation (74/96, 77.1%). Patients undergoing DAA hip arthroplasty were significantly older (77.6 ± 8.4 vs. 70.3 ± 13.9 years, *P* = 0.001), exhibited higher rates of fracture displacement (87.5% vs. 33.3%, *P* < 0.001), and reported more pain (3.3 ± 1.0 vs. 2.7 ± 1.3, *P* = 0.014) preoperatively compared to those undergoing IF. Additionally, DAA hip arthroplasty patients were able to ambulate earlier (1.6 ± 0.8 days vs. 2.5 ± 3.1 days, *P* = 0.011) and achieved better ambulatory status at discharge. However, the IF group experienced less intraoperative blood loss (89.0 ± 79.0 mL vs. 313.8 ± 237.3 mL, *P* < 0.001) and shorter hospital stays (5.5 vs. 7 days, *P* < 0.001). There were two intraoperative calcar fractures (2.1%) in the DAA hip arthroplasty group.

**Conclusions**

Patients with FNF who underwent DAA hip arthroplasty demonstrated earlier recovery compared to those who underwent IF, without an increased risk of complications relative to traditional approaches. Thus, DAA hip arthroplasty is a viable, safe, and effective option for facilitating early recovery in patients with fragility femoral neck fractures.

## P27 Comparative analysis of hemi-hip replacement outcomes: a study on femoral neck fractures versus intertrochanteric fractures

### Somchai Taosuwan, Varah Yuenyongviwat

#### Department of Orthopedic, Faculty of Medicine, Prince of Songkla University, Songkhla, Thailand

##### **Correspondence:** Somchai Taosuwan (maximtao2@gmail.com)

*Arthroplasty 2024*, **6(Suppl 1):**P27

**Background**

Bipolar Hemiarthroplasty is the standard treatment for both femoral neck and intertrochanteric fractures in elderly patients. There are no comparative studies of bipolar hemiarthroplasty for femoral neck fractures and intertrochanteric fractures with a posterior approach in the same treatment setting procedure. Therefore, this study is conducted to obtain results that will guide patient preparation and provide advice to patients on making informed decisions.

**Objective**

The purpose of this study is to compare the primary outcomes of bipolar hemiarthroplasty in two different types of hip fractures within the same approach and sector.

**Methods**

A retrospective study was conducted based on collected medical records from Songklanagarind Hospital between 2013 to 2023. The study included two groups: 38 patients with intertrochanteric fractures and 152 patients with femoral neck fractures, all aged over 60 years, who underwent bipolar hemiarthroplasty. Data were collected to compare primary outcomes, including operative time, hospital stay, blood loss, complications, and ambulation between the two groups.

**Results**

Operating time was longer for intertrochanteric fractures (195 compared to 170 minutes). intertrochanteric fractures group underwent more complex procedures with wire, resulting in greater blood loss and more postoperative transfusions. Intraoperative fractures, particularly calcar fractures, were more common in the femoral neck group. The intertrochanteric group had a longer hospital stay (16.3 compared to 9.8 days) due to postoperative complications. Both groups experienced delayed ambulation.

**Conclusion**

This research suggests that bipolar hemiarthroplasty for intertrochanteric fractures requires more preparation compared to femoral neck fractures. It involves the use of wire, blood transfusion units, specialized operative teams, and prolonged hospitalization. Hence, hemiarthroplasty for intertrochanteric fractures should be performed by specialists in hip surgery.

## P28 Femoral geometry-based model for precision sizing of femoral stems in hip arthroplasty

### Kritsada Sukha, Wiboon Wanitcharoenporn, Burin Sutthapakti, Witoon Thremthakanpon

#### Department of Orthopedic, Buddhachinaraj Hospital, Phitsanulok, Thailand

##### **Correspondence:** Kritsada Sukha (kritsada.sukha@gmail.com)

*Arthroplasty 2024*, **6(Suppl 1):**P28

**Background**

Precision sizing of femoral stems in hip arthroplasty is crucial for optimal patient outcomes. Traditionally, stem size selection has relied heavily on surgeon experience and preoperative imaging. This study aims to develop a femoral geometry-based model for precision sizing of femoral stems in hip arthroplasty, integrating both demographic data and preoperative parameters, particularly the P20/D20 ratio and Canal Bone Ratio (CBR).

**Methods**

A retrospective analysis was conducted on 750 patients who underwent hip arthroplasty with uncemented Corail stem. The patient cohort comprised 60.5% females with a mean age of 64.19 ± 14.93 years, mean weight of 56.29 ± 11.75 kg, mean height of 1.59 ± 0.08 m, and mean BMI of 22.25 ± 4.14 kg/m². Most patients had femoral neck fractures (56.5%) and underwent total hip arthroplasty (57.6%). Key measurements included P20/D20 ratio and CBR, with most patients classified as Dorr type B (74.1%).

**Results**

The average stem size usage was 10.69 ± 1.50. Correlation analysis revealed significant relationships between stem size usage and body weight (*r* = 0.196, *P* < 0.001), height (*r* = −0.399, *P* < 0.001), P20/D20 (*r* = −0.492, *P* < 0.001), and CBR (*r* = 0.423, *P* < 0.001). Multivariable analysis considering only demographic factors (sex, age, body weight, and height) explained 21.1% of the variance in stem size usage. Including preoperative parameters (P20/D20 ratio and CBR) increased the explained variance to 27.2%. The final

model, incorporating both demographic and preoperative parameters, explained 44.3% of the variance in stem size usage. Significant predictors included sex, age, body weight, height, P20/D20 ratio, and CBR. The regression equation developed was Stem size usage = 0.640 + 0.508 × (Male = 1, Female = 0) + 0.022 × Age (years) + 0.014 × Weight (kg) + 5.047 × Height (m) − 1.411 × P20/D20 + 6.216 × CBR. Using preoperative parameters alone, the model achieved an accuracy of 88.3% in predicting the correct stem size within ±1 size. However, incorporating both demographic and preoperative parameters increased the accuracy to 92.3%. Consequently, the model that includes both sets of parameters was selected as the predictive model due to its highest accuracy.

**Conclusion**

This study highlights the significant influence of both demographic and preoperative parameters on stem size usage in hip arthroplasty, with a particular focus on the P20/D20 ratio and CBR. The developed model provides a robust framework for precision sizing of femoral stems in clinical practice, promising improved outcomes and standardization in hip arthroplasty procedures. To facilitate the implementation, a free website for calculating the femoral stem size was developed.

## P29 Comparison of patient satisfaction following conventional vs robotic-assisted bilateral TKA

### Swist Chatmaitri, Chaturong Pornrattanamaneewong, Keerati Chareancholvanich, Rapeepat Narkbunnam

#### Department of Orthopaedic Surgery, Faculty of Medicine, Siriraj Hospital, Mahidol University, Bangkok, Thailand

##### **Correspondence:** Swist Chatmaitri (swist_swist@hotmail.com)

*Arthroplasty 2024*, **6(Suppl 1):**P29

**Purpose**

To compare the satisfaction rate at 1 year between bilateral robotic-assisted total knee arthroplasty and conventional total knee arthroplasty.

**Material and methods**

A prospective data was collected from 270 cases (90 robotic-assisted and 180 conventional TKA) in Siriraj Hospital between 2021–2023. Baseline characteristics were recorded. The primary outcome was the overall satisfaction rate at 1 year postoperative. Secondary outcomes include the 2011 Knee Society Scoring System, maximum NRS Pain score (at rest, during ambulation), and EQ5D.

**Results**

84.7% of patients in the robotic-assisted group were very satisfied compared to 72% in the conventional group. However, the results were not statistically significantly different. For secondary outcomes, only differences in pain scores at rest were found. The patients in the robotic-assisted group demonstrated lower pain scores at rest compared to the robotic-assisted group. Other secondary outcomes were not found to be different. 

**Conclusion**

Both bilateral robotic-assisted and conventional TKA provide good and comparable satisfaction rates.

## P30 Whole-organ magnetic resonance imaging score does not correlate with clinical improvement following platelet-rich plasma injections in knee osteoarthritis

### Chayanin Lertmahandpueti, Nonn Jaruthien, Chotetawan Tanavalee, Chavarin Amarase, Srihatach Ngarmukos, Aree Tanavalee

#### Department of Orthopedic, Faculty of Medicine, Chulalongkorn University, Bangkok, Thailand

##### **Correspondence:** Chayanin Lertmahandpueti (chayanin.lert@gmail.com)

*Arthroplasty 2024*, **6(Suppl 1):**P30

**Introduction**

This observational prospective cohort study evaluates the effectiveness of three courses of platelet-rich plasma (PRP) injections in patients with knee osteoarthritis (OA). The semi-quantitative whole-organ scoring system, Whole-organ magnetic resonance imaging score (WORMS), was used to assess structural changes in the knee post-PRP injection over 6 months. 

**Methods**

Twenty OA knee patients receiving PRP injections as an alternative treatment were graded pre- and post-injection using the WORMS scoring system. Clinical outcomes, including visual analog scale (VAS), patient-reported outcome measures (PROMs) such as the Western Ontario and McMaster Universities Osteoarthritis (WOMAC) Index, and performance-based measures (PBMs) including time up and go (TUG), 5-time sit to stand test (5 × SST), and 3-minute walk test (3-min WT), were also recorded. 

**Results**

The overall WORMS increased from 38.15 ± 17.48 (pre-treatment) to 40.30 ± 18.87 (post-treatment), with a mean change of 2.15 (95% CI: 0.82, 3.48). Specifically, the WORMS for articular cartilage showed the most increase, with a mean change of 1.45 (95% CI: 0.5, 2.4). Significant improvement in VAS was observed at 3 weeks post-treatment, with both clinical and statistical significance observed at 6 weeks and the final follow-up at 6 months. Delayed improvement in PBMs, at 6 months, was also noted. 

**Conclusion**

Clinical improvement following a course of 3-PRP injection in knee OA does not correlate with WORMS after 6 months. Changes of WORMS within grade or even worsening of them were observed, while improvement in VAS, PROMs, and PBMs were also noted.

## P31 Is CORI system accurate in sizing for knee arthroplasty?

### Khanaphong Pornsiriwattanakun, Nonn Jaruthien, Chotetawan Tanavalee, Chavarin Amarase, Srihatach Ngarmukos, Aree Tanavalee

#### Department of Orthopedic, Faculty of Medicine, Chulalongkorn University, Bangkok, Thailand 

##### **Correspondence:** Khanaphong Pornsiriwattanakun (bossoone@gmail.com)

*Arthroplasty 2024*, **6(Suppl 1):**P31

**Introduction**

The use of robotic assistance in total knee arthroplasty (R-TKA) has demonstrated reliable outcomes in bone cutting and implant positioning, potentially reducing post-operative patient dissatisfaction. The CORI system (Smith & Nephew, London, Great Britain) employs a morphing technique to delineate bone boundaries. This study aimed to investigate the accuracy of implant sizing using the CORI system. 

**Method**

A retrospective study was conducted on 26 patients undergoing primary R-TKA with the CORI system in our department. Preoperative planning utilized 2D digital templates. Predicted component sizes from both the CORI system and OrthoView™ were compared to the actual implantation to assess accuracy. 

**Result**

The morphing system accurately predicted the size of femoral components in 42.3% of cases and tibial components in 26.9%. The Intraclass Correlation Coefficient (ICC) values were 0.2571 for femoral components and 0.0852 for tibial components. Considering errors within one size of the implanted size, the morphing system's accuracy increased to 96.2% for femoral components and 76.9% for tibial components. Preoperative 2D templating accurately predicted the correct size of femoral components in 57.7% of cases and tibial components in 46.2%. The ICC values for 2D templating were 0.4381 for femoral components and 0.1875 for tibial components. When considering errors within one size, 2D templating accurately predicted sizes in 92.3% of femoral components and 84.6% of tibial components. 

**Conclusion**

Overall, preoperative 2D templating demonstrated higher accuracy than the morphing system in predicting implant sizes. The ICC values for femoral components were 0.4381 (2D templating) versus 0.2571 (morphing), with a non-significant *P*-value of 0.320. For tibial components, the ICC values were 0.1875 (2D templating) versus 0.0852 (morphing), with a non-significant *P*-value of 0.410.

## P32 Comparison of high flexion activity after total knee replacement between anatomical and non-anatomical implant

### Tanat Siripoon, Nattapol Tammachote

#### Department of Orthopedics, Faculty of medicine, Thammasat University, Bangkok, Thailand

##### **Correspondence:** Tanat Siripoon (tanat.srp@gmail.com)

*Arthroplasty 2024*, **6(Suppl 1):**P32

**Background**

High flexion activity after total knee replacement remains crucial in Asians regarding cultural and religious activities. This study aims to determine if a newer, anatomical design of a total knee replacement (Journey II BCS) provides superior outcomes in terms of high flexion activity than the older non-anatomical designs (Legion TKS).

**Method**

This single-centered, comparative retrospective review includes a total of 145 patients undergoing primary TKA with a follow-up period of at least one year post-operatively. 84 patients undergoing TKA with anatomical Journey II BCS designs group and 61 patients undergoing non-anatomical Legion TKS designs group were recruited. The outcome was a post-operative high flexion knee (HFKS) score compared at a minimum of 1 year post-operatively.

**Results**

At the mean follow-up of 52 ± 25 months, the mean post-operative high flexion knee scores were similar between Journey II and Legion TKS group. (13.08 ± 1.42 vs. 13.18 ± 1.37, *P* = 0.68) The mean increase in post-operative knee flexion was similar between Journey II and Legion TKS group. (19 ± 8.0 vs. 18 ± 7.3, *P* = 0.58) The mean modified Thai WOMAC scores were also similar. (4.6 ± 1.72 vs. 4.72 ± 1.61)

**Conclusion**

This study demonstrated that newer anatomical Journey II designs have no effect on post-operative high flexion knee scores, post-operative knee flexion angle, and modified Thai WOMAC scores compared to non-anatomical Legion TKS designs. Other factors to obtain optimal high flexion knee activities can be further investigated.

## P33 Are preoperative BUN-to-Creatinine ratios associated with increased postoperative short-term complications in patients undergoing primary total knee arthroplasty?

### Natthasit Wongwarawanith, Piya Pinsornsak

#### Department of Orthopaedics, Faculty of Medicine, Thammasat University, Bangkok, Thailand

##### **Correspondence:** Natthasit Wongwarawanith (natthasit_fight@hotmail.com)

*Arthroplasty 2024*, **6(Suppl 1):**P33

**Background**

From previous studies, preoperative dehydration was known to affect the outcomes and complications after other several operations. This study aimed to evaluate the association between preoperative hydration status, using values of BUN/Cr ratio, and post-operative complications after primary total knee arthroplasty.

**Materials and methods**

All patients undergone primary TKA between 2018–2023 were retrospectively reviewed. Patients were then classified into 2 groups: BUN/Cr > 20 and BUN/Cr ≤ 20. 90-day complications, readmissions, and mortality rates were compared between groups. Multivariable analyses were also conducted to eliminate potential confounding factors.

**Results**

The analysis included 363 TKA patients, 42.42% of which (154 patients) had preoperative BUN/Cr ratio > 20. After multivariable analyses, patients with preoperative BUN/Cr ratio > 20 had greater odds of overall 90-day complications (OR 3.04, CI 1.52–6.09, *P* = 0.002). Patients in this group were also associated with greater length of stay (MD 0.72, CI 0.54–0.9, *P* < 0.001) and positive fluid balance at day 1 (MD 477, CI 293–661, *P* < 0.001).

**Conclusion**

Patients with preoperative BUN/Cr > 20 were associated with a greater risk of 90-day complications after primary TKA. Surgeons should be aware of the importance of these laboratory values to prevent complications after surgery.

## P34 Investigation of the impact of dexamethasone for pain control after bilateral total knee arthroplasty in police general hospital: randomized control trial

### Kanok Pawanja, Ukrit Chaweewannakorn, Viroj Larbpaiboonpong

#### Department of Orthopedic Surgery, Police General Hospital, Bangkok, Thailand

##### **Correspondence:** Kanok Pawanja (kanat.ah34@gmail.com)

*Arthroplasty 2024*, **6(Suppl 1):**P34

**Backgrounds**

Total knee arthroplasty (TKA) is improving quality of life and restoring patients to a higher level of function in the end stage of osteoarthritis patients. However, TKA is moderate to severe postoperative pain and has more inflammation biomarkers. Management of pain in the immediate postoperative period is important to allow speedier rehabilitation and reduce the risk of postoperative complications. Dexamethasone is a long-acting glucocorticoid, a powerful anti-inflammatory, and decreases pain-activated products from the cyclooxygenase and lipoxygenase pathway.

**Purpose**

To compare pain score after bilateral TKA between the dexamethasone group and control group at 24, 48, 72, and 96 hours.

**Study Design**

Randomized control trial.

**Methods**

A total of 42 patients with bilateral OA knee were randomized into 2 groups. Dexamethasone (DEX) and No dexamethasone (NOD). The 21 patients in each group. All were spinal anesthesia. They underwent simultaneous bilateral TKA with subvastus approach. NO multimodal periarticular injection or Local infiltration technique. Immediately Dexamethasone 16 mg. intravenous at the recovery room in the Dexamethasone (DEX) group. We recorded Pain score (visual analog score 0–10), Pethidine consumption (mg/day) CRP, Blood sugar level, and other complications.

**Results**

The 42 patients were divided into two groups, 21 patients in each group were included and excluded. There is no difference in demographic data. The DEX group had significantly statistically reduced pain scores during motion in 48, 72, and 96 hours. post-op better than when compared to NOD, but no difference significantly in clinical outcome. No significant difference in pethidine consumption in both groups. There were significantly reduced inflammation biomarkers (CRP) in the DEX group.

**Conclusions**

The DEX group had significantly reduced pain scores and reduced inflammation biomarkers (CRP) when compared NOD group. Dex group also reduced hyperemia and swelling although not significantly.

## P35 The difference of posterior tibial slope angle on short-term clinical effect after posterior-stabilized total knee arthroplasty

### Wassapol Rerksanan, Wasin Wichitpreeda, Viroj Larbpaiboonpong

#### Department of Orthopedic, Police General Hospital, Bangkok, Thailand

##### **Correspondence:** Wassapol Rerksanan (whatwai@hotmail.com)

*Arthroplasty 2024*, **6(Suppl 1):**P35

**Objective**

One of the most important factors that affect the outcomes of total knee arthroplasty (TKA) is alignment. Surgeons always keep post-operative alignment back to normal for each patient to restore their knee kinematics. However, there is a lack of study about sagittal alignment especially posterior tibial slope (PTS). At present, the proper of PTS is still controversial. Many finite element and cadaveric studies confirm that knee kinematics also depend on the changing of PTS in Posterior-stabilized TKA (PS-TKA). The purpose of this study was to evaluate the effect of changing PTS on short-term clinical outcomes in PS-TKA.

**Method**

A retrospective cohort study in 42 knees who underwent PS-TKA. Divide into 2 groups, first for patients who change PTS to 3 degrees or higher and another group for patients who change PTS to less than 3 degrees. Pre-operative and post-operative Knee ROM, VAS, Coronal alignment, PTS, and WOMAC score of each patient were collected the day before surgery and at 1-year follow-up.

**Result**

All patients got better clinical outcomes post-operative. There is no significant in the preoperative characteristic between both groups. Although patients who changing of PTS less than 3 degrees had no significant change in VAS (1.6 (SD 0.3) vs 1.8 (SD 0.5), *P* = 0.89), knee ROM (118 (SD 10) vs 114 (SD 8), *P* = 0.57), and WOMAC score (18.7 (SD 4.6) vs (19.4 SD 5.1), *P* = 0.64).

**Conclusion**

Changing PTS did not affect the postoperative ROM and clinical outcomes of PS-TKA. The less changing of PTS may be a proper option for adjusting PTS in PS-TKA. 

## P36 An S-curved skin incision to avoid injury to the infrapatellar branch of the saphenous nerve (IPBSN) during total knee arthroplasty (TKA): a cadaveric study

### Vorathep Wangtrakunchai, Nitchanant Kitcharanant, Warakorn Jingjit, Kasisin Klunklin, Sakkadech Limmahakhun

#### Department of Orthopedic, Faculty of Medicine, Chiang Mai University, Chiang Mai, Thailand

##### **Correspondence:** Vorathep Wangtrakunchai (wang.vorathep@gmail.com)

*Arthroplasty 2024*, **6(Suppl 1):**P36

**Background**

Total knee arthroplasty (TKA) is a widely performed surgical intervention for managing knee osteoarthritis, but it is often accompanied by complications such as anterior knee pain and numbness. These complications are frequently attributed to damage incurred by the infrapatellar branch of the saphenous nerve (IPBSN).

**Objective**

This research endeavors to thoroughly examine the anatomy of the IPBSN and to develop an S-curved skin incision technique that minimizes the risk of nerve injury during TKA procedures.

**Methods**

Conducted in two distinct phases, this study first involved the detailed dissection of 20 cadaveric knees using surgical loupes to elucidate the anatomy and variation of the IPBSN. In the subsequent phase the S-curved incision technique on 10 additional cadaveric knees during medial parapatellar arthrotomy, specifically designed to preserve the IPBSN.

**Results**

Investigation revealed variable exit patterns of the IPBSN from the adductor canal, with anterior, penetrating, posterior, and pes anserinus types observed in 30%, 50%, 15%, and 5% of the specimens, respectively. The average distance of the IPBSN from the medial border of the patella at the mid-patellar level was measured to be 10.65 ± 4.35 cm. The nerve demonstrated branching into single, double, and triple branches in 20%, 60%, and 20% of cases, respectively. The mean distances from the main branch of the IPBSN to the inferior border of the patella at the midline, and from the tibial tuberosity to the distal end of the nerve, were recorded as 2.33 ± 0.92 cm and 1.03 ± 0.38 cm, respectively. The S-curved incision was found to effectively prevent IPBSN injury while providing sufficient surgical exposure for TKA, surpassing the traditional midline incision in efficacy (Figs 1–3).

**Conclusions**

The S-curved incision technique represents a promising surgical modification for TKA, potentially diminishing the incidence of anterior knee pain or numbness by ensuring the preservation of the IPBSN. This study advocates a paradigm shift in surgical techniques to optimize postoperative outcomes through enhanced nerve conservation.

**Fig. 1 (Abstract P36):**
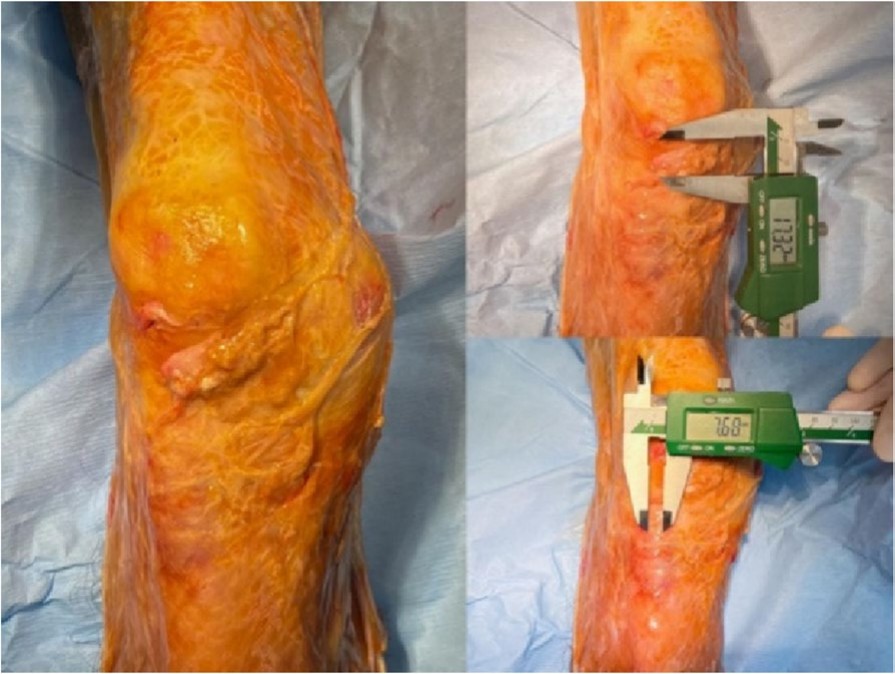
Anatomical Findings and Measurements of the Infrapatellar Branch of the Saphenous Nerve (IPBSN)

**Fig. 2 (Abstract P36):**
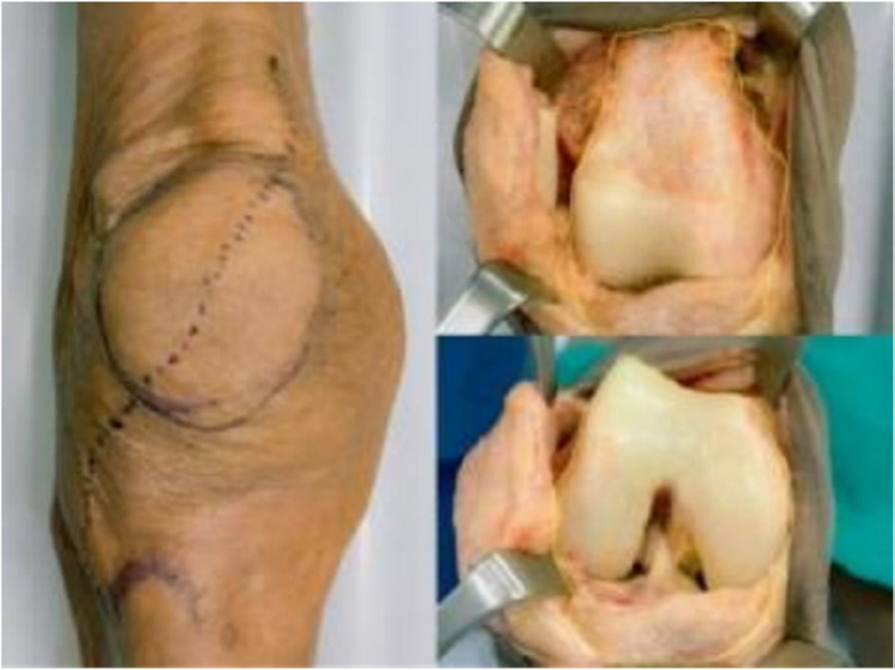
S-curved Skin Incision (length 15 cm): The proximal incision is located at the medial border of the patella and then passes through the midpoint of the patella. The distal incision starts from the midpoint of the patella and extends 2 cm laterally towards the tibial tubercle, followed by a medial parapatellar approach to expose the bone. The ImageJ software was used to measure the extent of bone exposure in both the extended position and at 90-degree flexion

**Fig. 3 (Abstract P36):**
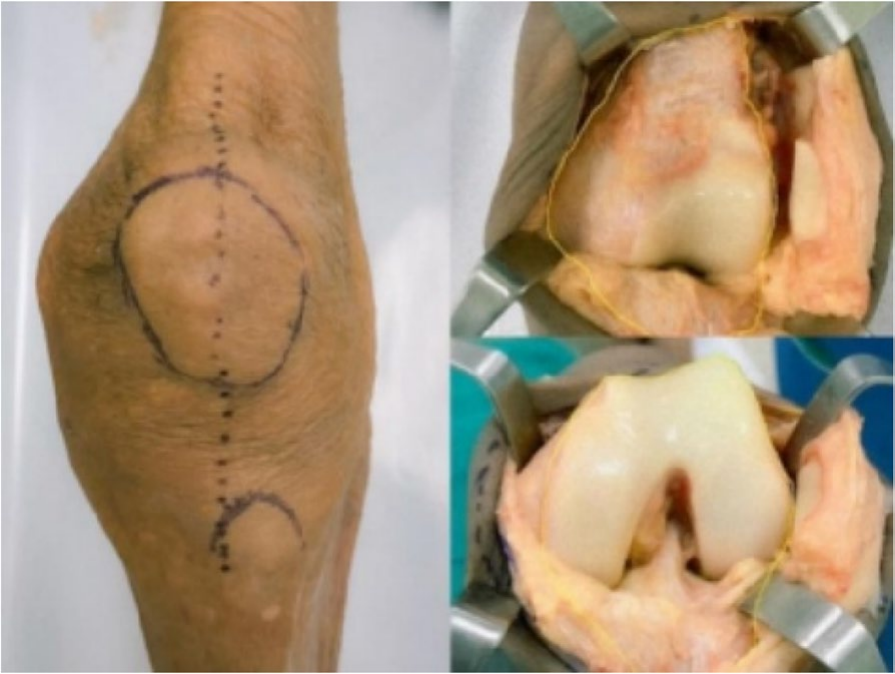
Midline Skin Incision (length 15 cm): Followed by a medial parapatellar approach to expose the bone. The ImageJ software was utilized to measure bone exposure in both the extended position and at 90 degrees of flexion

## P37 Early postoperative outcomes in bilateral simultaneous concurrent versus unilateral total knee arthroplasty: a comparative study

### Parisak Phungoen, Chayut Chaiperm

#### Department of Orthopedics, Bhumibol Adulyadej Hospital, Directorate of Medical Services, Royal Thai Air Force, Bangkok, Thailand

##### **Correspondence:** Parisak Phungoen (parisak5@hotmail.com)

*Arthroplasty 2024*, **6(Suppl 1):**P37

**Introduction**

While studies have compared unilateral TKA (UTKA) to simultaneous sequential bilateral TKA, there remains a gap in the literature regarding the outcomes of simultaneous concurrent bilateral TKA (SCBTKA), where both knees are operated on at the same time. This innovative approach could potentially offer distinct benefits or pose unique challenges compared to UTKA. This study aims to fill this gap by comparing the early postoperative outcomes of SCBTKA with those of UTKA. 

**Methods**

This retrospective analysis included all patients who underwent SCBTKA and UTKA between January 2022 and December 2023. Key outcomes, including length of stay, operative time, blood transfusion requirements, and in-hospital complications, were compared between the two groups. 

**Results**

The analysis included 63 (41.7%) SCBTKA and 88 (58.3%) UTKA patients. The median length of stay was similar between groups (SCBTKA: 7 days; UTKA: 6 days, *P* = 0.139). Postoperative hemoglobin levels were lower in the SCBKA group (10.17 ± 1.13 g/dL vs. 10.90 ± 1.50 g/dL, *P* = 0.001), with a greater decrease from baseline (ΔHb: −2.17 ± 0.94 vs. −1.72 ± 0.95, *P* = 0.005). Blood transfusion requirements were higher in the SCBTKA group (*P* = 0.003). Operative time was longer for SCBTKA (134.70 ± 30.73 min vs. 111.47 ± 30.36 min, *P* < 0.001). Early complications were rare, with no significant difference between groups (*P* = 0.417). 

**Conclusion**

BSCTKA is safe with similar early postoperative outcomes and discharge times compared to UTKA. Larger cohorts and longer follow-ups are needed to evaluate long-term functional outcomes.

## P38 Comparison of outcomes between multimodal intraosseous femoral injection and multimodal intraosseous tibial injection, a randomized controlled trial in simultaneous bilateral total knee arthroplasty patients

### Tanahem Wijit, Patcharavit Ploynumpon, Thakrit Chompoosang

#### Hip & Knee Arthroplasty Unit, Department of Orthopedic Rajavithi Hospital, Collage of Medicine Rangsit University, Bangkok, Thailand

##### **Correspondence:** Tanahem Wijit (troy.erdos@gmail.com)

*Arthroplasty 2024*, **6(Suppl 1):**P38

**Purpose**

Periarticular multimodal analgesia is a standard pain relief method for total knee arthroplasty (TKA) patients. Recent studies have demonstrated that intraosseous injection of pain relievers and antifibrinolytic agents provides statistically significant reductions in pain and blood loss. This study aimed to compare the outcomes between multimodal intraosseous femoral and tibial injections inpatients undergoing bilateral TKA.

**Methods**

A double-blind, randomized controlled trial was performed on 40 patients. Patients received multimodal intraosseous injections at either the femur or tibia for each TKA, with the site alternating between groups. Postoperative outcomes assessed included Visual Analog Scale (VAS) pain score, amount of painkiller used after surgery, range of motion, side effects, and complications.

**Results**

Among 40 TKA patients, no significant differences were found in VAS pain scores at various time points (12, 24, 48 hours, and 2 weeks postoperative). However, the femoral site demonstrated significantly lower postoperative blood loss as measured by drainage (289.6 vs. 350.4 mL, *P* < 0.001) and total blood loss (303.5 vs. 365.4 mL, *P* < 0.001). No significant differences were observed in the amount of painkiller used after surgery or postoperative range of motion. No side effects or complications were reported.

**Conclusion**

Multimodal intraosseous injection at the femoral site appears as effective for pain management as the tibial site but results in significantly reduced postoperative blood loss in patients undergoing TKA. Further study is required to explore the long-term benefits and confirm the safety profile of this technique.

## P39 Comparison of clinical outcomes between lateral mobile bearing UKA and lateral fixed bearing UKA in Valgus knee osteoarthritis patients

### Jatupong Taweepreda, Boonchana Pongcharoen

#### Department of Orthopedic, Faculty of Medicine, Thammasat University, Bangkok, Thailand

##### **Correspondence:** Jatupong Taweepreda (jatupongjt@gmail.com)

*Arthroplasty 2024*, **6(Suppl 1):**P39

**Background**

Valgus knee osteoarthritis (OA) patients mostly undergo total knee arthroplasty, clinical outcomes are excellent but take time to recover and limit daily life function in young and obese patients, the Lateral unicompartmental knee arthroplasty (UKA) is an option to restore function in patients with lateral compartment knee osteoarthritis, The aims this study was to compare functional outcome of patients following mobile bearing lateral UKA (MB-UKA) and fixed bearing lateral UKA (FB-UKA) in valgus knee OA.

**Materials and Methods**

Retrospectively cohort therapeutic study with regards to age, gender, and body mass index (BMI) between fixed and mobile lateral UKA. Functional outcome was measured using the Oxford Knee Score (OKS), Forgotten Joint Score (FJS), and range of motion (ROM). Complications and revisions were recorded.

**Result**

A cohort of 47 patients, mobile bearing (*n* = 17), fixed bearing (*n* = 30) lateral UKA in valgus OA knee, Mean follow-up 1 year) range 3 months–2 years, Age, gender, BMI is not significant between the group, OKS at 1-year follow-up in MB-UKA 46.7 ± 2.2 (41–48), FB-UKA 44.3 ± 4 (31–48), *P*-value 0.06, FJS at 1 year follow up in MB-UKA 98.5 ± 5.1 (79–100), FB-UKA 99.5 ±1.6 (93.75–100), *P*-value 0.33, post-op ROM in MB-UKA 120.0 ± 5.0 (110–130), FB-UKA 120.2 ± 6.5 (110–130) *P*-value 0.93, we not seen any complications in both groups.

**Conclusions**

Lateral mobile bearing UKA and lateral fixed bearing UKA have shown similar clinical outcome.

